# Is Cobalt Joining the σ‑/π-Hole Crown? Exploring Noncovalent Interactions in Cobalamin

**DOI:** 10.1021/jacsau.5c00955

**Published:** 2025-10-09

**Authors:** Sergi Burguera, Antonio Bauzá

**Affiliations:** 16745 Universitat de Les Illes Balears, Ctra. de Valldemossa, Km. 7.5, Palma de Mallorca, Islas Baleares 07122, Spain

**Keywords:** noncovalent interactions, σ-/π-hole chemistry, PDB survey, cobalamin, theoretical study

## Abstract

Herein, the σ-/π-hole chemistry of Co within a biological framework was computationally studied for the first time using Cobalamin (Cbl) as a case study. Starting with a Protein Data Bank (PDB) survey, 111 structures involving Cbl were found, 67 of which presented a noncovalent interaction (NCI) involving the Co atom and either substrate/product molecules, protein residues, or artificial ligands. These data were statistically analyzed to unveil trends regarding the abundance and structural features of the L···Co interaction (L = C, N, and O), and several selected examples were investigated at the BP86-D3/def2-TZVP level of theory. In addition, a computational study revealed the existence of a σ-hole complex involving the 5,6-dimethylbenzimidazole (DMB) group as a stabilizing prestate prior to Co coordination. This finding was further assessed by the identification of several local noncovalent energy minima based on σ-hole interactions using NCH, NH_3_, OCHNH_2_, and O­(CH_3_)_2_ as electron donor molecules, which were characterized using quantum chemistry tools. We believe that the results reported herein will have a significant impact on the fields of chemical biology, supramolecular chemistry, and biotechnology by describing for the first time σ-/π-hole interactions involving Co in biological systems, which are often overlooked by traditional coordination chemistry.

## Graphical abstract

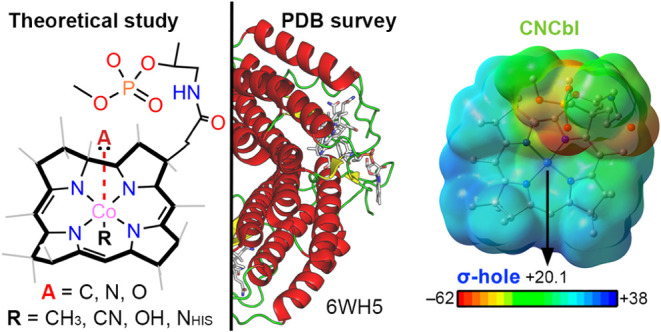

## Introduction

### Cobalamin Biological Roles and Main Structural Features

Cobalt is a relatively rare element in the Earth’s crust but is essential for life.^1–3^ It exists in various oxidation states, with the most biologically relevant being Co­(I), Co­(II), and Co­(III). Co biological role is almost exclusively linked to Cobalamin (Cbl, also known as vitamin B_12_) chemistry. Since the initial isolation of crystalline vitamin B_12_ (cyanocobalamin) during the past century,^4,5^ much work has been devoted to understanding its role in cellular metabolic pathways,^6–8^ DNA synthesis and methylation,^9,10^ as well as in amino acid and fatty acid biosynthesis,^11–13^ all of which are key areas of human metabolism. In this context, Cbl is a coenzyme in two primary enzymatic pathways in humans: methionine synthase (using methylcobalamin) and methylmalonyl-CoA mutase (using adenosylcobalamin). The first metabolic route catalyzes the conversion of homocysteine to methionine, a crucial reaction for DNA synthesis and methylation.^14^ In this process, methylcobalamin transfers a methyl group, facilitating the production of methionine, which subsequently generates *S*-adenosylmethionine (SAM), a universal methyl donor in numerous biochemical reactions. Without this reaction, homocysteine accumulates, leading to cardiovascular and neurological disorders.^15,16^ In fact, a deficiency of vitamin B_12_ disrupts this pathway, contributing to diseases such as megaloblastic anemia and neurological impairment. The second metabolic pathway uses adenosylcobalamin as a coenzyme to catalyze the rearrangement of methylmalonyl-CoA to succinyl-CoA, a critical step in amino acid and fatty acid metabolism.^17,18^ Deficiencies in vitamin B_12_ typically result in methylmalonic acid accumulation, leading to metabolic disorders such as methylmalonic acidemia.^19^

On the other hand, in some prokaryotes, Co is also present in metalloenzymes, although its functions are limited compared to its role in vitamin B_12_ biochemistry. For instance, it plays a role in the dehydration reaction of 1,2-diols and glycerol, which are important chemical species in bacterial fermentation and anaerobic metabolism,^20^ as well as in the cleavage of the C–N bond in ethanolamine, demonstrating Cbl’s role in bacterial survival and adaptation.^21^ Furthermore, reductive dehalogenases are a specialized class of Cbl-dependent enzymes found in some bacteria, catalyzing the removal of halogenated organic pollutants, thus playing an essential role in bioremediation processes.^22^ Lastly, in archaea, ribonucleotide reductases rely on Cbl derivatives to carry out DNA biosynthesis by catalyzing the conversion of ribonucleotides into deoxyribonucleotides,^23^ thereby highlighting Co’s crucial role in DNA synthesis and replication.

These metabolic pathways are influenced by (i) the capacity of the Co center to coordinate/release small chemical species and (ii) the stabilization of prereaction/postreaction states of the protein. In this regard, noncovalent forces can play a crucial role, and thus, the ability of the Co center to behave as a noncovalent Lewis acid might be key to controlling substrate positioning, barrier modulation, cofactor activation, and protein conformational stabilization. Hence, the presence of electrophilic regions over the Co atom can potentially be related to its inherent biological functions.

From a structural perspective, Cbl is one of the most complex biomolecules, distinguished by its central Co ion bound to a corrin ring, a tetrapyrrolic macrocycle similar to the porphyrin ring found in the heme group. However, it is less symmetric and allows greater flexibility in coordination chemistry, making Cbl distinct from heme-based systems.^24^ A maximum of six ligands bind to the Co center, four of them being N atoms from the corrin ring. In addition, from the two axial positions, one of them is typically occupied by different ligands that define the functional identity of Cbl in one-carbon metabolism. These include −CN (cyanocobalamin, CNCbl), −OH (hydroxocobalamin, OHCbl), −CH_3_ (methylcobalamin, MeCbl), and 5′-deoxyadenosyl (adenosylcobalamin) groups. The other axial position is usually occupied by a 5,6-dimethylbenzimidazole (DMB) phosphoribosyl moiety, which is further connected to the corrin ring through one of its propionamide side chains. In all Cbl derivatives, the coordination of the Co ion adopts a “base-on” configuration, where the N atom of DMB is directly coordinated to the Co center in one of the axial positions. However, when Cbl is bound to enzymes, such as methionine synthase and methylmalonyl-CoA mutase, a conformational rearrangement occurs, leading to the replacement of the DMB moiety by an HIS residue from the protein. This alternative coordination state is termed the “His-on” position. Studies have demonstrated that the transition from the “base-on/His-off” conformation to the “base-off/His-on” conformation plays a critical role in the catalytic function of these enzymes.^25^

### Cbl Noncovalent Chemistry Can Be Understood through σ-/π-Hole Interactions

While much is known regarding this crucial metal–organic assembly in terms of Co oxidation states and coordination chemistry, the ability of Co to participate as a noncovalent Lewis acid has not yet been investigated within this framework. In this regard, we have recently explored the noncovalent chemistry of first-row transition metal elements using porphyrin (ppy) as a molecular template, demonstrating that Co can indeed behave as a noncovalent Lewis acid when placed in the appropriate chemical context.^26^ This behavior is due to the presence of low electron density areas over the Co atom (exhibiting a positive electrostatic potential) perpendicularly located to the ppy molecular plane, also known as π-holes.^27–29^ The presence of these electropositive π-hole regions in Cbl is observed in a tetracoordinated Co environment (square planar geometry; see Figure 1a), where the Co atom usually exhibits a +2 oxidation state, enabling favorable interactions with Lewis bases through both sides of the molecular plane. The π-hole complexes established are also known as semicoordination bonds,^30,31^ which are related to associative substitution mechanisms involving organometallic coordination complexes.

**Figure 1:**
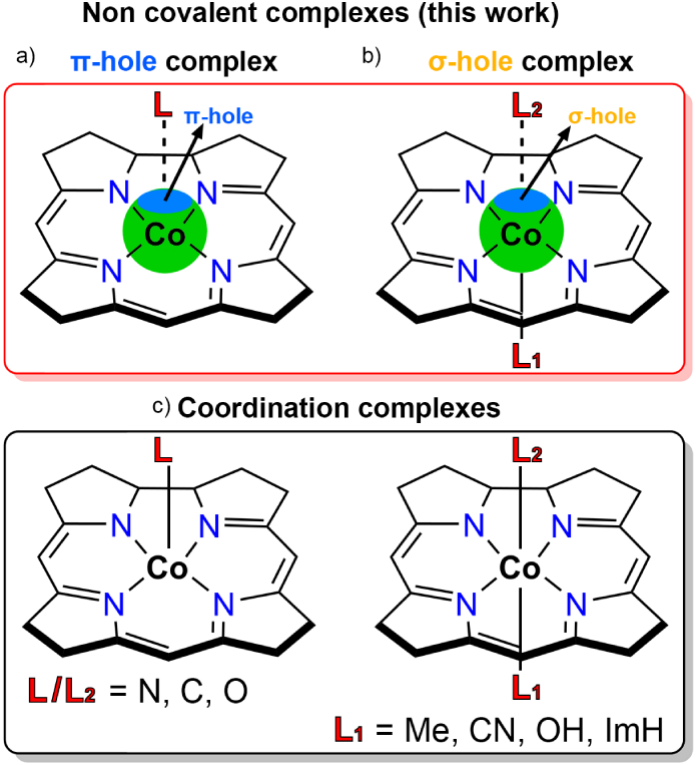
Schematic representation of the difference between noncovalent π-hole (a) and σ-hole (b) L/L_2_···Co bonds and L/L_2_–Co coordination bonds (c) involving a corrin ring. ImH stands for imidazole.

On the contrary, in Cbl derivatives (MeCbl, CNCbl, and OHCbl) where the Co ion exhibits a penta-coordinated environment (square pyramidal geometry, see Figure 1b), a σ-hole^32^ (a low electron density site exhibiting a positive electrostatic potential located along the vector of the Co–L_1_ coordination bond) is observed, which assists in the stabilization of the second axial ligand (L_2_). This is due to the blockage of one of the two Co axial coordination positions, which typically leads to a change in the oxidation state from +2 to +3, thus affecting the Lewis acidity of the Co center (see the electrostatic potential surface analyses below) and directing the interaction of L_2_ with the Co atom.

The presence of these electropositive regions over the Co atom acts as a link between the classical Co biochemistry and the novel supramolecular bonds involving the d-block of elements,^33–39^ which share the same physical principles as those noncovalent interactions (NCIs) involving the main group chemistry elements,^40–44^ such as the Halogen Bonding (HalB) interaction.^45,46^ The inclusion of Co and Cbl into the σ-/π-hole chemistry is important not only to expand the σ-/π-hole family of interactions but also to further understand the noncovalent chemistry regarding this crucial organometallic complex, since these interactions could have deep implications related to Cbl’s structure and functionality, for instance by regulating the strength of the DMB···Co interaction or even substrate/product molecular recognition events.

In the present study, we inspected the Protein Data Bank (PDB) to investigate the ability of Cbl to participate in σ-/π-hole chemistry. As a result, we obtained statistical data regarding L···Co intermolecular distances and L···Co–N angle distributions (L = C, N, and O). Additionally, 10 representative PDB structures were selected, and the strength of the L···Co interaction was evaluated at the BP86-D3/def2-TZVP level of theory. Furthermore, we investigated the influence of N_DMB_···Co interactions on Cbl, MeCbl, CNCbl, OHCbl, as well as imidazole-coordinated Cbl (ImHCbl) stability, resulting in the identification of noncovalent local energy minima in most cases, which were characterized as σ-hole complexes. The existence of Co noncovalent bonds based on these NCIs was further assessed through a computational study (also at the BP86-D3/def2-TZVP level of theory) involving several N, C, and O donor Lewis bases (NCH, NH_3_, OCHNH_2_, and O­(CH_3_)_2_), which allowed us to characterize the physical nature of these NCIs.

## Results and Discussion

### Understanding the σ-/π-Hole Donor Ability of Cbl

Prior to investigating the PDB, we computed the Molecular Electrostatic Potential (MEP) surfaces of several theoretical models involving Cbl and its main derivatives (Cbl, MeCbl, CNCbl, OHCbl, and ImHCbl), as shown in Figure 2. As noted, in MeCbl, CNCbl, and OHCbl derivatives, the Co­(III) ion exhibited a positive electrostatic potential region along the extension of the Co–Me/CN/OH coordination bond, which resembles a classical σ-hole, with values of +11.9, +20.1, and +26.3 kcal·mol^–1^, respectively. These values followed the expected trend regarding the electron-donating/electron-withdrawing ability of the ligand moieties, with the −CH_3_ and −OH groups acting as σ-donor/acceptor ligands, while in the case of the −CN group, the π-system acts as a π-donor and slightly counterbalances the σ-acceptor ability of this ligand. On the other hand, in the ImHCbl derivative, a slightly positive electrostatic potential value along the Co–N coordination bond was obtained (+2.1 kcal·mol^–1^), likely due to the greater π-donor ability of the ImH moiety compared to the rest of the ligands used. Lastly, in Cbl we found a positive MEP value over the Co­(II) atom, which extended above and below the corrin ring plane, thus resembling a π-hole, in agreement with the results obtained in ppys investigated by us in our previous study.^26^ Taken all together, the presence of a coordinated axial ligand converts the Co π-hole into a σ-hole and can influence the σ-hole donor ability of Co by tuning its electrostatic potential surface value. This might be important since the presence of one coordinated ligand to the Co center is often used to regulate the interaction with the second axial ligand, which is a crucial step in the catalytic cycle of Cbl.

**Figure 2:**
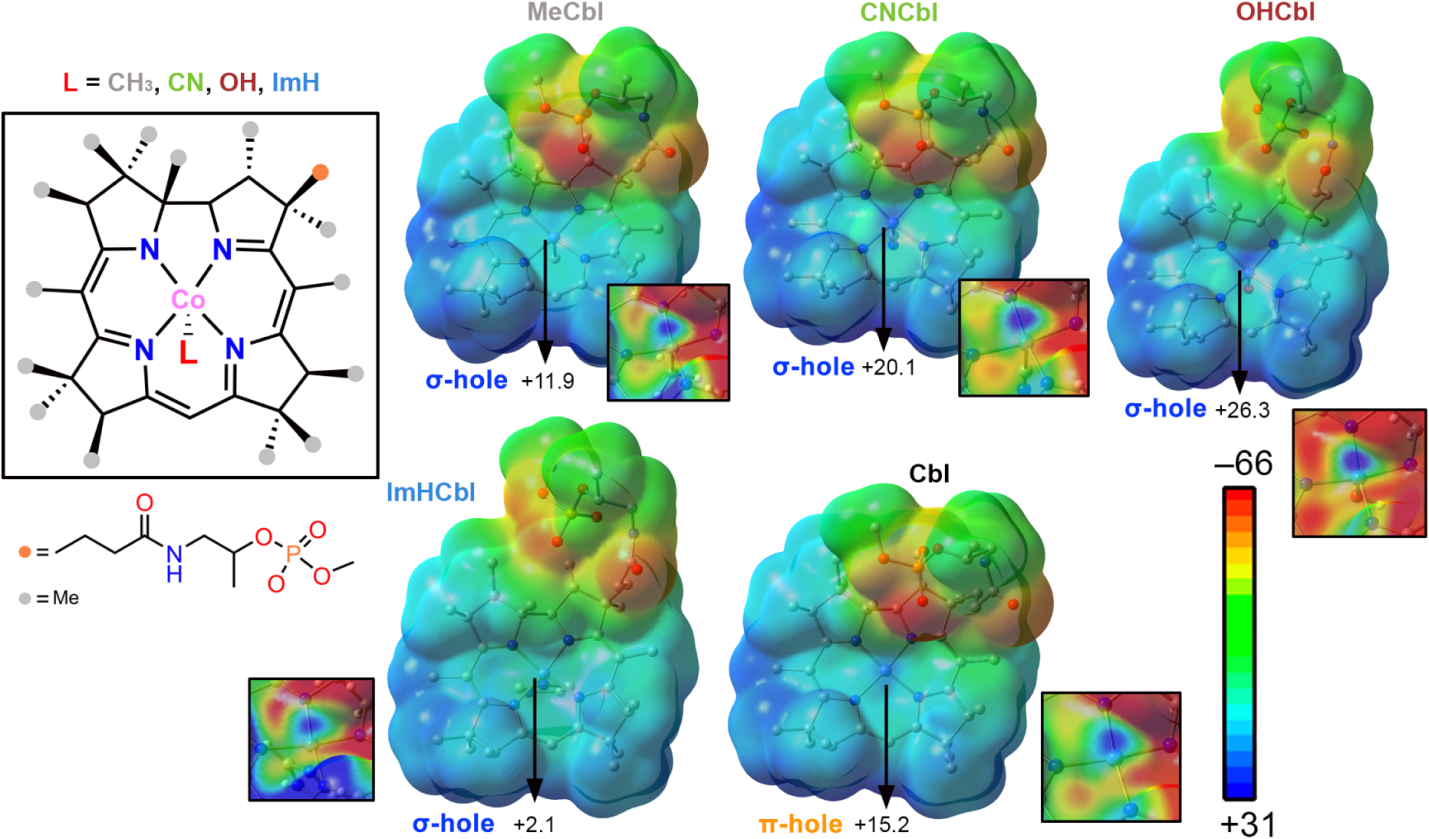
MEP surfaces of Cbl, MeCbl, CNCbl, OHCbl, and ImHCbl. Energy values at specific points on the surfaces are given in kcal·mol^–1^ (isosurface contour = 0.001 au). The MEP scale was tweaked to show more clearly the presence of a σ-/π-hole over the Co atom (see the squared parts of the figure). H atoms were omitted for the sake of clarity.

### Statistical Analyses of the PDB Data

Having elucidated both the σ- and π-hole donor ability of Cbl and its derivatives, we conducted a PDB inspection to analyze L···Co (L = C, N, or O) interactions. During our search, those contacts below 2.5 Å were considered as coordination bonds, while those between 2.5 Å and the sum of the Co + C/N/O radii + 0.5 Å were considered noncovalent in nature (this includes both semicoordination bonds and purely noncovalent interactions; see the PDB survey details in the Supporting Information for more details).

As a result, we found a total of 111 structures, and in 67 of them, at least one of the two corrin axial ligands was establishing a semicoordination/noncovalent contact with the Co center. In Figure 3a, a schematic representation of the geometrical parameters used during the search is shown, with d_1_ and d_2_ representing the two L···Co distances and ∝̅_1_ and ∝̅_2_ the average L···Co–N angles (see the Computational Methods section in the Supporting Information for more details).

**Figure 3:**
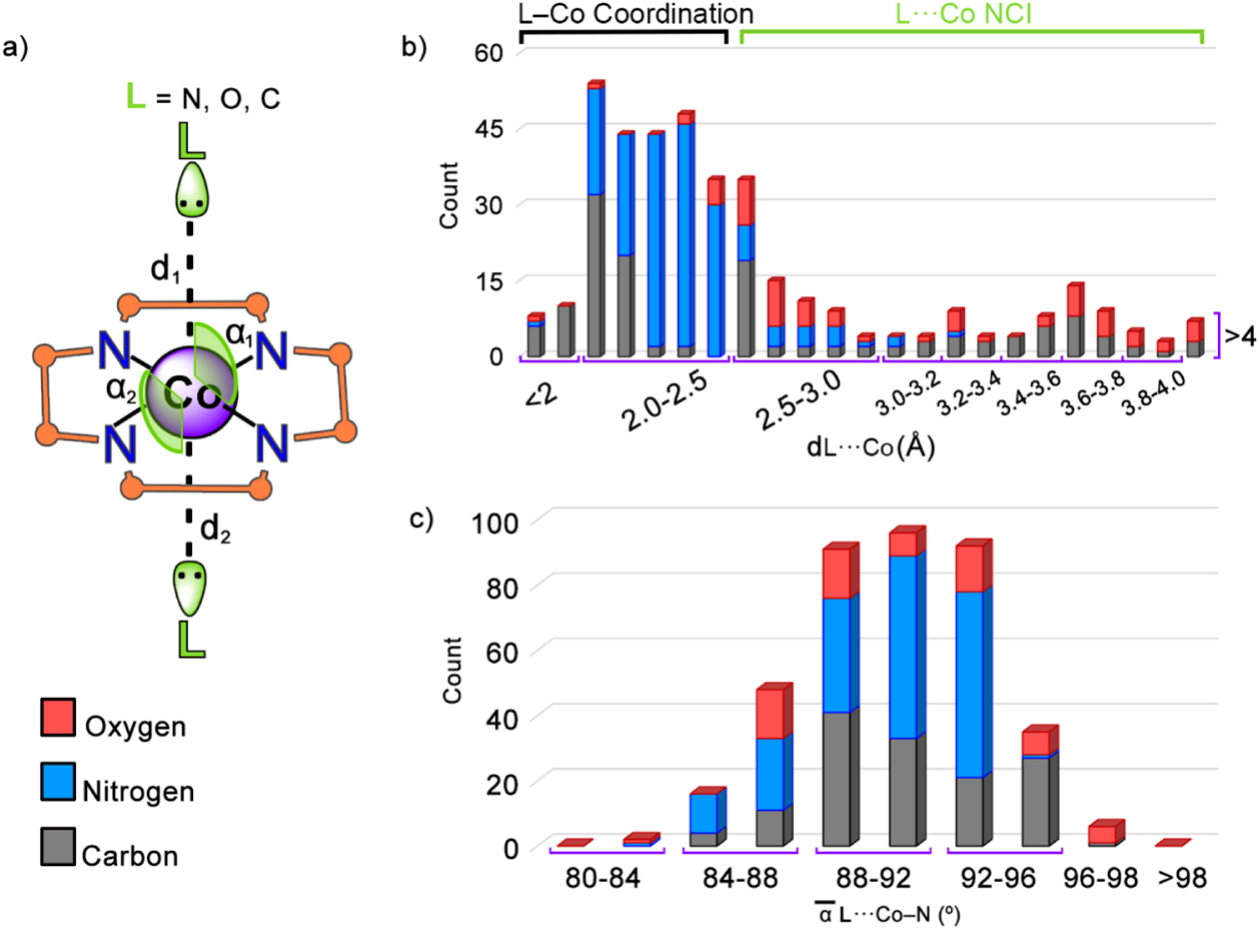
(a) Schematic representation of the distance (d_1_ and d_2_) and average angle (α_1_ and α_2_) parameters used during the PDB search. (b and c) Graphical representations of the PDB hit count vs the L···Co (L = C, N, and O) distance (dCo···A, in Å) and the average L···Co–N angle (α̅ L···Co–N, in °).

In Figure 3b the results regarding the abundance of coordination and noncovalent L···Co contacts are shown. Specifically, we found a higher abundance of L–Co coordination contacts (252) compared to those involving an NCI/semicoordination bond (157), as expected. N donor species (colored in blue) were the most abundant ligands due to the presence of DMB and HIS moieties, followed by C atoms (colored in gray) from either methanide moieties or substrate molecules (e.g., 5′-deoxyadenosyl), and lastly, by O atoms (colored in red), which mainly belong to water molecules. On the other hand, among the 157 noncovalent contacts (these were considered from 2.5 Å of L···Co distance based on the different element radii involved; see Supporting Information for more details), most of them involved either a C (66) or an O (55) atom from a protein amino acid (e.g., PHE, THR, or SER) or a water molecule, while a smaller portion involved a N atom from a HIS residue (36). From these, 10 examples were selected, and the strength of the L···Co interaction as well as its plausible biological role were computationally investigated (see below).

On the other hand, regarding the average L···Co–N angle values, we found that in most of the structures, these values were between 84 and 92°, in line with an axial ligand interaction. Many of them involved a N atom, likely due to the higher abundance of Co–N coordination bonds (involving DMB or HIS moieties) compared to Co–C and Co–O coordination bonds. On the other hand, the average angle values for O spanned between 80 and 98°, suggesting particular flexibility when approaching/binding Cbl. Lastly, in the case of C the behavior observed was similar to that for N, where most of the structures exhibited C···Co–N angles comprised between 84° and 96°. In Figure S1, we found a linear relationship between the two L···Co distances (d_1_ and d_2_), thus indicating that the formation of one of the two axial L···Co bonds (either a π-hole complex or a coordination bond) serves as a spatial guide for the second axial L···Co interaction (being either a σ-hole complex or a second coordination bond). The same behavior was found when comparing the two average L···Co–N angles (∝̅_1_ and ∝̅_2_), thus suggesting a linear dependency between them upon formation of the octahedral Co environment.

If the data presented in these plots is analyzed separately for C, N, and O, we found several interesting aspects. Concretely, in Figure 4 we plotted the relationships between the average L···Co–N angle (∝̅ L···Co–N, in °) and the L···Co distance (in Å) for all three donors. We found two clearly differentiated regions in the case of C: one located between 1.5 and 2.5 Å spanning from 88 to 96°, which corresponds to the formation of Co–C coordination bonds, and another one comprised between 2.5 and 4.0 Å and between 87 and 92°, which belongs to the formation of C···Co noncovalent complexes. On the other hand, in the case of N we found that the data were much more concentrated in one specific region, located between 2.0 and 2.5 Å and between 90 and 94°, which agreed with the higher number of N···Co coordination bonds as shown in Figure 3b. In addition, a small fraction of hits was scattered above 2.5 Å, corresponding to N···Co noncovalent interactions. Furthermore, in the case of O, we found a cluster of hits between 2.5 and 3.0 Å and between 92 and 96°, while some data were scattered between 2.5 and 4.5 Å, exhibiting a small cluster located around 3.6 Å and 88°, mainly corresponding to noncovalent O···Co contacts. Lastly, in Figure 4 we also included the regression lines for all three graphics, including a confidence interval (shadowed region). In the case of N, the regression line indicates that the relationship between the N···Co distance and the N···Co–N angle remains approximately the same across coordinated and semicoordinated/noncovalent contacts, thereby sharing similar directionality (probably conditioned by the vast number of N–Co coordination bonds compared to noncovalent N···Co contacts). On the other hand, we found that for C and O, this relationship decreases, meaning that the C/O···Co–N angle lowers while the C/O···Co distance increases, indicating a slightly different spatial approach to the Co center between coordinated and noncovalent contacts.

**Figure 4:**
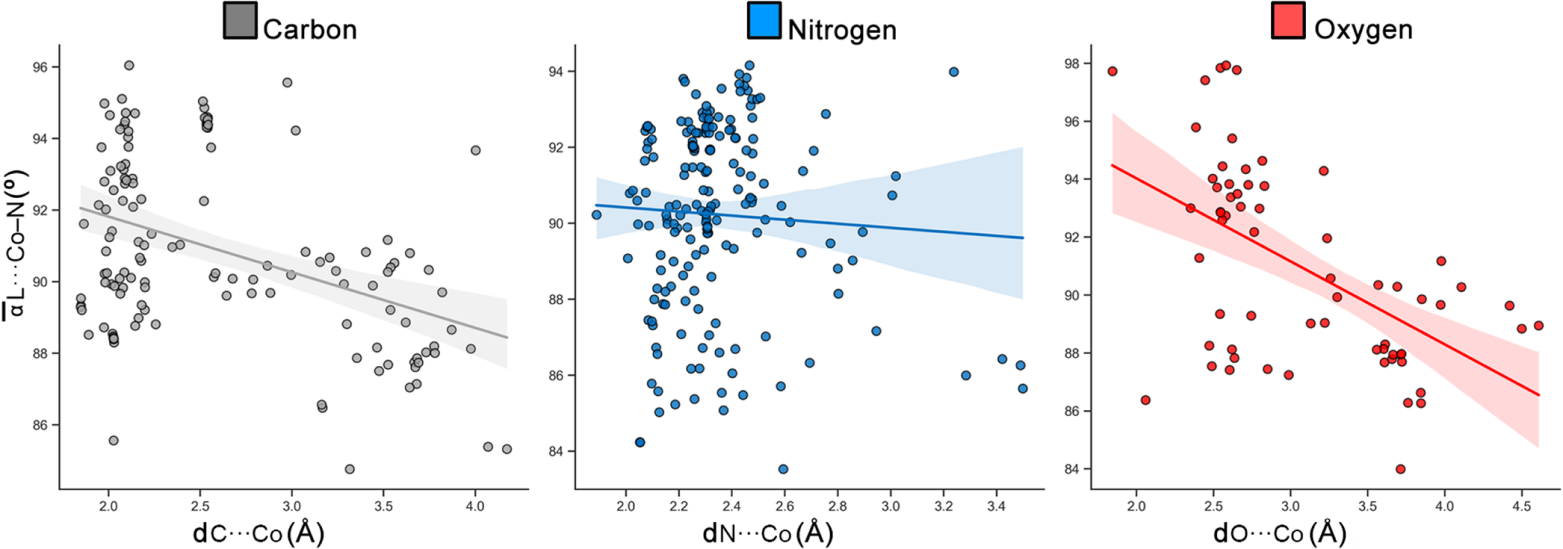
Individual scatter plots of the L···Co (L = C, N, and O) distance (dL···Co, in Å) vs the average L···Co–N angle (α̅L···Co–N, in °). The regression lines for all three graphics, including a confidence interval (shadowed region), are shown, indicating that the relationship between the distance and angle decreases for C and O when comparing coordination and semicoordination/noncovalent contacts, while for N it remains around a constant value.

### Theoretical Calculations on Selected PDB Structures

From these results, we selected 10 PDB structures involving different ligands and codependent enzymes and computed the interaction energy value of the L···Cbl interaction to provide a general overview of the Lewis base···Co strength (see Computational Methods and Table S1 in the Supporting Information for further details). In Figure 5, three of the calculated examples are highlighted, involving human transcobalamin,^47^ cobalamin adenosyltransferase,^48^ and human methylmalonyl-CoA mutase enzymes.^49^ The first selected example (see Figure 5a) encompasses the X-ray crystal structure of transcobalamin (TC), a cobalamin transporter enzyme solved at 3.20 Å resolution. TC exhibits two main domains: (i) an N-terminal ″α-domain” dominated by α-helical motifs (colored in red in Figure 5a) and (ii) a C-terminal ″β-domain” where β-strands predominate (colored in yellow in Figure 5a).

**Figure 5:**
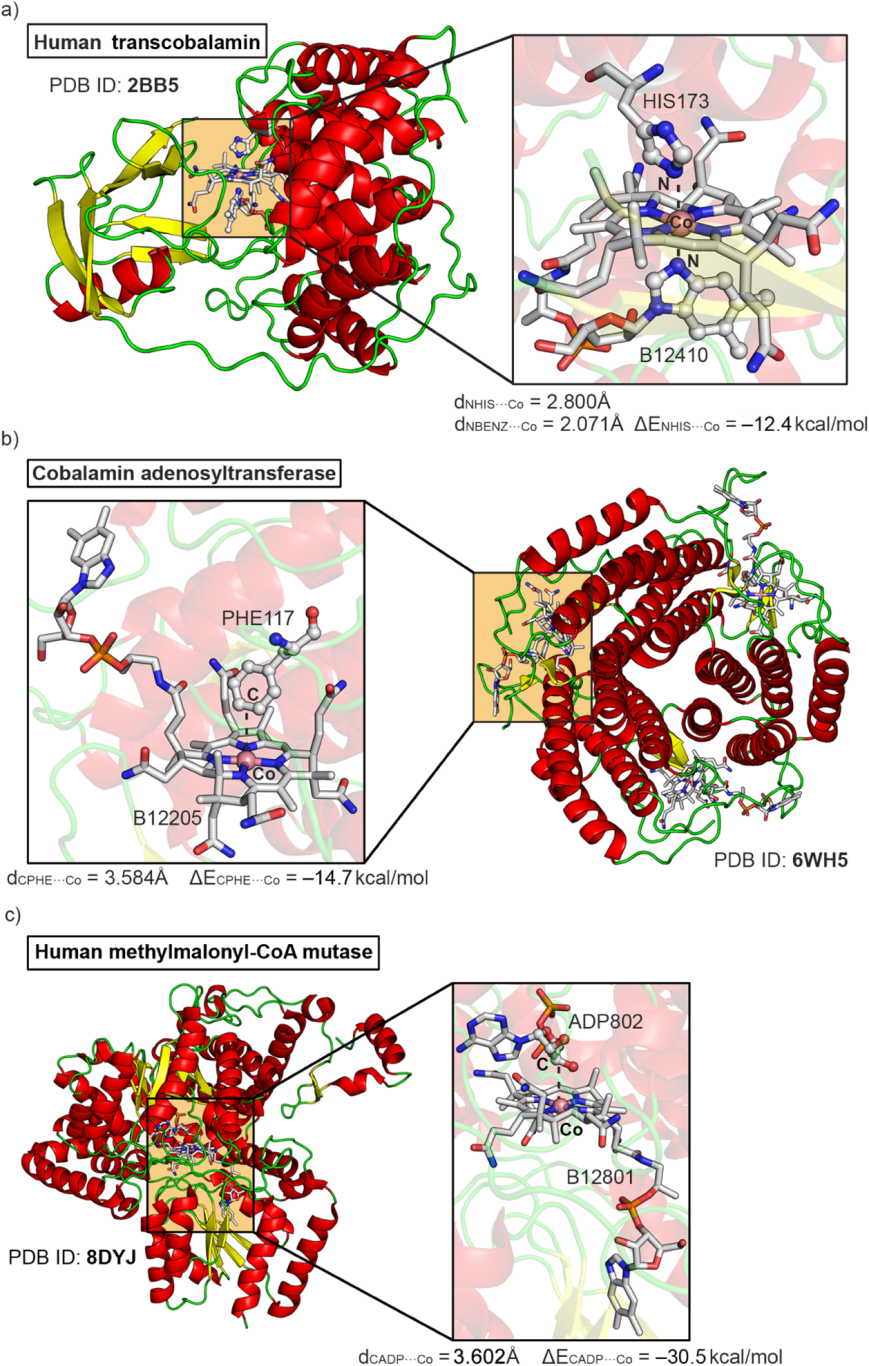
Partial views of the X-ray crystal structures corresponding to (a) Human transcobalamin transporter (PDB ID: 2BB5), (b) Cobalamin adenosyltransferase (PDB ID: 6WH5), and (c) Human methylmalonyl-CoA mutase (PDB ID: 8DYJ). The noncovalent Co···C/N interaction is magnified within the squared parts of the figure, with an indication of the C/N···Co distance and interaction energy value.

In this structure, a Cbl in the “base-on” conformation (with the Co ion coordinated by the DMB group) was located between the two domains, with the plane of the corrin ring approximately perpendicular to the domain interface (exhibiting a square pyramidal coordination environment). Consequently, two axial N···Co interactions were observed, involving the N atom from the DMB group (at 2.071 Å), which clearly indicated the presence of a N–Co coordination bond and a noncovalent contact involving a HIS residue (HIS173), positioned at the end of a flexible loop that preceded one of the α-helices at 2.800 Å from the Co center. Both the N···Co distance and the N···Co–N_DMB_ angle (164.6°) were consistent with the formation of a N_HIS_···Co σ-hole complex. HIS173 is a crucial amino acid involved in Cbl–TC binding, a fact highlighted by the original authors,^47^ who concluded that the length and mobility of this HIS loop provided lability to the HIS···Co interaction, which was considered a semicoordinated bond (further assessed by the observation of higher rate constants regarding HIS replacement in human TC compared to the bovine derivative). The computed interaction energy value of the N_HIS_···Co σ-hole interaction resulted in −12.4 kcal·mol^–1^, which corresponds to a moderately strong value far from the typical energy of a N–Co coordination bond (usually between 30 and 50 kcal·mol^–1^). This example illustrates the importance of σ-hole bonds in Cbl biochemistry, specifically for guiding Cbl–TC molecular recognition. We propose that the weak nature of the N···Co interaction might be crucial for the release of Cbl in the cellular cytoplasm, as this is the last step carried out by the TC protein, thus being key for the regular functioning of this enzyme.

In the second example (see Figure 5b),^48^ the X-ray crystal structure of cobalamin adenosyltransferase (ATR) was obtained at 1.87 Å resolution. This enzyme is responsible for (i) the synthesis of 5-deoxyadenosylcobalamin (AdoCbl) in a reductive adenosylation reaction and (ii) the delivery of AdoCbl to methylmalonyl-CoA mutase (MCM). In their study, the authors investigated the mechanism by which ATR signals whether its cofactor cargo is ready (AdoCbl) or not (Cbl) for transfer to MCM. The X-ray structures they obtained revealed a Cbl system in its “base-off” conformation (exhibiting a square planar coordination environment), since the DMB is not coordinated to Co and is buried inside the enzyme’s pocket instead. The authors found a protein loop containing a PHE residue (PHE117) located on the other side of the corrin ring (at 3.584 Å), which precluded access of water molecules to the active site and enforced the “base-off” state. This PHE···Co interaction could be interpreted as a C=C···Co π-hole complex involving the π-system of PHE. The computed interaction energy strength resulted in −14.7 kcal·mol^–1^, consistent with the value of a noncovalent interaction, and highlights the role of noncovalent C=C···Co π-hole interactions in the stability of a specific protein conformation. In this regard, we propose that the formation of a Cbl π-hole complex stabilizes the Co­(II) center prior to the second single-electron reduction that converts Co­(II) into Co­(I) within the enzyme’s active site.

These two first examples highlight the importance of Co noncovalent chemistry in protein stabilization and might affect both substrate positioning (by altering the geometry of the enzyme’s active site) as well as barrier modulation (owing to the strength of the NCIs established between HIS/PHE amino acids and Cbl).

The last selected example^49^ encompasses a human methylmalonyl-CoA mutase (human MCM) structure solved at 2.20 Å and exhibiting a square planar Co coordination fashion, which catalyzes the isomerization of methylmalonyl-CoA to succinyl-CoA using AdoCbl as a metallocofactor. During catalysis, the occasional escape of the 5′-deoxyadenosine (dAdo) moiety leaves the Cbl intermediate stranded and prone to hyperoxidation to hydroxocobalamin, which is a difficult process to reverse. In their study, the authors identified that the use of ADP was effective in protecting against Cbl overoxidation. Interestingly, in one of the X-ray structures (8DYJ), the ADP moiety interacts with the Cbl through a noncovalent contact (C···Co distance of 3.602 Å), and we propose that this interaction is crucial for blocking one of the axial coordination positions of Cbl, thus preventing the overoxidation reaction of Cbl. The computed interaction energy resulted in −30.5 kcal·mol^–1^, which accounted for the C–H···Co interaction as well as one O···H hydrogen bond (HB) involving a −H_2_C–OH group from the ADP moiety and a methyl group from Cbl (not shown in Figure 5c). We propose that the formation of a Cbl π-hole complex stabilizes the hydrogen abstraction of the 5′-deoxyadenosyl radical, thus being directly involved in the catalytic mechanism of human MCM. This structure exemplifies the plausible influence of these NCIs in cofactor activation, owing to the involvement of an ADP molecule in the formation of the noncovalent complex.

As the final part of the PDB study and since we found both Cbl “base-on” and “base-off” states as a result of the PDB survey, we carried out several relaxed scans involving Cbl, MeCbl, OHCbl, CNCbl, and ImHCbl and the DMB group to understand the influence of the N_DMB_···Co interaction on Cbl stability (see Figure 6 below). In Figure 6a, the results showed the formation of a σ-hole-based noncovalent local energy minima in those complexes involving CNCbl (colored in green) and ImHCbl (colored in blue) around 3.4 Å, and a π-hole local energy minima (also at around 3.4 Å) in the case of Cbl (colored in yellow), which could potentially act as stable prestates prior to the formation of the N_DMB_–Co coordination bond. In the cases of OHCbl (colored in red) and MeCbl (colored in gray), we did not find a noncovalent minima, indicating that the formation of a coordination bond was more favored in these two Cbl derivatives. In Figure 6b, we represent the N_DMB_···Co distance vs the N···Co–N angle for all five cases. We found that at the intermolecular N···Co distances where the noncovalent minima were found, the N_DMB_···Co–N_corrin_ angle is comprised between 75 and 80°, very close to the 90° value, which would be ideal for the interaction with the σ-/π-hole present in the Co atom.

**Figure 6:**
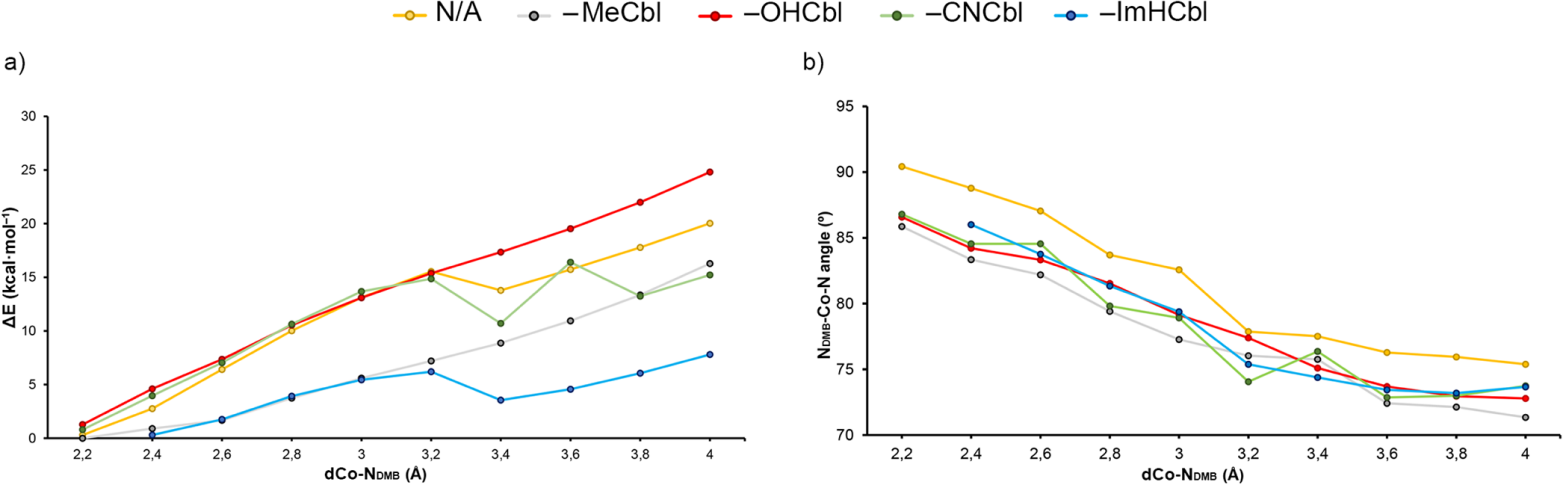
Graphical representation of (a) the relationship between the Co···N_DMB_ distance (dCo-N_DMB_ in Å) and the energetic difference with respect to a Co–N coordinated state (Δ*E* in kcal·mol^–1^) and of (b) the relationship between the Co···N_DMB_ distance (dCo-N_DMB_ in Å) and the N_DMB_-Co-Ncorrin angle (N_DMB_–Co-N in °). N/A stands for the axially uncoordinated Co atom exhibiting a square planar geometry.

### Computational Study on Cbl σ-/π-Hole Donor Ability

To further investigate the formation of a σ-/π-hole noncovalent complex involving Cbl and its derivatives, we conducted a computational study involving several C, N, and O donor Lewis bases (A in Figure 7), such as NH_3_, NCH, OCHNH_2_, and O­(CH_3_)_2_. The results are shown in Figures 7–9 as well as in Tables 1, S2 and S3. More specifically, we carried out a relaxed scan on the A···Co distance (see Computational Methods in the Supporting Information for more details) and found a noncovalent local minimum in most of the cases. Concretely, most A···Co distances ranged from 3 and 3.8 Å, and only in complexes **6** and **17** to **20** we observed A···Co distances between 2.5 and 3.0 Å, which could be attributed to a semicoordinated geometry.

**Figure 7:**
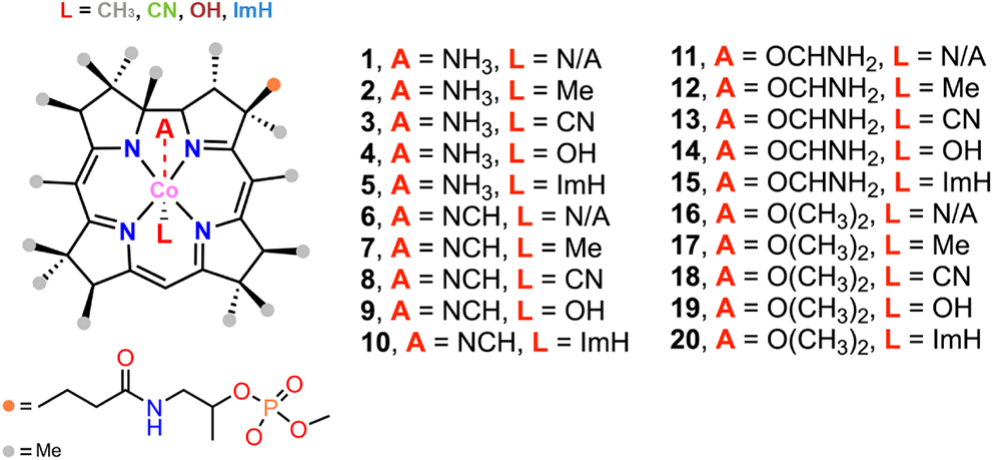
Schematic representation of complexes **1** to **20** studied herein. N/A indicates an axially uncoordinated Co center (square planar geometry).

**Figure 8:**
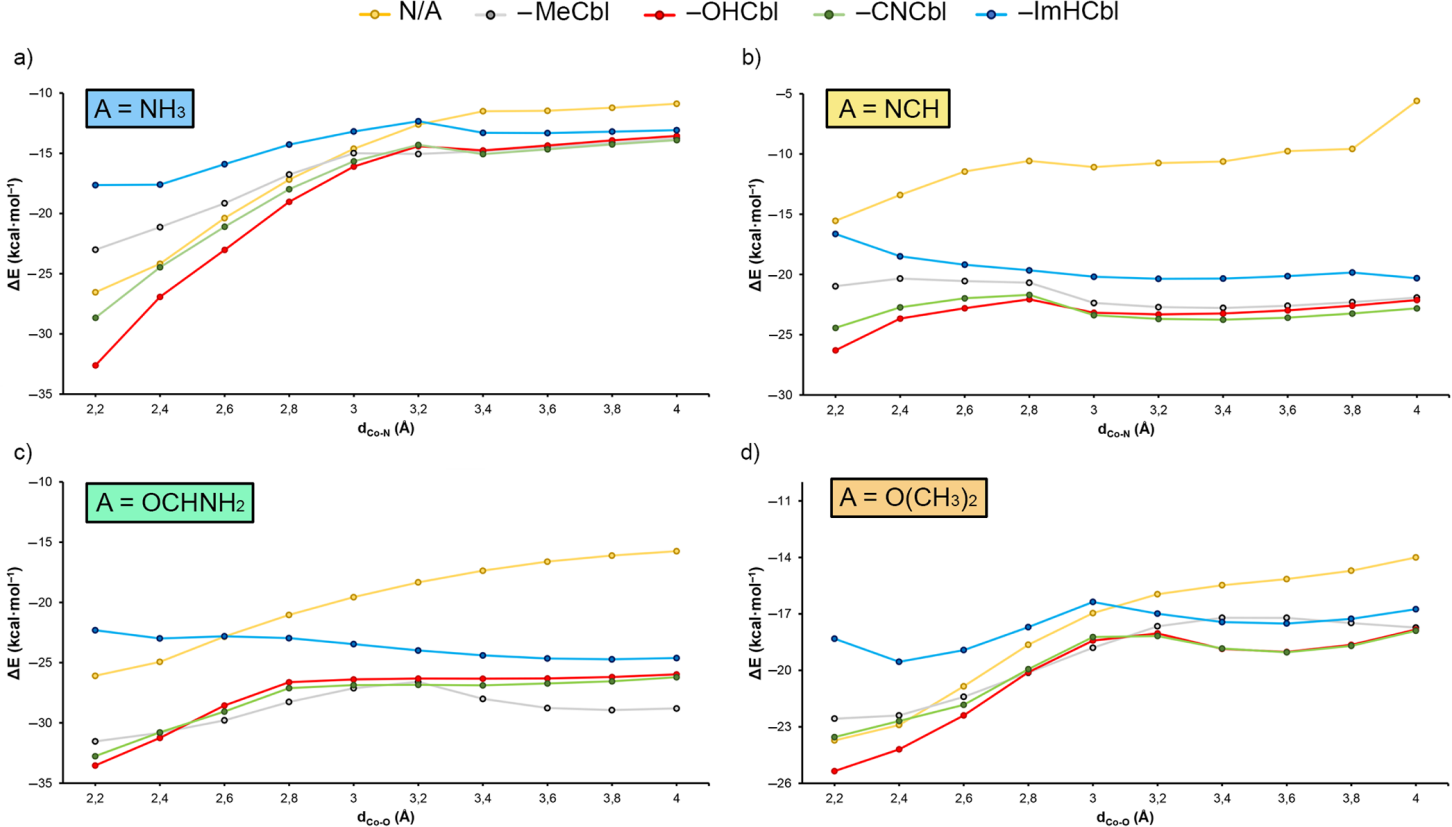
Graphical representations of the A···Co (A = NH_3_, NCH, OCHNH_2_, and O­(CH_3_)_2_) relaxed distance scans were generated using a square planar (N/A) and a square pyramidal corrin system (with −CH_3_, −OH, −CN, and −ImH group as axial ligands). The plot represents the Co···A distance (d_Co–O_ and d_Co–N_, in Å) vs the energetic difference between each point of the scan and a fully relaxed corrin ring complex (Δ*E* in kcal·mol^–1^).

**Table 1:** Interaction Energies (Δ*E*, in kcal·mol^–1^), Intermolecular A···Co Distances (D, in Å), Average Values of the A···Co–N Angle (α_A···Co–N_, in °) and A···Co–L Angle (α_A···Co–L_, in °); Values of the Electron Density (ρ), Its Laplacian (∇^2^ρ), Potential (V), and Kinetic (*G*) Energies in au; and the −*G*/*V* Ratio for Complexes 2 to 10, 12 to 15, and 17 to 20 at the BP86-D3/def2-TZVP Level of Theory^a^

Complex^b^ ^b^	Δ*E* ^a^ ^a^	d^c^ ^c^	α_A···Co–N_	α_A···Co–L_	ρ^d^ ^d^	∇^2^ρ^d^ ^d^	*V* ^d^ ^d^	*G* ^d^ ^d^	–*G*/*V*
**2** H_3_N···Co–CH_3_	–10.6 (−8.6) [−8.8]	3.140	88.0	162.7	0.99	2.48	–0.58	0.60	1.03
**3** H_3_N···Co–CN	–11.2 (−10.9) [−11.3]	3.260	87.4	164.8	0.84	2.19	–0.42	0.49	1.16
**4** H_3_N···Co–OH	–11.0 (−9.6) [−9.7]	3.220	87.6	164.8	0.88	2.04	–0.47	0.49	1.04
**5** H_3_N···Co–ImH	–9.2 (−7.8) [−7.9]	3.500	87.2	158.3	0.48	1.55	–0.28	0.27	0.96
**6** HCN···Co	–9.0 (−6.5) [−5.9]	2.718	91.1		1.95	4.42	–1.35	1.23	0.91
**7** HCN···Co–CH_3_	–18.9 (−16.3) [−16.7]	3.237	87.4	166.0	0.79	1.97	–0.44	0.47	1.06
**8** HCN···Co–CN	–19.9 (−17.5) [−17.9]	3.253	86.6	167.5	0.78	1.88	–0.40	0.44	1.10
**9** HCN···Co–OH	–19.1 (−16.4) [−16.6]	3.141	86.9	171.3	0.93	2.25	–0.54	0.55	1.02
**10** HCN···Co–ImH	–17.3 (−16.0) [−16.2]	3.300	88.0	157.7	0.78	2.37	–0.41	0.50	1.22
**12** NH_2_HCO···Co–CH_3_	–22.0 (−18.0) [−18.4]	3.780	87.2	166.2	2.28	5.63	–1.69	1.55	0.92
**13** NH_2_HCO···Co–CN	–21.1 (−20.0) [−19.1]	3.400	86.6	153.7	0.41	1.68	–0.29	0.31	1.07
**14** NH_2_HCO···Co–OH	–20.5 (−19.3) [−18.4]	3.420	86.8	154.6	0.43	1.38	–0.24	0.28	1.16
**15** NH_2_HCO···Co–ImH	–19.0 (−17.5) [−16.4]	3.780	87.0	146.8	0.28	1.07	–0.17	0.19	1.12
**17** (CH_3_)_2_O···Co–CH_3_	–13.1 (−8.9) [−10.5]	2.587	86.9	168.0	1.15	2.33	–0.65	0.61	0.94
**18** (CH_3_)_2_O···Co–CN	–14.3 (−12.4) [−13.2]	2.905	86.0	160.4	0.76	2.11	–0.41	0.47	1.15
**19** (CH_3_)_2_O···Co–OH	–14.1 (−12.1) [−12.8]	2.849	87.5	149.1	0.37	1.30	–0.20	0.26	1.30
**20** (CH_3_)_2_O···Co–ImH	–12.9 (−9.8) [−11.1]	2.789	87.7	150.5	0.89	2.29	–0.51	0.54	1.06

a Values in parentheses and brackets correspond to the PBE0-D3/def2-TZVP and ωb97X-D/def2-TZVP levels of theory, respectively.

b Complexes **1**, **11**, and **16** were not included since they did not represent a noncovalent minimum.

c Values are given as the shortest distance between the Lewis base and the Co atom.

d Values are multiplied by 100.

**Figure 9:**
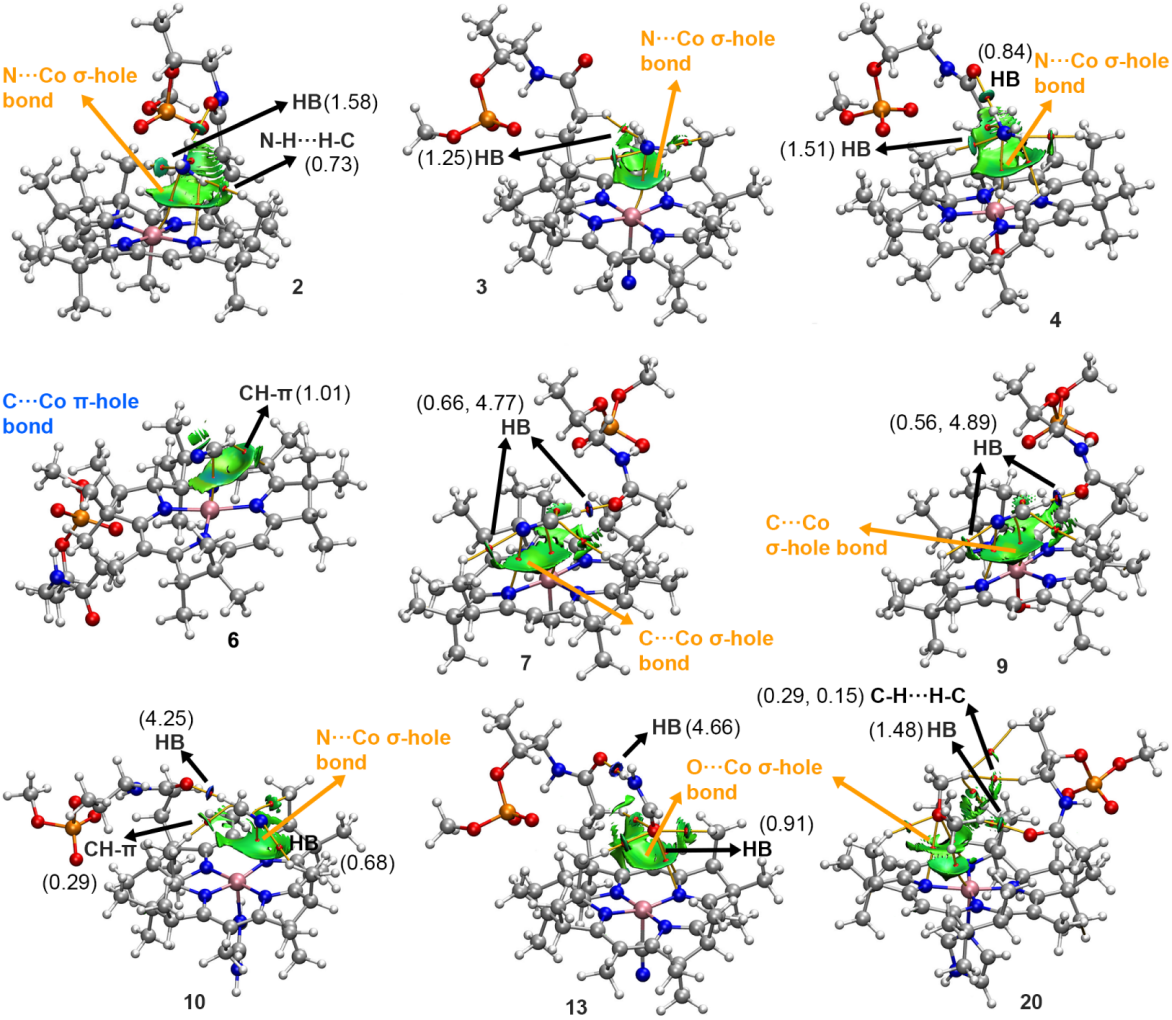
NCIplot analysis and QTAIM distribution of intermolecular bond critical points (bcps in red spheres) and bond paths in complexes **2** to **4**, **6**, **7**, **9**, **10**, **13**, and **20**. The names of the NCIs involved in the A···Cbl recognition (A = NH_3_ in **2**, **3**, and **4**, NCH in **6**, **7**, **9**, and **10**, OCHNH_2_ in **13** and O­(CH_3_)_2_ in **20**) are also indicated, including the values of the density at the bcps that characterize these interactions, expressed in au. NCIplot surfaces include only intermolecular contacts between A and the corrin ring. NCIplot color range −0.04 au ≤ (signλ_2_)­ρ ≤ + 0.04 au. Isosurface value: RDG = 0.5 and ρ cutoff = 0.05 au.

As noted in Figure 8, the NH_3_ and O­(CH_3_)_2_ involving scans (Figure 8a,d) exhibited a similar profile (resembling the shape of a shoulder) where the Δ*E* values (in kcal·mol^–1^), which correspond to the energetic difference between the fully optimized coordination complex and each point of the scan, rose abruptly from 2.2 to 3.0–3.2 Å. Once this point was reached, the noncovalent local minima were found, followed by a subtle increase in the Δ*E* values up to 4.0 Å. On the other hand, in the case of NCH and OCHNH_2_ involving scans (Figure 8b,c) a similar behavior between them was also observed, consisting of initially less pronounced curve and obtaining noncovalent minima around 3.4 Å. The steeper Δ*E*/*d* profiles observed for NH_3_ and O­(CH_3_)_2_ molecules compared to NCH and OCHNH_2_ profiles are due to more pronounced spatial rearrangements of the electron donor molecules over the corrin’s ring plane along the course of the relaxing scan process.

Once the initial A···Co scanned distance was refined (see Supporting Information for more details), in complexes **1** to **5** involving NH_3_ (Figure 8a) noncovalent minima were found for all four Cbl derivatives (at 3.140 Å for MeCbl (**2**), 3.260 Å for CNCbl (**3**), 3.220 Å for OHCbl (**4**), and 3.500 Å for ImHCbl (**5**)). The interaction energies obtained were −10.6 kcal·mol^–1^ (**2**), −11.2 kcal·mol^–1^ (**3**), −11.0 kcal·mol^–1^ (**4**) and −9.2 kcal·mol^–1^ (**5**), all of them being attractive and moderately strong in nature. For complexes **6** to **10** involving NCH (Figure 8b), noncovalent minima were also found for all four Cbl derivatives as well as for the axially uncoordinated Co center (at 2.718 Å for N/A (**6**), 3.237 Å for MeCbl (**7**), 3.253 Å for CNCbl (**8**), 3.141 Å for OHCbl (**9**), and 3.300 Å for ImHCbl (**10**)). In this set of complexes, the interaction energy values obtained were −9.0 kcal·mol^–1^ (**6**), −18.9 kcal·mol^–1^ (**7**), −19.9 kcal·mol^–1^ (**8**), −19.1 kcal·mol^–1^ (**9**), and −17.3 kcal·mol^–1^ (**10**), indicating stronger magnitude than those observed in complexes involving NH_3_ (see the section QTAIM and NCIplot Analyses below).

For complexes **11** to **15** involving OCHNH_2_ (Figure 8c) the noncovalent minima were found at 3.780 Å for MeCbl (**12**), with an interaction energy value of −22.0 kcal·mol^–1^ at 3.400 Å for CNCbl (**13**), exhibiting an interaction energy value of −21.1 kcal·mol^–1^ at 3.420 Å for OHCbl (**14**), with a strength of −20.5 kcal·mol^–1^, and lastly, at 3.780 Å for ImHCbl (**15**), showing an interaction strength of −19.0 kcal·mol^–1^. Finally, among complexes **16** to **20** involving O­(CH_3_)_2_ (Figure 8d), the noncovalent minima were found at 4.100 Å for MeCbl (**17**), obtaining an interaction energy strength of −13.1 kcal·mol^–1^ at 2.905 Å for CNCbl (**18**), with an interaction energy strength of −14.3 kcal·mol^–1^ at 2.849 Å for OHCbl (**19**) and at 2.789 Å for ImHCbl (**20**), with interaction energy values of −14.1 and −12.9 kcal·mol^–1^, respectively. It is also important to note that these A···Co distances correspond to the shortest values between the Lewis base and the Co center.

In general, the tendency observed when comparing the energetic values involving the same Lewis base agrees well with the Co σ-hole MEP values discussed above (Figure 2), thus indicating that this noncovalent bond plays an important role in the stabilization of these noncovalent minima. On the other hand, in complexes **1**, **11**, and **16** involving the square planar Co center, we did not find an energy minimum based on a noncovalent interaction, as we observed a constant increase in the Δ*E* values; therefore, no energetic data regarding these complexes were provided in Table 1. Furthermore, comparing these complexes while keeping constant the Cbl derivative also agrees with the Lewis basicity character exhibited by the electron donor molecules. Only in the case of complexes **11** to **15** involving OCHNH_2_, this behavior is not observed, due to the presence of strong NH···O HBs that also contribute to the stabilization of the noncovalent complexes. More in detail, aside from the A···Co interaction, ancillary HB, C–H···H–C, N–H···H–C, and CH−π interactions are also present in these systems, which further contribute to the formation of these noncovalent minima (see the section QTAIM and NCIplot Analyses below). We have also computed the interaction energies of all complexes studied using the PBE0 and ωB97X-D methods, obtaining values similar to those retrieved using the BP86 functional, thus providing reliability to the results obtained using the latter.

Regarding the geometrical disposition of the Lewis base over the Cbl system, we found that in most cases, the average value of the A···Co–N angle is close to 90° (ranging between 86.0° and 91.1°), which is in line with a perpendicular arrangement of the electron-rich atom over the Cbl molecular plane. In addition, we have also measured the A···Co–L angle, which ranges from 146.8 to 171.3°, denoting a preferential directionality marked by the presence of the Co σ-hole.

As a final remark, we also obtained the wave function parameters involving the Lewis base···Co interaction for the complexes described above, such as ρ, ∇^2^ρ, *V*, *G*, and the −*G*/*V* ratio, which are included in Table 1. Interestingly, in most of the cases, the −*G*/*V* ratio obtained is close to 1, thus further confirming the weak nature of the complexes listed in Table 1.

### QTAIM and NCIplot Analyses

In Figure 9, the combined QTAIM and NCIplot analyses involving several representative complexes (**2** to **4**, **6**, **7**, **9**, **10**, **13**, and **20**) are shown regarding intermolecular A···Cbl recognition (see Supporting Information for the results involving the Natural Bonding Orbital (NBO) study). Interestingly, a C/N/O···Co bond critical point (bcp) and a bond path were found in complexes **6** and **7** (involving the C atom from NCH), **2** to **4** (involving the N atom from ammonia), **9** (involving the C–N triple bond from NCH), and **20** (involving the O atom from O­(CH_3_)_2_), which characterized the presence of the noncovalent A···Co σ-/π-hole bond (highlighted in orange and blue in Figure 9, respectively). Additionally, ancillary HB, N–H···H–C, C–H···H–C, and CH−π interactions were also present in the supramolecular complexes studied.

For instance, in complex **2**, we observed two main ancillary interactions involving (i) the ammonia N lone pair, which interacts with a C–H bond from one of the corrin’s methyl groups, characterized as a HB and (ii) the N–H bond from ammonia and the C–H bond from another methyl group in the corrin system, thus denoting the presence of a N–H···H–C interaction. On the other hand, in complex **3**, a bcp and a bond path characterized an ancillary HB between the ammonia N atom and a C–H bond from a corrin methyl group. In complex **4**, two ancillary HBs were observed, involving the N atom and a N–H bond from ammonia as either HB acceptor or donor, respectively, interacting with (i) a C–H bond from a vicinal methyl group and (ii) the sp^2^ O from the corrin’s propionamide group. In complex **6**, an ancillary CH−π interaction is characterized by a bcp and bond path connecting the C–H bond of NCH to an sp^2^ C atom from the corrin ring.

In complexes **7**, **9**, and **10**, two HBs assist in the stabilization of the NCH molecule, in addition to the C···Co interaction, as denoted by the two bcps and bond paths connecting (i) the C–H bond of NCH and the sp^2^ O atom from the corrin amide group and (ii) the N lone pair of NCH and a C–H bond from a neighboring group. In complex **13**, several HBs are established between the sp^2^ O from the OCHNH_2_ moiety and vicinal C–H bonds from the corrin system, while a strong HB between the OCHNH_2_ N–H group and the sp^2^ O atom from the propionamide group is also present in the assembly. Lastly, in complex **20**, ancillary HB and C–H···H–C interactions are denoted by the bcps and bond paths connecting C–H groups from O­(CH_3_)_2_ to (i) the corrin’s amide group and (ii) neighboring corrin methyl groups.

The results from these analyses agree with those derived from the NCIplot study, which revealed a greenish isosurface in all of the cases located between the electron donor molecule and the Co corrin system, serving as an additional descriptor of the NCIs present in these supramolecular complexes. Besides, we found bluish isosurfaces that characterized strong N–H···O and C–H···O HBs, such as those present in complexes **7**, **9**, **10**, and **13**, which agree with the values of the density at their respective bcps.

Overall, the presence of these NCI regions provided valuable graphical information regarding the extent of the A···Co interaction in real space, as well as a way to account for the different noncovalent contributors responsible for the formation of the supramolecular complexes studied herein.

## Conclusions

In the present study, a combined PDB inspection and computational approach was conducted to find evidence of noncovalent L···Co interactions (L = C, N, and O) beyond the classical coordination chemistry of Cbl. In 67 structures out of 111 total found, the Co atom was involved in an NCI with either a protein residue (e.g., PHE, HIS, THR), a water molecule, or an artificial ligand. As a result of our search, we also found structures involving the main Cbl derivatives, MeCbl, CNCbl, and OHCbl, as well as some cases where Cbl was in a “base-off” conformation and the DMB group was replaced by a HIS residue. This led to the formation of a σ-hole over the Co atom, which served as a stability source of semicoordinated/noncovalent C/N/O···Co–L interactions based on the formation of σ-hole complexes. From the PDB search, 10 selected structures were computationally analyzed at the BP86-D3/def2-TZVP level of theory, and three examples were discussed in detail, which demonstrated the involvement of Co σ-hole interactions in (i) protein–Cbl binding, such as in human transcobalamin, (ii) stabilization of specific protein states, such as in cobalamin adenosyltransferase, and (iii) stabilizing substrate mimic molecules, such as in human methylmalonyl-CoA mutase. Aside from this, we investigated the N_DMB_···Co interaction in both Cbl and its derivatives, obtaining a local energy minimum in most of the cases based on either a σ- or a π-hole bond, which shed light on B_12_ complex structural features. On the other hand, by means of theoretical calculations at the BP86-D3/def2-TZVP level of theory, we evidenced the formation of noncovalent A···Co σ-hole and π-hole complexes (A = NCH, NH_3_, OCHNH_2_, and O­(CH_3_)_2_) using Cbl, MeCbl, CNCbl, and OHCbl and ImHCbl derivatives. The physical nature of these supramolecular complexes was analyzed by means of quantum chemistry tools, which shed light into the importance of electrostatics and orbital effects as key contributors to their overall stability. Since this novel noncovalent chemistry is often overlooked in favor of traditional coordination chemistry, we believe that the results reported herein will be significant for scientists working in supramolecular chemistry, bioinorganic chemistry, and biotechnology by describing for the first time Co-involving NCIs within a biological framework, which might be important to fully understand the catalytic mechanisms and substrate/amino acid interacting poses of vitamin B_12_-dependent enzymes.

## Supplementary Material



## Notes

Includes PDB survey details; Computational methods; Figure S1; Table S1; NBO study; A brief comparison with standard Pnictogen and Halogen bond donor complexes; Co NCIs in the Cambridge Structural Database; Cartesian coordinates of the PDB models; Cartesian coordinates of complexes **2** to **10**, **12** to **15**, and **17** to **20**; Cartesian coordinates of the DMB···Co relaxed scans; Cartesian coordinates of complexes **21** to **52**; Cartesian coordinates of TUDDEE structure model (PDF)

## Abbreviation

Cbl: Cobalamin
NCI: Noncovalent interaction
DMB: 5,6-dimethylbenzimidazole
SAM: *S*-adenosylmethionine
CNCbl: cyanocobalamin
OHCbl: hydroxocobalamin
MeCbl: methylcobalamin
ppy: porphyrin
HalB: halogen bonding
PDB: Protein Data Bank
ImHCbl: imidazole-coordinated Cbl
MEP: molecular electrostatic potential
HIS: histidine
PHE: phenylalanine
THR: threonine
SER: serine
TC: transcobalamin
ATR: adenosyltransferase
AdoCbl: 5-deoxyadenosylcobalamin
MCM: methylmalonyl-CoA mutase
dAdo: 5′-deoxyadenosine
QTAIM: Quantum Theory of Atoms in Molecules
NCIplot: noncovalent interaction plot
NBO: natural bonding orbital
