# Supplementary material for: Is Cobalt Joining the σ‑/π-Hole Crown? Exploring Noncovalent Interactions in Cobalamin
Source: JACS Au. 2025 Oct 9;5(11):5438–49. doi: 10.1021/jacsau.5c00955 (PMC12648342; doi:10.1021/jacsau.5c00955)
Supplement: Supplementary file 1 [file au5c00955_si_001.pdf]

Supporting Information

Is Cobalt Joining the σ-/π-Hole Crown? Exploring Noncovalent Interactions in Cobalamin

Sergi Burguera[a] and Antonio Bauzá*[a]

[a]Departament de Química, Universitat de les Illes Balears, Ctra. de Valldemossa km 7.5, 07122 Palma de Mallorca (Baleares), Spain

E-mail: antonio.bauza@uib.es

PDB survey details
page 2

Computational methods
page 2

Figure S1
page 5

Table S1
page 5

NBO study
page 6

A brief comparison with standard Pnictogen and Halogen bond donor complexes
page 10

Co NCIs in the Cambridge Structural Database
page 14

Cartesian coordinates of the PDB models
page 15

Cartesian coordinates of complexes 2 to 10, 12 to 15 and 17 to 20
page 53

Cartesian coordinates of the DMB···Co relaxed scan
page 108

Cartesian coordinates of complexes 21 to 52
page 120

Cartesian coordinates of TUDDEE structure model
page 130

PDB survey details

The PDB was inspected to statistically evaluate the tendency of cobalamin to form coordination/noncovalent bonds. To achieve this, the following criteria were used:

- Only X-ray structures were considered.
- No specific X-ray structure resolution criteria were imposed, except that those structures beyond 4Å resolution were discarded. Most of the structures were solved at ~2.0–3.0Å resolution, providing some uncertainty to the crystallographic coordinates when attempting to make sub-angstrom distinctions.
- In our analysis, the two distances corresponding to the two axial positions of the corrin system were inspected for each structure, leading to the data shown in Figures 3, 4 and S1.
- The distance cutoffs used to classify a contact as a coordination bond, or a noncovalent contact were:
    - Distances below 2.5Å were considered as coordination bonds (the sums of the Co+C/N/O covalent radii are 2.0, 1.97 and 1.88Å, respectively).[50] The total number of coordination bonds found was 252.
    - Distances comprised between 2.5Å and the sum of the Co+C/N/O van der Waals radii +0.5Å (4.67Å for C, 4.56Å for N and 4.40Å for O) were considered as noncovalent contacts. The total number of noncovalent contacts found was 157, corresponding to 67 structures.
    - All the structures found were manually inspected prior to the analysis of the L···Co distances and L···Co–N angles.

Computational methods

General information

The interaction energies of all complexes included in this study were computed at the BP86[51,52]-D3[53]/def2-TZVP[54] level of theory. The calculations have been performed using the program TURBOMOLE version 7.7.[55] The interaction

[50] Cordero, B.; Gómez, V.; Platero-Prats, A. E.; Revés, M.; Echeverría, J.; Cremades, E.; Barragán F.; Álvarez S. Covalent radii revised. *Dalton Trans.*, **2008**, *31 (21)*, 2832-2838.

[51] Becke, A.D. Density-functional exchange-energy approximation with correct asymptotic behavior. *Phys. Rev. A*, **1988**, *38 (6)*, 3098-3100.

[52] Perdew, J. P. Density-functional approximation for the correlation energy of the inhomogeneous electron gas. *Phys. Rev. B*, **1986**, *33 (12)*, 8822-8824.

[53] Grimme, S.; Antony, J.; Ehrlich S.; Krieg H. A consistent and accurate *ab initio* parametrization of density functional dispersion correction (DFT-D) for the 94 elements H-Pu. *J. Chem. Phys.*, **2010**, *132 (15)*, 154104.

[54] Schäfer, A.; Horn, H.; Ahlrichs, R. Fully optimized contracted Gaussian basis sets for atoms Li to Kr. *J. Chem. Phys.*, **1992**, *97 (4)*, 2571-2577.

[55] Balasubramani, S. G.; Chen, G. P.; Coriani, S.; Diedenhofen, M.; Frank, M. S.; Franzke, Y. J.; Furche, F.; Grotjahn, R.; Harding, M. E.; Hättig, C.; Hellweg, A.; Helmich-Paris, B.; Holzer, C.; Huniar, U.; Kaupp, M.; Marefat Khah, A.; Karbalaei Khani, S.; Müller, T.; Mack, F.; Nguyen, B. D.; Parker, S. M.; Perlt, E.; Rappoport, D.; Reiter, K.; Roy, S.; Rückert, M.; Schmitz, G.; Sierka, M.; Tapavicza, E.; Tew, D. P.; van Wüllen, C.; Voora, V. K.; Weigend, F.; Wodyński, A.; Yu, J. M. TURBOMOLE: Modular Program Suite for Ab Initio Quantum-Chemical and Condensed-Matter Simulations. *J. Chem. Phys.*, **2020**, *152* (*18*), 184107.

energies were calculated using the supermolecule approximation ($\Delta E = E_{complex} - E_{monomer1} - E_{monomer2}$). The MEP surfaces were also computed at the BP86-D3/def2-TZVP level of theory by means of the Gaussian 16 software[56] and analyzed using the Gaussview 5.0 program.[57] In these systems, the Cbl moiety was modeled by replacing the hanging propionamide groups by methyl moieties while the chain linking the DMB group and the corrin ring was replaced by a methyl phosphate group.

The calculations for the wavefunction analysis were carried out at the BP86-D3/def2-TZVP level of theory using the Multiwfn software.[58] The NBO[59] analyses were performed at the BP86-D3/def2-TZVP level of theory. Lastly, the NCIplot[60] isosurfaces correspond to both favorable and unfavorable interactions, as differentiated by the sign of the second-density Hessian eigenvalue and defined by the isosurface color. The color scheme is a red−yellow−green−blue scale, with red for repulsive (ρ_{cut}^{+}) and blue for attractive (ρ_{cut}^{-}) NCI interaction density. Yellow and green surfaces correspond to weak repulsive and weak attractive interactions, respectively. The surfaces were visualized using the Visual Molecular Dynamics (VMD) software.[61]

Details regarding calculations involving PDB models

Initially, the 10 selected structures were downloaded from the PDB webpage and inspected using the Biovia Discovery Studio visualizer program.[62] These were chosen to give a general overview of different scenarios involving Cbl, such as different protein families, different interacting moieties as well as different Co coordination environments. More in detail, the Cbl moiety was modeled by replacing the hanging propionamide groups by methyl moieties except in the case of the chain connecting the corrin ring with the DMB moiety, which was kept as

---

[56] Gaussian 16, Revision C.01, Frisch, M. J.; Trucks, G. W.; Schlegel, H. B.; Scuseria, G. E.; Robb, M. A.; Cheeseman, J. R.; Scalmani, G.; Barone, V.; Petersson, G. A.; Nakatsuji, H.; Li, X.; Caricato, M.; Marenich, A. V.; Bloino, J.; Janesko, B. G.; Gomperts, R.; Mennucci, B.; Hratchian, H. P.; Ortiz, J. V.; Izmaylov, A. F.; Sonnenberg, J. L.; Williams-Young, D.; Ding, F.; Lipparini, F.; Egidi, F.; Goings, J.; Peng, B.; Petrone, A.; Henderson, T.; Ranasinghe, D.; Zakrzewski, V. G.; Gao, J.; Rega, N.; Zheng, G.; Liang, W.; Hada, M.; Ehara, M.; Toyota, K.; Fukuda, R.; Hasegawa, J.; Ishida, M.; Nakajima, T.; Honda, Y.; Kitao, O.; Nakai, H.; Vreven, T.; Throssell, K.; Montgomery, J. A., Jr.; Peralta, J. E.; Ogliaro, F.; Bearpark, M. J.; Heyd, J. J.; Brothers, E. N.; Kudin, K. N.; Staroverov, V. N.; Keith, T. A.; Kobayashi, R.; Normand, J.; Raghavachari, K.; Rendell, A. P.; Burant, J. C.; Iyengar, S. S.; Tomasi, J.; Cossi, M.; Millam, J. M.; Klene, M.; Adamo, C.; Cammi, R.; Ochterski, J. W.; Martin, R. L.; Morokuma, K.; Farkas, O.; Foresman, J. B.; Fox, D. J. Gaussian, Inc., Wallingford CT, 2016.

[57] Dennington, R.; Keith T. A.; Millam, J. M. GaussView (Version 5), Semichem Inc., Shawnee Mission, KS, USA, 2016.

[58] Lu, T.; Chen, F. Multiwfn: A multifunctional wavefunction analyzer, *J. Comp. Chem.*, **2011**, *33 (5)*, 580-592.

[59] Glendening, E. D.; Badenhoop, J. K.; Reed, A. E.; Carpenter, J. E.; Bohmann, J. A.; Morales, C. M.; Karafiloglou, P.; Landis, C. R.; Weinhold, F. NBO 7.0, Theoretical Chemistry Institute; University of Wisconsin, Madison, 2018.

[60] Contreras-García, J.; Johnson, E. R.; Keinan, S.; Chaudret, R.; Piquemal, J. -P.; Beratan, D. N.; Yang, W. NCIPLOT: A program for plotting Noncovalent Interaction Regions. *J. Chem. Theory Comput.*, **2011**, *7 (3)*, 625-632.

[61] Humphrey, W.; Dalke, A.; and Schulten K. VMD: Visual molecular dynamics. *J. Molec. Graphics,* **1996**, *14 (1)*, 33-38.

[62] BIOVIA, Dassault Systèmes, Discovery Studio visualizer (19), San Diego, 2019.

is. Additionally, the two axial ligands were included in the theoretical models. These were:

- Protein residues (HIS in 2BB5, PHE in 6D5K, 6WH5, 7RUV and 7RUU and THR in 4DJD structures) interacting with the Co center were capped at both sides by methyl groups.
- Substrate molecules (5'-deoxyadenosine in structures 2XIQ, 6OXC, 6WGV and 8DYJ) were left unchanged.

Once the modeling phase was concluded, the H atoms of the supramolecular complexes were relaxed at the BP86-D3/def2-SVP level of theory and the interaction energies calculated using the def2-TZVP basis set by means of single point calculations (see the cartesian coordinates of the PDB models used below).

Details regarding relaxed N_{DMB}···Co scans (results shown in Figure 6)

We carried out relaxed scan calculations at the BP86-D3/def2-SVP level of theory using the DMB moiety and i) the square planar Co corrin ring and ii) the Cbl derivatives (MeCbl, CNCbl, OHCbl and ImHCbl). In these systems, the Cbl moiety was modeled by replacing the hanging propionamide groups by methyl moieties while the chain linking the DMB group and the corrin ring was replaced by a methyl phosphate group.

Initially, the whole system was freely optimized, leading to the formation of either a square pyramidal or octahedral coordination complex. From here, the N_{DMB}···Co distance was increased using 0.2Å increments from the N–Co coordination bond distance up to 4.0Å. For example, in the OHCbl derivative a N_{DMB}···Co distance of 2.04 Å was obtained from its equilibrium geometry. Thus, relaxed scans were performed using an interval of 0.2 Å and optimizing the geometry (keeping the N···Co distance frozen) at 2.2Å, 2.4Å, 2.6Å and so on. This led to the graphical representations shown in Figure 6 where we represented the N_{DMB}···Co scanned distance ($d_{Co–NDMB}$ in Å, x axis) *vs* the energetic difference between the optimized coordination complex and each point of the scan (ΔE in kcal·mol^{-1}, y axis). Once a noncovalent minimum was identified, the DMB···Co interaction energy was computed at the BP86-D3/def2-TZVP level of theory (using the supermolecule approximation shown above). Frequency calculations were not performed on the structures found, since the objective was to find noncovalent local minima and not global minima, which would probably result in the formation of a Co coordination complex.

Details regarding relaxed A···Co scans in complexes 1 to 20 (results shown in Figure 8)

In this case, we used the same Cbl systems (modeled in the same way as the previously mentioned scans involving the DMB group) and several N, C and O Lewis bases (NCH, NH_3, $OCHNH_2$ and $O(CH_3)_2$) and performed the same relaxed scan strategy. However, for these complexes if an energy minimum was located, we performed a "refined" scan starting from that A···Co distance. The refined scan was carried out at steps of 0.02Å between the energy minima and the previous/next point from the original scan. For instance, if a local minimum was

located at 3.2Å (e.g. HCN···OHCbl complex) in the initial relaxed scan, a "refined" scan was performed starting from the geometry obtained at 3.2Å and increasing or decreasing the distance by 0.02Å until arriving to 3.0Å and 3.4Å, respectively, which were the previous and following points from the relaxed scan. Lastly and following our previous relaxed scan methodology, once the noncovalent minimum was refined, the A···Co interaction energy was computed at the BP86-D3/def2-TZVP level of theory. Again, no frequency calculations were performed as the objective was to identify, quantify and categorize the possible noncovalent interactions regarding Co.

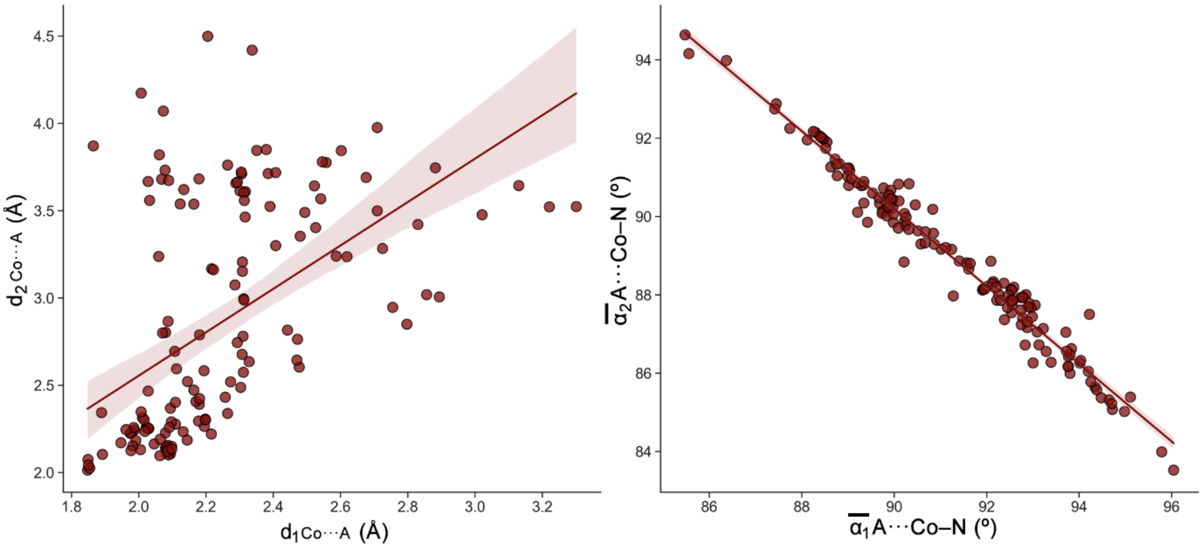

Figure S1. Graphical representations of the linear relationship between the two Co···A distances (d_1 and d_2) as well as between the two A···Co–N angles (α_1 and α_2).

These two representations, as well as those shown in Figure 4 of the main text include a shadowed region which belongs to the confidence interval, thus, the thinner the region is, the more confident the regression line is. The maximum confidence interval value was set to 95%.

Table S1. List of protein families, PDB IDs, resolution (R, in Å), multiplicity of the Co center, interaction energies (ΔE, in kcal·mol^{-1}) and intermolecular L···Co (L = C, N and O) distances (d, in Å) involving the selected PDB structures at the BP86-D3/def2-TZVP level of theory.

Protein family	PDB ID	R	Multiplicity	ΔE	d
**Human Transcobalamin**	2BB5	3.20	Doublet	–12.4	2.800

**Human methylmalonyl-CoA mutase**	8DYJ	2.20	Doublet	–30.5	3.602
	2XIQ	1.95	Doublet	–29.1	3.308
**Mycobacterium tuberculosis methylmalonyl-CoA mutase**	6OXC	1.90	Doublet	–12.6	2.520
**ATP cobalamin adenosyltransferase**	6D5K	2.85	Singlet	–12.0	3.679
	6WGV	2.15	Doublet	–11.3	2.881
	7RUV	2.10	Singlet	–31.8	3.326
	7RUU	1.85	Singlet	–11.1	3.681
	6WH5	1.87	Doublet	–14.7	3.584
**B12-dependent methyltransferase**	4DJD	2.38	Doublet	–19.6	3.259

NBO study

In Tables S2 and S3 the results regarding the second order perturbation analysis are shown involving complexes **2** to **10**, **12** to **15** and **17** to **20**. Only the results gathered in Table S1 were discussed, since they involved those complexes discussed in the main text of the article. We have included the rest of the calculated complexes in Table S2 for completeness.

Firstly, in the case of complexes **6**, **7**, **9** and **10** involving NCH we found an orbital contribution involving a π-bonding (BD) C–N orbital to antibonding (BD*) Co–N/Co–C/Co–O orbitals, thus characterizing the N···Co π-/σ-hole interaction from an orbital perspective. These orbital interactions lie between 0.35 and 2.53 kcal·mol^{-1} and are the main orbital contribution in complexes **6**, **7** and **9**. In addition, in complexes **7**, **9** and **10** we found an orbital interaction between a σ-bonding (BD) C–H orbital to antibonding (BD*) Co–C and Co–N orbitals, which

further contributes to the stabilization of such complexes. Lastly, in complexes **6** and **10** we found an orbital interaction between a lone pair (LP) from the N atom of NCH to an antibonding (BD*) Co–N orbital, although of lower magnitude than the orbital contributions involving the π-bonding (BD) C–N orbital in the case of complex **6**.

Secondly, in complexes **2** and **3** involving NH_3, we found an orbital contribution from the LP of the N atom to antibonding (BD*) Co–C and Co–N orbitals, with a magnitude spanning from 2.44 to 7.01 kcal·mol^{-1}, which further characterized the N···Co σ-hole interactions present in these two complexes. This orbital interaction was not found in complex **4**, instead, orbital interactions between a σ-bonding (BD) N–H orbital to antibonding (BD*) Co–O and Co–N orbitals were found. Lastly, in the case of complexes **13** and **20** involving $OCHNH_2$ and $O(CH_3)_2$ molecules, the main orbital contribution encompassed the O lone pairs (LP) and antibonding (BD*) Co–N and Co–C orbitals in the case of complex **13**, thus denoting the presence of an O···Co σ-hole interaction. On the other hand, in complex **20**, we found an orbital contribution that involved a bonding (BD) C-H orbital from the electron donor molecule and an antibonding (BD*) Co–N orbital from the corrin ring system as the main orbital stabilizing source.

Table S2. Donor and acceptor NBOs with indication of the type of interaction and second-order interaction energy $E^{(2)}$ in complexes **2** to **4**, **6**, **7**, **9**, **10**, **13** and **20**. LP, BD and BD* stand for lone pair, bonding orbital and antibonding orbital, respectively. Energy values are in kcal·mol^{-1}.

Complex	Donor	Acceptor	Type	$E^{(2)}$
**2 (H_3N···Co–CH_3)**	LP N	BD* Co–C	n→σ*	4.13
	LP N	BD* Co–N	n→π*	2.44
**3 (H_3N···Co–CN)**	LP N	BD* Co–C	n→σ*	7.01
	LP N	BD* Co–N	n→π*	2.95
**4 (H_3N···Co–OH)**	BD N–H	BD* Co–N	σ→π*	1.79
	BD N–H	BD* Co–O	σ→σ*	3.34
**6 (HCN···Co)**	LP N	BD* Co–N	n→π*	0.55
	BD C–N	BD* Co–N	π→π*	1.10

**7 (HCN···Co–CH_3)**	BD C–N	BD* Co–C	π→σ*	1.73
	BD C–H	BD* Co–C	σ→σ*	0.57
**9 (HCN···Co–OH)**	BD C–N	BD* Co–N	π→π*	0.80
	BD C–N	BD* Co–O	π→σ*	2.53
	BD C–H	BD* Co–N	σ→π*	0.47
**10 (HCN···Co–ImH)**	LP N	BD* Co–C	n→π*	0.63
	BD C–H	BD* Co–N	σ→π*	0.39
	BD C–N	BD* Co–N	π→π*	0.35
**13 (NH_2HCO···Co–CN)**	LP O	BD* Co–C	n→σ*	1.13
	LP O	BD* Co–N	σ→π*	0.41
**20 ($(CH_3)_2$O···Co–ImH)**	LP O	BD* Co–N	n→π*	0.22
	BD C–H	BD* Co–N	σ→π*	0.49

Table S3. Donor and acceptor NBOs with indication of the type of interaction and second-order interaction energy $E^{(2)}$ in complexes **5, 8**, **12**, **14**, **15, 17-19**. LP, BD and BD* stand for lone pair, bonding orbital and antibonding orbital, respectively. Energy values are in kcal·mol^{-1}.

**Complex**	**Donor**	**Acceptor**	**Type**	**$E^{(2)}$**

**5 (H_3N···Co–ImH)**	LP N	BD* Co–N	n→π*	0.71
**8 (HCN···Co–CN)**	BD C–N	BD* Co–C	π→σ*	1.95
	BD C–H	BD* Co–C	σ→σ*	0.71
	BD C–H	BD* Co–N	σ→π*	0.38
**12 (NH_2HCO···Co–CH_3)**	LP O	BD* Co–N	LP→π*	0.25
	BD C–H	BD* Co–C	σ→σ*	6.77
**14 (NH_2HCO···Co–OH)**	LP O	BD* Co–N	n→π*	0.51
	LP O	BD* Co–O	n→σ*	0.75
**15 (NH_2HCO···Co–ImH)**	LP O	3s* Co	n→s*	0.12
**17 ($(CH_3)_2O$···Co–CH_3)**	BD C–H	BD* Co–N	σ→π*	1.18
	BD C–H	BD* Co–C	σ→σ*	1.77
**18 ($(CH_3)_2O$···Co–CN)**	BD C–H	BD* Co–C	σ→σ*	0.73
	BD C–H	BD* Co–N	σ→π*	0.37
**19 ($(CH_3)_2O$···Co–OH)**	BD C–H	BD* Co–N	σ→π*	0.36
	BD C–H	BD Co–O	σ→σ*	0.69

A brief comparison with standard Pnictogen and Halogen bond donor complexes

To provide a quick comparison between the σ-hole donor ability of the different cobalamin derivatives and canonical σ-hole based interactions such as Pnictogen or Halogen bonds, we have computed the MEP surfaces of several –CH_3/–CN substituted Halogen and Pnictogen bond donor molecules (see Figure S2 and Table S4 below). As expected, we found a σ-hole on either the halogen/pnictogen

atom that becomes more electropositive upon going from Br to I and from As to Sb, as well as by including strong electron withdrawing groups, such as –CN. These results are in line with those shown in Figure 2, where the σ-hole present in the Co center became more electropositive when comparing MeCbl to OHCbl or CNCbl.

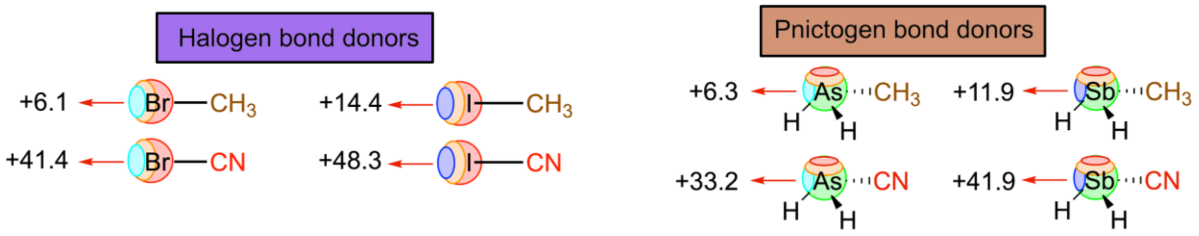

Figure S2. Schematic representation of $–CH_3$ and –CN substituted halogen (Br/I) and pnictogen (As/Sb) bond donor molecules. The values of the electrostatic potential measured at the Br/I/As/Sb σ-hole are also indicated in kcal·mol^{-1} (using a 0.001 a.u. isocontour value).

Table S4. Electrostatic potential values measured at the tip of the Halogen/Pnictogen σ-hole ($V_{σ\text{-hole}}$ in kcal·mol^{-1}). The MEP minima and maxima values for each compound are also indicated in kcal·mol^{-1} (V_{min} and V_{max}, respectively).

**Compound**	**$V_{σ\text{-hole}}$**	**V_{min}**	**V_{max}**
**$Br–CH_3$**	+6.1	–13	+20
**$I–CH_3$**	+14.4	–11	+20
**$As–CH_3$**	+6.3	–15	+15
**$Sb–CH_3$**	+11.9	–7	+16
**Br–CN**	+41.4	–31	+42
**I–CN**	+48.3	–32	+49
**As–CN**	+33.2	–35	+36

Sb–CN	+41.9	–37	+43

In addition, we have computed the interaction energy between the Lewis bases used herein (A = NCH, NH_3, $OCHNH_2$ and $O(CH_3)_2$) and the Halogen/Pnictogen bond donors shown in Figure S2 (see Table S5). In general, the energetic values obtained ranged between –11.4 and –0.3 kcal·mol^{-1}. Although the energies are not strictly comparable from a quantitative point of view (the elements involved are non-metals and there are no ancillary NCIs), we found that the energetic values involving the same Lewis base agreed well with the Halogen/Pnictogen σ-hole MEP values discussed above and that energetic behavior of these complexes when keeping constant the Halogen/Pnictogen bond donor molecule also agrees with the Lewis basicity character exhibited by the electron donor molecules, in line with the results obtained for the Cbl models.

Table S5. Interaction energies (ΔE, in kcal·mol^{-1}), intermolecular A···X (X = Br, I, As, Sb) distances (d, in Å), A···X–R (R = CH_3/CN) angle values ($\alpha_{A\cdots X–R}$ in º) and values of the electron density (ρ), its laplacian ($\nabla^2\rho$), potential (V) and kinetic (G) energies in a.u. and the –G/V ratio at the BP86-D3/def2-TZVP level of theory.

Complex	ΔE	d[a]	$\alpha_{A\cdots X–R}$[a]	ρ[b]	$\nabla^2\rho$[b]	V[b]	G[b]	–G/V
**21** HCN···Br-CH_3	−0.3	3.231	179.9	0.81	3.08	−0.45	0.61	0.74
**22** H_3N···Br–CH_3	−2.1	2.924	179.9	1.84	5.45	−1.12	1.24	0.90
**23** $(CH_3)_2O$···Br–CH_3	−2.6	2.981	171.2	1.38	4.98	−0.86	1.05	0.82
**24** NH_2HCO···Br–CH_3	−2.9	3.023	174.7	1.26	4.48	−0.75	0.93	0.80
**25** HCN···I-CH_3	−1.2	3.193	179.9	1.14	3.90	−0.65	0.81	0.80

**26** $H_3N\cdots I–CH_3$	−4.4	2.923	179.9	2.28	5.92	−1.44	1.46	0.99
**27** $(CH_3)_2O\cdots I–CH_3$	−3.7	2.984	176.3	1.72	5.56	−1.09	1.24	0.88
**28** $NH_2HCO\cdots I–CH_3$	−4.3	3.019	176.0	1.59	5.13	−0.96	1.12	0.86
**29** $HCN\cdots AsH_2\text{-}CH_3$	−1.7	3.524	179.9	0.61	1.55	−0.24	0.31	0.76
**30** $H_3N\cdots AsH_2\text{-}CH_3$	−1.8	3.123	163.6	1.30	3.35	−0.69	0.76	0.90
**31** $(CH_3)_2O\cdots AsH_2\text{-}CH_3$	−2.7	3.117	163.0	1.11	3.42	−0.59	0.72	0.82
**32** $NH_2HCO\cdots AsH_2\text{-}CH_3$	−3.8	3.179	161.8	0.99	2.99	−0.50	0.62	0.80
**33** $HCN\cdots SbH_2\text{-}CH_3$	−0.9	3.436	162.5	0.73	2.17	−0.33	0.44	0.75
**34** $H_3N\cdots SbH_2\text{-}CH_3$	−3.2	3.079	159.8	1.67	3.79	−0.91	0.93	0.98
**35** $(CH_3)_2O\cdots SbH_2\text{-}CH_3$	−2.7	3.166	158.8	1.18	3.35	−0.62	0.73	0.85
**36** $NH_2HCO\cdots SbH_2\text{-}CH_3$	−3.2	3.218	161.9	1.10	3.02	−0.54	0.65	0.83

**37** $\mathrm{HCN\cdots Br\text{-}CH_3}$	−3.9	2.919	179.9	1.53	5.50	−0.98	1.18	0.83
**38** $\mathrm{H_3N\cdots Br\text{–}CH_3}$	−8.1	2.693	179.9	2.88	7.78	−1.92	1.93	0.99
**39** $\mathrm{(CH_3)_2O\cdots Br\text{–}CH_3}$	−6.0	2.697	177.8	2.41	8.24	−1.76	1.91	0.92
**40** $\mathrm{NH_2HCO\cdots Br\text{–}CH_3}$	−6.5	2.691	177.1	2.40	8.22	−1.74	1.90	0.92
**41** $\mathrm{HCN\cdots I\text{-}CN}$	−5.4	2.898	179.9	2.01	6.39	−1.34	1.47	0.91
**42** $\mathrm{H_3N\cdots I\text{–}CN}$	−11.4	2.722	179.9	3.31	7.60	−2.28	2.09	1.09
**43** $\mathrm{(CH_3)_2O\cdots I\text{–}CN}$	−8.3	2.727	179.1	2.76	8.32	−2.06	2.07	0.99
**44** $\mathrm{NH_2HCO\cdots I\text{–}CN}$	−9.3	2.710	177.6	2.83	8.45	−2.11	2.11	1.00
**45** $\mathrm{HCN\cdots AsH_2\text{-}CN}$	−3.5	3.033	161.2	1.23	3.89	−0.70	0.84	0.84
**46** $\mathrm{H_3N\cdots AsH_2\text{-}CN}$	−6.7	2.801	164.2	2.32	5.06	−1.39	1.33	1.05
**47** $\mathrm{(CH_3)_2O\cdots AsH_2\text{-}CN}$	−5.6	2.799	161.2	1.91	5.57	−1.23	1.31	0.94

**48** $NH_2HCO\cdots AsH_2$-CN	−6.5	2.787	162.5	1.95	5.60	−1.24	1.32	0.94
**49** $HCN\cdots SbH_2$-CN	−4.4	3.025	161.3	1.51	4.23	−0.87	0.97	0.90
**50** $H_3N\cdots SbH_2$-CN	−9.0	2.825	161.3	2.59	5.24	−1.59	1.45	1.10
**51** $(CH_3)_2O\cdots SbH_2$-CN	−6.3	2.828	159.6	2.07	5.76	−1.36	1.40	0.97
**52** $NH_2HCO\cdots SbH_2$-CN	−7.1	2.803	162.1	2.20	5.87	−1.44	1.45	0.99

[a]Values given as the shortest distance between the Lewis base and the Pnictogen/Halogen atom.
[b]Values multiplied by 100.

Co NCIs in the Cambridge Structural Database

With the purpose to compare our results with those Cbl structures present in the Cambridge Structural Database, we conducted a search involving Co corrin systems with the same conditions as the one carried out in the PDB (see above). As a result, we found a total number of 50 X-ray structures, 27 of them belonging to CNCbl. Most of the cases exhibited an octahedral Co coordination environment, with the two L···Co axial distances ranging between 1.8 and 2.1Å, likely due to the presence of small ligands with a high affinity for metal coordination (e.g. a chloride ion, an isocyanate group or even a platinum coordination complex) which are not common in a biological context, as well as to the different chemical environment (in terms of composition and flexibility) offered inside a protein's active site. Besides, due to the fact that most structures involved CNCbl, the Co coordination was even more favored, since it exhibited one of the most electropositive σ-hole MEP values, as shown in Figure 2 of the manuscript.

Only one example was found to exhibit a L···Co distance that can be interpreted as a plausible noncovalent interaction, involving structure TUDDEE (see Figure S3 below). We computed the interaction energy of the O···Co contact, resulting in –15.3 kcal·mol^{-1}, in line with the results discussed in the main text.

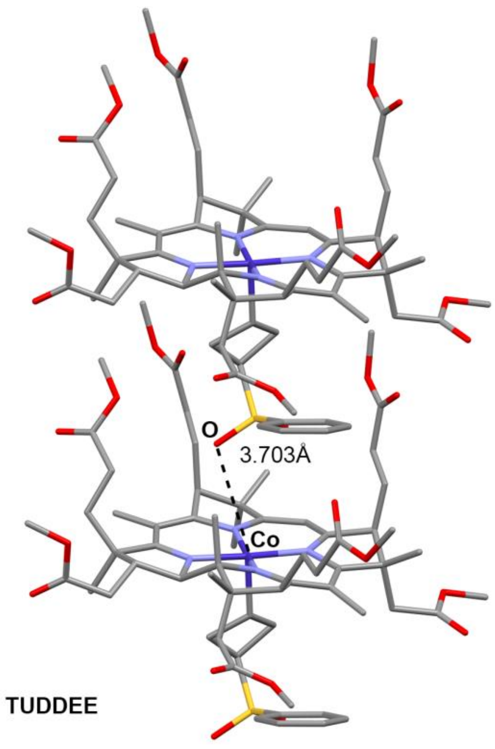

Figure S3. Partial view of the X-ray crystal structure TUDDEE, exhibiting a noncovalent O⋯Co contact. The O⋯Co distance is also indicated along with the CSD code.

Cartesian coordinates of the PDB models

2BB5

C	59.2810000	20.8460000	28.5610000
C	59.1210000	19.5170000	29.3040000
O	59.6070000	19.3740000	30.4370000
N	58.4580000	18.5520000	28.6560000
C	58.3220000	17.1770000	29.1630000
C	59.6600000	16.5790000	29.6240000
O	59.7070000	15.7790000	30.5640000
C	57.6900000	16.2820000	28.0910000
C	56.2670000	16.6340000	27.7750000
N	55.8990000	17.2950000	26.6220000
C	55.1220000	16.4170000	28.4660000
C	54.5870000	17.4650000	26.6140000
N	54.0920000	16.9460000	27.7240000

N	60.7360000	16.9830000	28.9520000
C	62.0930000	16.5990000	29.3110000
Co	51.6870000	16.1270000	28.9010000
N	51.5260000	17.3480000	30.3170000
N	50.6730000	17.2790000	27.7830000
N	52.0090000	14.7870000	27.6060000
N	52.7980000	15.1930000	30.1150000
C	51.9930000	16.8950000	31.6580000
C	50.9100000	16.1100000	32.3860000
C	52.3470000	18.2550000	32.2970000
C	52.3890000	18.2720000	33.8130000
C	53.7240000	18.7020000	31.7760000
C	51.3040000	19.2030000	31.6520000
C	49.9950000	19.4920000	32.4120000
C	51.1000000	18.5570000	30.3270000
C	50.5400000	19.2380000	29.1830000
C	50.3830000	20.7230000	29.2870000
C	50.2050000	18.5540000	28.0620000
C	49.2840000	18.9960000	26.9020000
C	47.9600000	19.6140000	27.2690000
C	50.0700000	19.9420000	25.9540000
C	49.1550000	17.7010000	26.0900000
C	47.8170000	16.9750000	26.2150000
C	50.3210000	16.8960000	26.5690000
C	50.9180000	15.9420000	25.8540000
C	51.7000000	14.9390000	26.3480000
C	52.3260000	13.8650000	25.5070000
C	53.6880000	14.3890000	25.0620000
C	51.5310000	13.5260000	24.2740000
C	52.4940000	12.7540000	26.5530000
C	51.3940000	11.7380000	26.6420000
C	52.6050000	13.5520000	27.8140000
C	53.2120000	13.1440000	28.9620000
C	53.7720000	11.7520000	29.0060000
C	53.3430000	14.0130000	30.0960000
C	54.1250000	13.7550000	31.3960000
C	55.5970000	13.8630000	31.1010000
C	53.8760000	12.4280000	32.1380000
C	52.4490000	11.9840000	32.4260000

C	52.3580000	10.5340000	32.7680000
O	52.7050000	9.6610000	31.9880000
N	51.8670000	10.2690000	33.9740000
C	53.6300000	14.8990000	32.2940000
C	54.5740000	15.3280000	33.3930000
C	53.1480000	15.9600000	31.3200000
C	51.8290000	8.9470000	34.5440000
C	50.5460000	8.2170000	34.3290000
C	50.3430000	7.1650000	35.3750000
O	49.4520000	9.1290000	34.4850000
O	47.7170000	7.8480000	33.2360000
O	47.3130000	10.2100000	34.0370000
P	48.2400000	9.1930000	33.5090000
O	49.0310000	9.7020000	32.2210000
C	48.3370000	10.0440000	31.0560000
C	47.8450000	11.4830000	31.0570000
O	48.5230000	12.2170000	32.0400000
C	48.2240000	11.9820000	29.6800000
O	49.3160000	11.2080000	29.2750000
C	49.3020000	9.9260000	29.9140000
C	48.9450000	8.8730000	28.9330000
O	49.8330000	7.7790000	28.9880000
N	48.6170000	13.3670000	29.6430000
C	47.8360000	14.4660000	29.9120000
C	49.8350000	13.8390000	29.3410000
N	49.9360000	15.1300000	29.3810000
C	48.6610000	15.5690000	29.7490000
C	48.1380000	16.8050000	29.9640000
C	46.8070000	16.9470000	30.3370000
C	46.2780000	18.3090000	30.5600000
C	46.0010000	15.8150000	30.4930000
C	44.5690000	15.9420000	30.8890000
C	46.5270000	14.5660000	30.2750000
H	60.3555603	21.0155985	28.4000673
H	57.7282550	15.2512477	28.4740885
H	58.3144583	16.3011200	27.1819017
H	54.9675576	15.9144032	29.4092077
H	54.0185085	17.9428818	25.8182295
H	57.6939671	17.1663788	30.0680119

H	57.9634631	18.7867689	27.8052659
H	62.0231531	15.7690981	30.0252517
H	60.6139911	17.7121623	28.2626639
H	51.2230882	15.8648694	33.4094346
H	49.9689766	16.6700384	32.4403510
H	50.6913089	15.1730637	31.8567921
H	51.4346939	17.9742967	34.2697545
H	52.6235646	19.2852461	34.1762441
H	53.1700591	17.6042147	34.1956851
H	54.5404939	18.1021273	32.2010812
H	53.7920754	18.6579981	30.6787990
H	51.7839208	20.1850270	31.5023320
H	49.4089149	18.5825250	32.6017530
H	49.3574974	20.1766596	31.8376416
H	49.6664838	21.0456919	30.0585429
H	50.0566095	21.1715978	28.3442446
H	51.3487676	21.1913640	29.5380536
H	47.3735110	18.9690861	27.9373630
H	48.0837585	20.5829781	27.7734689
H	47.3654590	19.7965433	26.3615771
H	50.3298898	20.9014458	26.4244219
H	51.0079907	19.4669156	25.6267388
H	49.2812387	17.9240212	25.0168305
H	47.5853438	16.7192610	27.2604688
H	47.8438104	16.0355298	25.6427188
H	50.6110827	15.8568631	24.8101811
H	54.3276096	14.6301797	25.9258900
H	54.2074484	13.6339725	24.4501367
H	53.5756702	15.3024498	24.4586884
H	50.4859739	13.2710907	24.5034015
H	51.9761980	12.6669471	23.7476526
H	51.5241644	14.3663528	23.5640154
H	53.4401220	12.2242988	26.3567964
H	51.5551985	11.0449347	27.4785274
H	50.4132021	12.2073067	26.8148550
H	54.8180977	11.7411521	29.3462570
H	53.2206552	11.0959216	29.7015588
H	53.7582458	11.2654240	28.0268601
H	56.1992659	13.6176993	31.9884848

H	55.9017651	14.8747280	30.7938318
H	55.9071322	13.1713069	30.3046489
H	54.4251891	12.5010994	33.0926170
H	54.3699598	11.6069482	31.5970526
H	51.9879107	12.6003875	33.2131122
H	51.8144022	12.1261129	31.5326275
H	52.7256793	14.5053649	32.7809981
H	54.9529579	14.4551849	33.9453325
H	55.4468420	15.8805788	33.0115624
H	54.0027751	16.5871605	31.0238114
H	52.0138350	9.0552051	35.6262577
H	52.6612103	8.3701036	34.1146434
H	50.5129681	7.7883930	33.3132474
H	51.1566308	6.4202077	35.3872981
H	50.2830753	7.6413336	36.3654311
H	49.3908935	6.6528464	35.1764547
H	47.4884050	9.3462671	30.9327955
H	46.7522981	11.5251579	31.2164291
H	48.2541569	11.7896916	32.8838345
H	47.3713490	11.8598542	28.9795405
H	50.3171098	9.7379347	30.2957168
H	47.8909659	8.5499905	29.0378868
H	49.0508499	9.2951590	27.9204953
H	49.7259089	7.3580692	29.8500308
H	50.6426549	13.1491080	29.1172441
H	48.7521806	17.6952313	29.8528046
H	45.8660666	18.4320534	31.5761069
H	47.0578605	19.0716103	30.4263175
H	45.4529525	18.5532180	29.8684127
H	43.9975144	16.5483977	30.1663122
H	44.4597929	16.4412115	31.8662785
H	44.0811234	14.9609409	30.9632279
H	45.9108893	13.6752405	30.4126682
H	56.5356493	17.5186342	25.8666411
H	62.6233848	17.4357692	29.7926599
H	62.6541624	16.2732030	28.4227151
H	58.9202172	21.6519495	29.2141021
H	58.7588575	20.8936351	27.5943392
H	51.3401780	10.9830580	34.4583031

H	49.4646567	20.1620234	25.0611037
H	46.9992285	17.5877862	25.8127904
H	54.0691436	15.9665966	34.1287108
H	50.2081487	19.9693802	33.3791952
H	53.9103705	19.7441768	32.0806499
H	51.3223893	11.1398995	25.7212920

2XIQ

Co	12.6943727	84.1502613	120.6673379
N	11.0364577	83.3295936	121.0156609
N	11.8008397	85.8588222	120.7515347
N	14.3674231	84.8296759	120.1111834
N	13.2885828	82.3980758	120.4931738
C	10.9295847	81.9440654	120.5152199
C	10.7686374	82.0280155	118.9991017
C	9.6626472	81.4097088	121.2739260
C	8.8693984	80.3718134	120.4906089
C	10.0331147	80.8287130	122.6437126
C	8.8640130	82.7347359	121.5552266
C	7.7464881	83.0990713	120.5739987
C	9.9670516	83.7570667	121.5617301
C	9.9525972	85.1495950	122.1628638
C	8.6147603	85.5670995	122.7571674
C	10.6412774	86.1383317	121.2344984
C	10.0552489	87.4151153	120.6291035
C	9.1535079	86.9484644	119.4709333
C	9.2842583	88.3652865	121.5385194
C	11.3592909	88.0892335	120.1321022
C	11.2302963	89.0781370	118.9908783
C	12.2669469	86.9020363	119.8340693
C	13.7338545	87.1650340	119.9260053
C	14.6581989	86.1909900	120.0051855
C	16.1633592	86.3764752	119.9454178
C	16.6051097	87.6002691	119.1548795
C	16.7204137	86.4616862	121.3735324
C	16.5675413	85.0177601	119.3182225
C	16.6302963	85.0128940	117.7903636
C	15.4559625	84.0923515	119.7808711
C	15.5584588	82.7017288	119.7616072

C	16.8611421	82.0596006	119.3521517
C	14.4291709	81.8909389	120.0887652
C	14.3772740	80.3638612	120.1234160
C	15.1351146	79.8812603	121.3703916
C	14.8992441	79.6562000	118.8639199
C	14.4585924	80.2770095	117.5344642
C	14.4487845	79.1908091	116.4710534
O	15.4836637	78.7120487	116.0302877
N	13.2124000	78.7621027	116.1589685
C	12.8429387	80.0882535	120.2540189
C	12.4897793	78.7980510	120.9715773
C	12.3171125	81.3701309	120.8890378
C	12.9705255	77.5412036	115.4385793
C	11.5192581	77.1084005	115.6203614
C	11.2160380	75.8151668	114.8917174
O	11.2399248	76.9205618	117.0018545
O	10.1739970	77.5800993	119.2181693
O	10.8970622	79.4154399	117.4639313
P	10.3400293	78.0431503	117.8047929
O	8.9076501	77.9587483	117.0015002
C	8.0614719	76.8726646	117.2834213
N	9.7794542	87.7430440	125.6060222
C	10.0532637	86.4631406	125.8450569
N	11.1472131	85.7853414	125.4957629
C	12.0164847	86.5532497	124.8273546
N	13.2384821	86.1971697	124.3334520
C	13.7290269	87.3012693	123.6764431
N	12.9221354	88.3321392	123.7324880
C	11.8477652	87.8886468	124.4611266
C	10.6582894	88.4941538	124.9252435
N	10.3753409	89.7851919	124.6932471
C	13.8143255	84.8983443	124.5842042
C	13.0263339	83.7524398	123.9350310
C	14.1391845	82.7136460	123.6613238
C	15.4163897	83.4219991	124.1578427
C	16.6638478	83.1183632	123.3761664
O	15.0971597	84.8163329	124.0773837
O	12.0299944	83.2150858	124.7407044
O	13.9007480	81.4884379	124.2719602

H	10.6781074	81.0333546	118.5277099
H	9.8885325	82.6294849	118.7324888
H	11.6522008	82.5171547	118.5592355
H	7.9755524	80.0722523	121.0612255
H	9.4572949	79.4605472	120.2981277
H	8.5371177	80.7366716	119.5103786
H	10.6364238	79.9179774	122.5408654
H	10.5868575	81.5452573	123.2723573
H	8.4106839	82.6719840	122.5591252
H	8.1198591	83.2215312	119.5484775
H	7.2437570	84.0314080	120.8679794
H	10.6462272	85.0522378	123.0191709
H	8.2361078	84.7652782	123.4053602
H	8.7243493	86.4621970	123.3803529
H	7.8512463	85.7694305	121.9956111
H	8.7245088	87.8175225	118.9506196
H	8.3162999	86.3392554	119.8432994
H	9.7016829	86.3432201	118.7330515
H	8.2788441	88.0053598	121.7872275
H	9.8248347	88.5727798	122.4701005
H	11.7941412	88.6016734	121.0102844
H	10.5324025	89.8931735	119.2357845
H	12.2044878	89.5400839	118.7708083
H	12.0457336	86.5097086	118.8161593
H	14.0610069	88.2022075	119.8371106
H	16.1543501	87.6330614	118.1538377
H	16.3201723	88.5241481	119.6813011
H	17.7006437	87.6154049	119.0446259
H	16.4437470	87.4219031	121.8364923
H	16.3369098	85.6523684	122.0107314
H	17.8203621	86.4038390	121.3628205
H	17.5422648	84.6977171	119.7162598
H	15.6773363	85.3548165	117.3575524
H	16.8195535	83.9980812	117.4139837
H	17.0937876	81.1795138	119.9633887
H	17.7065581	82.7487966	119.4684691
H	16.8615176	81.7212586	118.3032744
H	15.1368511	78.7824226	121.4034791
H	14.6750740	80.2431942	122.3021108

H	16.1842971	80.2089269	121.3590088
H	14.5068966	78.6262932	118.9214730
H	15.9923932	79.5463307	118.8751063
H	13.4578464	80.7264082	117.6263776
H	15.1537696	81.0716254	117.2252022
H	12.4281485	80.0169362	119.2362359
H	12.6970890	78.8518771	122.0515862
H	11.4429114	78.5168836	120.7940634
H	12.3640406	81.2879550	121.9898910
H	13.6476851	76.7485548	115.8041794
H	13.1809060	77.6601142	114.3598606
H	10.8663366	77.9088057	115.2246515
H	11.8270223	74.9960650	115.3011867
H	10.1569875	75.5517674	115.0225049
H	11.4232593	75.9064875	113.8146664
H	8.4268248	75.9386764	116.8179294
H	9.2872118	85.8986786	126.3880443
H	14.6898288	87.2606676	123.1671701
H	13.8010368	84.7360339	125.6831813
H	11.5515498	83.9592390	125.1744012
H	13.1120827	81.6062009	124.8252302
H	9.1578151	89.3244442	121.0126244
H	10.8804735	88.5950416	118.0660811
H	13.0697076	77.9608555	120.5541935
H	17.4338005	85.6647070	117.4182742
H	6.9857254	82.3068541	120.5594567
H	9.1126787	80.5513113	123.1809886
H	7.0656294	77.1005586	116.8728395
H	7.9835284	76.7018659	118.3700426
H	12.3940173	79.1689328	116.6472297
H	11.0574168	90.3751982	124.2391994
H	9.5330535	90.1822673	125.0853541
H	17.5170782	83.7069694	123.7422697
H	16.9019664	82.0501347	123.4836536
H	16.5086363	83.3233109	122.3059636
H	14.2288040	82.5730818	122.5748534
H	12.6109353	84.1325928	122.9769080
H	15.5485129	83.1496496	125.2238948

6D5K

C	-10.54200000	43.79600000	-13.51500000
C	-12.00600000	43.52400000	-13.22600000
O	-12.47700000	43.69600000	-12.11000000
N	-12.72400000	43.07300000	-14.24400000
C	-14.13600000	42.74100000	-14.11400000
C	-14.30500000	41.39000000	-13.43800000
O	-13.68600000	40.40600000	-13.85300000
C	-14.79000000	42.65700000	-15.48400000
C	-15.02300000	43.96400000	-16.14300000
C	-15.90100000	44.89000000	-15.59400000
C	-14.42800000	44.23900000	-17.37200000
C	-16.15500000	46.10200000	-16.23700000
C	-14.66800000	45.43100000	-18.02100000
C	-15.53500000	46.37000000	-17.45100000
N	-15.17000000	41.32700000	-12.44200000
C	-15.45200000	40.06900000	-11.75600000
N	-26.27400000	52.14600000	-16.45800000
C	-25.55200000	51.45900000	-17.34600000
N	-24.28100000	51.64200000	-17.71100000
C	-23.75200000	52.70200000	-17.09700000
N	-22.49200000	53.22500000	-17.26500000
C	-22.44900000	54.34800000	-16.49000000
N	-23.55400000	54.58100000	-15.81600000
C	-24.37900000	53.53100000	-16.18700000
C	-25.70200000	53.19500000	-15.82400000
N	-26.37600000	53.78600000	-14.83500000
C	-21.41000000	52.65900000	-18.07200000
C	-20.31800000	53.65500000	-18.48600000
C	-19.22500000	53.34800000	-17.47200000
C	-19.35700000	51.83900000	-17.38700000
C	-18.70400000	51.18800000	-16.19100000
O	-20.77900000	51.61400000	-17.34800000
O	-19.86500000	53.26500000	-19.77500000
O	-17.94600000	53.67300000	-17.99500000
Co	-17.52800000	49.50400000	-15.94800000
N	-16.05900000	50.40800000	-15.20700000
N	-17.96000000	48.82900000	-14.20600000
N	-18.77000000	48.40800000	-16.91300000

N	-16.84900000	50.15900000	-17.58800000
C	-14.99200000	50.83500000	-16.09500000
C	-14.05300000	49.66000000	-16.29100000
C	-14.36900000	52.06900000	-15.33500000
C	-12.90500000	52.32400000	-15.71500000
C	-14.61200000	51.65600000	-13.83800000
C	-13.50400000	50.77700000	-13.23700000
C	-15.86200000	50.82500000	-13.98900000
C	-16.81100000	50.46000000	-12.84400000
C	-16.68400000	51.32300000	-11.62300000
C	-17.71200000	49.42500000	-12.97100000
C	-18.69000000	48.86700000	-11.94100000
C	-18.15400000	48.53400000	-10.53500000
C	-19.85900000	49.85500000	-11.90500000
C	-19.16900000	47.56900000	-12.64300000
C	-18.33800000	46.29000000	-12.46400000
C	-19.00100000	47.99700000	-14.07800000
C	-19.94300000	47.61300000	-15.03700000
C	-19.85100000	47.89300000	-16.37300000
C	-20.90600000	47.53100000	-17.39300000
C	-21.46700000	46.13900000	-17.09000000
C	-22.04800000	48.55500000	-17.40700000
C	-20.12400000	47.44200000	-18.70300000
C	-19.66800000	45.99100000	-18.91400000
C	-18.98000000	48.38100000	-18.32000000
C	-18.33500000	49.12500000	-19.26200000
C	-18.82000000	49.04700000	-20.68000000
C	-17.11000000	49.93700000	-18.86100000
C	-16.17100000	50.67400000	-19.80200000
C	-16.90600000	51.90400000	-20.36800000
C	-15.56000000	49.81100000	-20.93700000
C	-15.23200000	48.44900000	-20.32900000
C	-14.72000000	47.49700000	-21.38000000
O	-15.45300000	46.66800000	-21.90300000
N	-13.42800000	47.57500000	-21.66500000
C	-15.03900000	51.04400000	-18.82000000
C	-14.27700000	52.36200000	-19.06200000
C	-15.74100000	51.13000000	-17.45500000
C	-12.76600000	46.68400000	-22.60200000

C	-12.38600000	45.38600000	-21.89600000
C	-11.93500000	44.37700000	-22.92000000
O	-11.28600000	45.68100000	-21.06100000
O	-9.53184253	45.46067262	-22.95401992
O	-9.36440062	47.47133899	-21.43842434
P	-9.82379574	46.02079033	-21.61065010
O	-9.00805639	45.49316728	-20.37291296
C	-9.23224216	44.28987000	-19.67871889
H	-10.34955700	44.08146870	-14.55957290
H	-15.75982910	42.14700880	-15.35067270
H	-14.18960290	41.98342310	-16.11458630
H	-16.39940920	44.66838270	-14.64445780
H	-13.73269460	43.52750770	-17.83169460
H	-16.85035690	46.82292360	-15.79982970
H	-14.18004660	45.63135260	-18.97844320
H	-15.75637540	47.30421440	-17.96843530
H	-14.60195980	43.53166570	-13.50239540
H	-15.04572300	39.26293960	-12.37896740
H	-26.07389940	50.62597110	-17.83175200
H	-21.55879550	54.97487230	-16.43200460
H	-26.00930260	54.62374770	-14.40706590
H	-21.89552110	52.23846250	-18.96749750
H	-20.65364910	54.70393470	-18.47947040
H	-19.39737700	53.84934430	-16.50628850
H	-18.96851660	51.44386450	-18.34105670
H	-19.49759890	50.94550060	-15.46556780
H	-18.01993200	51.90630360	-15.71375440
H	-20.41469280	53.69199450	-20.43976370
H	-18.09417890	53.85343990	-18.93377940
H	-13.67720960	49.24106130	-15.35529450
H	-13.18962380	49.92242830	-16.91806760
H	-14.59173270	48.84453430	-16.79472400
H	-12.47899450	53.12690190	-15.09264000
H	-12.27945040	51.43424460	-15.57536270
H	-12.81188250	52.64849190	-16.75659210
H	-14.77477880	52.53430720	-13.19703810
H	-13.75494470	50.51096820	-12.19896250
H	-13.35097530	49.84041010	-13.77650790
H	-17.46587680	51.11917740	-10.88529840

H	-15.71495690	51.19798190	-11.11177100
H	-16.76803740	52.38780710	-11.89342690
H	-18.90672470	47.93694480	-9.99813790
H	-17.95829560	49.42915390	-9.92932760
H	-17.22375440	47.95186410	-10.58741250
H	-19.54434960	50.83383880	-11.51676740
H	-20.27710580	50.00956470	-12.91180840
H	-20.22137340	47.36386690	-12.39779330
H	-18.39049590	45.90644520	-11.43439120
H	-17.28025500	46.46400170	-12.71500460
H	-20.80372730	47.05434550	-14.67290230
H	-20.70524690	45.39189830	-16.85311900
H	-22.14355690	46.18519330	-16.21928550
H	-22.08687880	45.77808520	-17.92183100
H	-22.53311940	48.61355490	-16.42115930
H	-22.80709700	48.25512990	-18.14473980
H	-21.68966060	49.56272920	-17.65922170
H	-20.70062590	47.81228120	-19.55783840
H	-19.10666200	45.61223060	-18.05089420
H	-20.50483270	45.32616830	-19.14378560
H	-19.87200330	49.36217210	-20.75176640
H	-18.24866060	49.69467050	-21.35182900
H	-18.73910270	48.02770850	-21.08907640
H	-17.13762590	52.59047960	-19.55909250
H	-16.26545050	52.41782600	-21.10028160
H	-17.84374610	51.62632430	-20.86416590
H	-14.65942250	50.31817060	-21.31798510
H	-16.24994090	49.68700530	-21.78336990
H	-14.50176220	48.53039240	-19.51143800
H	-16.14167110	47.98194010	-19.93517030
H	-14.32006050	50.21647220	-18.79535340
H	-13.38204840	52.40739400	-18.43320320
H	-14.90482280	53.23741360	-18.84356040
H	-16.21528600	52.12323290	-17.35392110
H	-11.86087260	47.16604140	-22.99511080
H	-13.47039590	46.47326460	-23.41774640
H	-13.24327290	45.00703570	-21.31185450
H	-11.64847390	43.44632180	-22.40926460
H	-11.04922420	44.75760400	-23.45043540

H	-12.72960070	44.14957950	-23.64894590
H	-10.26224913	44.23775788	-19.27414850
H	-14.96305920	40.03035020	-10.76932020
H	-16.53623270	39.93302240	-11.62689530
H	-9.96088550	42.88674490	-13.29540150
H	-10.19875560	44.58829100	-12.83882290
H	-9.07770986	43.39378015	-20.31321940
H	-8.52275066	44.23869920	-18.83722566
H	-20.66607490	49.47765540	-11.25667410
H	-13.93075450	52.44459500	-20.10444570
H	-18.72102240	45.50911380	-13.13690290
H	-18.97486270	45.93842290	-19.77045990
H	-12.54782590	51.31876470	-13.20719850
H	-14.99092400	52.95075820	-15.55824310
H	-27.34002930	53.52143140	-14.68708830
H	-12.27096590	42.84987660	-15.12174310
H	-15.49340370	42.19035090	-12.02614130
H	-12.82723960	48.14476450	-21.08336630

6OXC

C	7.2820000	109.7890000	100.3590000
C	5.9960000	109.7320000	99.6130000
N	4.9540000	110.6060000	99.8510000
C	5.5840000	108.9130000	98.6200000
C	3.9710000	110.3410000	99.0160000
N	4.3340000	109.3250000	98.2570000
H	7.7683482	110.7728708	100.2627762
H	7.9789564	109.0462454	99.9494713
H	4.9844121	111.3526189	100.5338145
H	6.1079084	108.1080872	98.1112974
H	3.0267544	110.8778263	98.9511833
Co	2.9780000	108.4040000	96.6820000
N	2.8660000	110.1920000	96.0820000
N	1.6820000	108.7950000	98.0460000
N	3.3220000	106.6190000	97.1940000
N	4.3670000	108.3080000	95.3860000
C	3.6150000	110.5400000	94.8820000
C	4.7520000	111.1970000	95.6860000
C	2.8270000	111.5780000	94.0620000

C	3.7570000	112.7090000	93.5430000
C	2.0840000	110.9250000	92.8970000
C	1.7760000	112.0680000	95.0810000
C	2.0960000	113.3910000	95.8060000
C	1.6780000	110.9440000	96.0790000
C	0.5920000	110.7550000	97.0340000
C	-0.5090000	111.7890000	96.9590000
C	0.5940000	109.7230000	97.9130000
C	-0.4410000	109.5040000	99.0380000
C	-0.7150000	110.7870000	99.8520000
C	-1.7530000	108.9870000	98.4910000
C	0.1800000	108.3750000	99.8880000
C	0.7000000	108.7510000	101.2910000
C	1.2430000	107.8400000	98.9720000
C	1.7850000	106.4910000	99.1070000
C	2.7770000	106.0200000	98.3440000
C	3.1490000	104.5970000	98.5520000
C	2.7140000	103.6730000	97.4410000
C	3.4590000	103.9670000	99.8820000
C	4.6310000	104.8680000	98.0350000
C	5.7190000	105.3110000	99.0290000
C	4.5520000	105.9890000	97.0100000
C	5.4230000	106.2250000	95.8590000
C	6.4510000	105.1750000	95.5010000
C	5.2180000	107.2750000	95.0350000
C	5.9590000	107.6680000	93.7340000
C	5.2750000	106.7880000	92.6970000
C	7.4750000	107.4610000	93.6950000
C	8.2350000	108.1970000	94.7920000
C	9.6110000	107.5810000	94.9100000
O	9.8520000	106.8680000	95.8680000
N	10.4860000	107.8480000	93.9380000
C	5.5480000	109.1490000	93.4880000
C	5.5300000	109.7290000	92.0650000
C	4.3700000	109.3260000	94.4180000
C	11.7790000	107.2550000	93.9520000
C	12.7390000	108.0520000	94.8430000
C	14.1250000	107.4470000	94.7670000
O	12.7230000	109.3710000	94.3110000

O	11.8540000	110.4590000	96.5130000
O	12.0490000	111.7330000	94.3810000
P	12.6160000	110.6610000	95.2480000
O	14.1620000	111.0680000	95.5870000
C	14.9780000	111.4070000	94.4980000
H	5.1806204	110.4446217	96.3559335
H	4.4226202	112.0331883	96.3154187
H	5.5747973	111.5684343	95.0519015
H	3.1713605	113.4284546	92.9493878
H	4.5319439	112.2984025	92.8834581
H	4.2547775	113.2577833	94.3469689
H	1.4617647	110.0900649	93.2562338
H	2.7753425	110.5289506	92.1402074
H	0.8129106	112.2037827	94.5630614
H	3.0214019	113.3288005	96.3910352
H	1.2839103	113.6595874	96.4933985
H	-0.3081770	112.6575036	97.6052739
H	-1.4834417	111.3835906	97.2503764
H	-0.6321248	112.1634755	95.9346921
H	-1.3228864	111.5039421	99.2919149
H	0.2113834	111.2931819	100.1619364
H	-1.2823020	110.5333432	100.7589485
H	-1.6297887	108.0060451	98.0194374
H	-2.1997918	109.6488049	97.7370316
H	-0.5730993	107.5843686	100.0393392
H	1.1581354	107.8721636	101.7682028
H	-0.1227178	109.0937776	101.9321136
H	1.3606990	105.8689711	99.8951871
H	1.6308401	103.4561883	97.4663292
H	3.2452538	102.7138412	97.5193364
H	2.9229841	104.1097325	96.4553377
H	2.5645578	103.5415963	100.3729294
H	3.8903863	104.6881308	100.5858573
H	4.1609975	103.1345962	99.7388679
H	5.0177892	103.9527629	97.5590947
H	5.4156018	106.2044996	99.5972899
H	6.6304883	105.5698568	98.4699236
H	6.3114513	104.2612991	96.0921249
H	7.4868298	105.5119807	95.6763729

H	6.3671202	104.8793952	94.4471726
H	5.4301349	105.7178919	92.8955738
H	5.6634846	106.9872861	91.6852381
H	4.1871515	106.9656193	92.6725526
H	7.8283061	107.8030961	92.7073222
H	7.7068379	106.3902815	93.7276583
H	7.7524465	108.0685595	95.7707006
H	8.3077055	109.2775637	94.5893021
H	10.3982994	108.7275793	93.4398210
H	5.4743551	110.8233140	92.1006241
H	4.6899763	109.3558505	91.4565703
H	12.1797444	107.2349486	92.9260503
H	11.6872297	106.2240769	94.3229533
H	12.3529992	108.0754381	95.8765080
H	14.8307366	108.0465293	95.3610963
H	14.4814230	107.4363608	93.7246574
H	14.1301885	106.4163667	95.1552261
H	15.3138146	110.5128511	93.9369417
N	-5.2610000	111.0700000	95.3200000
C	-5.2090000	109.8990000	96.0370000
N	-4.1800000	109.0200000	95.8290000
C	-3.2060000	109.3010000	94.9210000
N	-2.1030000	108.6170000	94.5220000
C	-1.5000000	109.3720000	93.5790000
N	-2.2100000	110.5020000	93.3860000
C	-3.2640000	110.4540000	94.2190000
C	-4.2950000	111.3420000	94.4260000
N	-4.6630000	112.6570000	93.8360000
C	-1.8100000	107.4240000	95.0970000
C	-1.2330000	106.3570000	94.1420000
C	-0.3290000	105.6540000	94.8870000
C	0.3540000	106.7720000	95.8190000
C	1.4170000	107.4530000	94.9470000
O	-0.6280000	107.5910000	96.1510000
O	-2.3320000	105.5360000	93.5960000
O	-0.8710000	104.6930000	95.8600000
H	-5.9838954	109.6587725	96.7664882
H	-0.5718489	109.1113067	93.0770359
H	-5.6540961	112.8037789	93.9812684

H	-4.3073667	112.8527599	92.9066283
H	-2.6697108	107.0605576	95.6730176
H	-0.7217421	106.8548407	93.3026132
H	0.4302813	105.1297636	94.2792000
H	0.7917341	106.2732917	96.6935420
H	1.6075894	108.4737215	95.3109167
H	1.0531197	107.6121178	93.9184981
H	2.3377636	106.8654096	94.8674027
H	-2.8697665	106.1088126	93.0386093
H	-1.5595493	104.1968305	95.3963166
H	-2.4814231	108.8937750	99.3129386
H	1.4647025	109.5403975	101.2427201
H	6.4648796	109.4658614	91.5492886
H	1.4197534	111.6517975	92.4056930
H	2.1981245	114.2153049	95.0864710
H	5.9908260	104.5206515	99.7415594
H	7.1523136	109.5772100	101.4330467
H	15.8707205	111.9286704	94.8802270
H	14.4343121	112.0684290	93.8011712
H	6.3777691	109.7096815	93.9674313
H	3.5713601	108.9275055	93.7044496

6WGV

Co	-32.1700000	40.5620000	18.3040000
N	-30.4340000	41.2110000	18.1770000
N	-31.9020000	39.4690000	16.7540000
N	-33.9230000	40.0790000	18.6410000
N	-32.1730000	41.7090000	19.8340000
C	-30.1170000	42.5000000	18.8310000
C	-30.6710000	43.6750000	17.9960000
C	-28.5170000	42.4720000	18.9300000
C	-27.8920000	43.8990000	18.9680000
C	-28.0550000	41.6030000	20.1330000
C	-28.1980000	41.6890000	17.6370000
C	-27.8310000	42.4920000	16.3210000
C	-29.4260000	40.8170000	17.4480000
C	-29.4870000	39.7030000	16.5230000
C	-28.1110000	39.1080000	16.1040000
C	-30.6850000	39.1260000	16.1610000

C	-30.8770000	37.9020000	15.2540000
C	-30.0570000	37.8970000	13.9600000
C	-30.6270000	36.6190000	16.1200000
C	-32.4250000	37.9520000	15.0140000
C	-32.8240000	38.7870000	13.7550000
C	-32.8820000	38.7340000	16.2300000
C	-34.1350000	38.5640000	16.7610000
C	-34.6170000	39.1840000	17.8920000
C	-35.9800000	38.8700000	18.4640000
C	-35.8410000	37.4740000	19.1640000
C	-37.0720000	38.7480000	17.3630000
C	-36.1300000	39.9520000	19.5640000
C	-37.3410000	40.9400000	19.2910000
C	-34.8150000	40.6810000	19.5180000
C	-34.5100000	41.5480000	20.5650000
C	-35.5820000	41.9630000	21.5800000
C	-33.1500000	42.0250000	20.6220000
C	-32.7240000	43.3420000	21.3470000
C	-32.6160000	42.6460000	22.7650000
C	-33.6500000	44.5200000	21.4300000
C	-34.0900000	44.9500000	20.0030000
C	-35.2030000	45.9950000	20.1210000
O	-36.3760000	45.6430000	20.0820000
N	-34.7910000	47.2400000	20.2370000
C	-31.2980000	43.6720000	20.8880000
C	-30.2230000	44.0860000	21.9400000
C	-30.9110000	42.3870000	20.1710000
C	-35.8460000	48.2680000	20.3340000
C	-35.8860000	49.0300000	19.0020000
C	-36.8860000	50.1950000	19.0210000
O	-34.5490000	49.5990000	18.8180000
O	-34.2990000	51.7470000	17.4580000
O	-34.7060000	49.4490000	16.3000000
P	-34.0990000	50.2930000	17.4190000
O	-32.4910000	49.9620000	17.3790000
C	-32.1480000	48.6380000	17.1680000
C	-31.7240000	48.1740000	15.7830000
O	-30.9460000	49.0800000	15.0650000
C	-30.9610000	46.8690000	16.1340000

O	-30.3830000	47.1050000	17.3890000
C	-30.8180000	48.3700000	17.9560000
C	-30.9330000	48.2930000	19.4610000
O	-31.8920000	47.2510000	19.7930000
N	-29.8090000	46.5260000	15.2970000
C	-29.6590000	45.3520000	14.5620000
C	-28.7050000	47.2490000	15.0950000
N	-27.8510000	46.6550000	14.2880000
C	-28.4270000	45.4360000	13.9270000
C	-28.0080000	44.3770000	13.1010000
C	-28.8810000	43.2860000	12.9750000
C	-28.5240000	42.0940000	12.1100000
C	-30.1700000	43.2090000	13.6460000
C	-31.0510000	42.0300000	13.4790000
C	-30.5590000	44.2880000	14.4650000
H	-31.7692108	43.6014514	17.9480477
H	-30.2901323	43.6296486	16.9692380
H	-30.4117962	44.6586273	18.4072136
H	-28.1851503	44.5050841	18.1024329
H	-26.7938731	43.8236301	18.9753900
H	-28.1930440	44.4364504	19.8765483
H	-28.5245487	40.6089170	20.1202405
H	-28.2804043	42.0774856	21.0979236
H	-27.3279374	41.0405874	17.8291348
H	-28.6340520	43.1587198	15.9871107
H	-27.6233559	41.7895374	15.5039703
H	-28.1664056	38.0185576	16.0037163
H	-27.3595663	39.3246793	16.8714857
H	-27.7427414	39.5169408	15.1504488
H	-30.0879160	38.8710377	13.4528309
H	-30.4602566	37.1401744	13.2700497
H	-29.0064563	37.6403836	14.1378203
H	-31.2841771	36.6164706	17.0025898
H	-29.5864405	36.5572116	16.4684297
H	-32.8738652	36.9504506	14.9696774
H	-32.4935696	38.2977332	12.8284165
H	-32.3801327	39.7887228	13.8025625
H	-34.7963478	37.8682084	16.2464147
H	-36.7764620	37.2331376	19.6928033

H	-35.0144152	37.4746091	19.8898863
H	-35.6465206	36.6792314	18.4289530
H	-38.0511109	38.5498337	17.8213209
H	-36.8450128	37.9016268	16.7013962
H	-37.1521788	39.6570405	16.7506449
H	-36.2519886	39.4887399	20.5541642
H	-37.4380316	41.7055317	20.0638619
H	-38.2841448	40.3779604	19.2499465
H	-35.1463792	42.2641180	22.5345306
H	-36.2653026	41.1317172	21.7929020
H	-36.1694437	42.8218355	21.2170578
H	-33.5325267	42.1361149	23.0713382
H	-32.3586768	43.3690091	23.5571957
H	-31.8131019	41.8952908	22.7434573
H	-34.5398960	44.3060499	22.0358481
H	-33.1170278	45.3406566	21.9378958
H	-33.2341558	45.3443208	19.4362352
H	-34.5180569	44.0878278	19.4732460
H	-33.8589503	47.5280513	19.9412506
H	-31.3723045	44.4937267	20.1616548
H	-29.3076080	44.3975315	21.4228524
H	-29.9717610	43.2617982	22.6217660
H	-36.7952704	47.7565395	20.5231771
H	-35.6111953	48.9696869	21.1492632
H	-36.0862064	48.3456500	18.1612674
H	-36.6047491	50.9091963	19.8085548
H	-37.9123109	49.8351140	19.1998313
H	-36.8504538	50.7212141	18.0576583
H	-32.9299833	47.9492457	17.5300043
H	-32.6378810	47.9284589	15.2097932
H	-31.3574196	49.9451799	15.2096843
H	-31.6436788	46.0064126	16.1551257
H	-30.1017958	49.1696381	17.6946400
H	-31.2862617	49.2767752	19.8126714
H	-29.9512574	48.0592535	19.9076606
H	-31.9181683	47.1924627	20.7541179
H	-28.5697612	48.2310788	15.5431333
H	-27.0505516	44.4039018	12.5779849
H	-27.5377483	42.2189515	11.6403449

H	-29.2592608	41.9414753	11.3033058
H	-28.4974477	41.1594349	12.6970320
H	-31.3030378	41.8351519	12.4235661
H	-31.9944110	42.1612209	14.0285730
H	-30.5705755	41.1146102	13.8659104
H	-31.5316054	44.2905011	14.9600969
H	-30.3727043	41.7051696	20.8498028
N	-34.4850000	32.2820000	21.3720000
C	-35.1090000	33.4940000	21.4000000
N	-34.7130000	34.7520000	21.6760000
C	-33.3780000	34.6950000	21.9750000
N	-32.5590000	35.7920000	22.3310000
C	-31.3000000	35.1980000	22.5280000
N	-31.2130000	33.8910000	22.3580000
C	-32.5510000	33.5740000	22.0050000
C	-33.1530000	32.3240000	21.6880000
N	-32.4860000	31.1670000	21.6710000
C	-33.0320000	37.1550000	22.4220000
C	-32.1570000	38.0370000	23.3530000
C	-31.0650000	38.4950000	22.3790000
C	-31.8650000	38.6300000	21.0890000
C	-30.9600000	38.4470000	19.8430000
O	-32.8920000	37.6590000	21.0850000
O	-32.9530000	39.0490000	23.7420000
O	-30.5300000	39.6560000	22.8070000
H	-36.1735895	33.4194574	21.1410188
H	-30.4461174	35.8073356	22.8252673
H	-31.5064276	31.1360015	21.9118375
H	-32.9901055	30.3201998	21.4500109
H	-34.0901555	37.1345412	22.7135091
H	-31.7508928	37.4521691	24.1953370
H	-30.2724950	37.7379306	22.2835983
H	-32.3135554	39.6360881	21.0792394
H	-31.5590825	38.4684523	18.9209054
H	-30.1926026	39.2294200	19.8064246
H	-30.4722696	37.4607688	19.8816161
H	-33.0864660	39.0517928	24.6926482
H	-31.2482385	40.1634110	23.2130839
H	-30.5659375	44.9395296	22.5447471

H	-26.9643765	41.4640672	20.0802295
H	-26.9273514	43.0916864	16.4916595
H	-30.8439312	35.7223926	15.5197562
H	-33.9170611	38.8990475	13.7082020
H	-37.2001464	41.4516311	18.3286882

6WH5

C	22.0370000	7.0280000	17.7290000
C	21.8790000	5.5210000	17.5430000
O	22.7270000	4.8590000	16.9400000
H	21.7572944	7.3528066	18.7422161
N	20.7810000	4.9830000	18.0690000
C	20.4750000	3.5720000	17.8870000
C	19.9070000	3.3180000	16.4910000
O	19.3910000	4.2180000	15.8160000
C	19.4690000	3.0820000	18.9260000
C	20.0070000	3.0210000	20.3230000
C	21.1170000	2.2490000	20.6240000
C	19.3880000	3.7250000	21.3430000
C	21.6040000	2.1850000	21.9210000
C	19.8670000	3.6670000	22.6370000
C	20.9720000	2.8940000	22.9300000
H	20.0384050	5.5872997	18.3951309
H	21.4308935	3.0298677	17.9720308
H	18.5719232	3.7204971	18.8811293
H	19.1319926	2.0775918	18.6194885
H	21.6217835	1.6905617	19.8305600
H	18.5120384	4.3405089	21.1195752
H	22.4830179	1.5718126	22.1410987
H	19.3757515	4.2298789	23.4391613
H	21.3461797	2.8357105	23.9549542
N	19.9920000	2.0570000	16.0780000
C	19.5140000	1.6070000	14.7750000
H	20.5232713	1.4021109	16.6365439
H	19.0111723	2.4609934	14.3039845
Co	22.8860000	0.4310000	24.6950000
N	24.6720000	0.9080000	24.6080000
N	23.1430000	-0.7980000	23.2430000
N	21.0970000	0.0610000	24.9950000

N	22.8120000	1.7810000	26.0310000
C	25.0930000	2.1720000	25.2490000
C	24.9150000	3.3230000	24.2200000
C	26.6060000	1.8470000	25.6530000
C	27.4940000	3.1130000	25.8590000
C	26.6380000	0.9360000	26.9160000
C	26.9910000	0.9290000	24.4680000
C	27.8180000	1.5130000	23.2600000
C	25.6760000	0.3560000	23.9860000
C	25.5560000	-0.7510000	23.0510000
C	26.8580000	-1.4920000	22.6180000
C	24.3330000	-1.2070000	22.6430000
C	24.0670000	-2.4200000	21.7400000
C	24.9170000	-2.5020000	20.4750000
C	24.1650000	-3.7150000	22.6230000
C	22.5430000	-2.2120000	21.4390000
C	22.2720000	-1.2950000	20.1940000
C	22.1240000	-1.4340000	22.6680000
C	20.8370000	-1.4790000	23.1360000
C	20.3890000	-0.8630000	24.2820000
C	18.9140000	-0.8480000	24.6050000
C	18.5280000	-2.3330000	24.9150000
C	18.0460000	-0.4360000	23.3770000
C	18.8780000	-0.0110000	25.9100000
C	17.6870000	1.0390000	25.9260000
C	20.2340000	0.6660000	25.9150000
C	20.5880000	1.5050000	26.9800000
C	19.7060000	1.6300000	28.2320000
C	21.9400000	1.9870000	26.9660000
C	22.3670000	3.3440000	27.6180000
C	22.4300000	2.8530000	29.1260000
C	21.4360000	4.5120000	27.5930000
C	21.3450000	5.0260000	26.1370000
C	20.0930000	5.8850000	25.9420000
O	19.0200000	5.3540000	25.6800000
N	20.2920000	7.1850000	26.0390000
C	23.8030000	3.6180000	27.1160000
C	24.9390000	4.0300000	28.1040000
C	24.1130000	2.2860000	26.4660000

C	19.1060000	8.0370000	25.8120000
C	18.8550000	8.9900000	26.9790000
C	18.1670000	8.2450000	28.1340000
O	20.1580000	9.4500000	27.4400000
O	21.6310000	11.3930000	28.1200000
O	21.1220000	10.9060000	25.6200000
P	20.6900000	10.9370000	27.0860000
O	19.3070000	11.8240000	27.1130000
C	19.0350000	12.6060000	28.2370000
H	25.2180746	4.2904379	24.6418220
H	25.5038467	3.1322343	23.3144968
H	23.8558691	3.3898299	23.9299203
H	27.2199758	3.6401923	26.7768985
H	27.4253609	3.8062696	25.0111382
H	28.5486883	2.8126603	25.9601339
H	26.3765780	1.4792105	27.8332258
H	25.9673329	0.0708835	26.8083608
H	27.5951592	0.0892051	24.8528604
H	28.0499505	0.7259549	22.5352148
H	27.2722244	2.3082119	22.7347955
H	27.2226932	-1.1721515	21.6308623
H	27.6583556	-1.3152818	23.3461604
H	26.6949013	-2.5744440	22.5837807
H	25.0304531	-1.5198684	19.9961845
H	25.9178196	-2.9055798	20.6656370
H	24.4373837	-3.1789284	19.7511878
H	25.1803469	-3.8671589	23.0142224
H	23.4731446	-3.6518166	23.4757206
H	22.0021076	-3.1629921	21.3390306
H	22.5544080	-1.7977567	19.2586089
H	22.8450442	-0.3605503	20.2793017
H	20.1333241	-2.1014626	22.5847418
H	17.4794391	-2.3890572	25.2487287
H	18.6301630	-2.9694840	24.0244550
H	19.1621304	-2.7536946	25.7105215
H	18.3063105	0.5667899	23.0049403
H	18.1935093	-1.1545366	22.5612882
H	16.9822433	-0.4436296	23.6516929
H	18.8077439	-0.6783651	26.7838194

H	17.6449463	1.6148123	26.8541728
H	16.7269251	0.5171765	25.8034143
H	20.2948988	1.3651908	29.1169131
H	19.2838622	2.6346385	28.3828688
H	18.8738605	0.9225058	28.1974694
H	22.9457744	1.8847056	29.2340598
H	22.9665638	3.5792537	29.7563650
H	21.4276445	2.7676459	29.5502491
H	20.4316490	4.2359174	27.9400153
H	21.8166367	5.2958510	28.2669625
H	22.2427562	5.5944161	25.8547629
H	21.2413307	4.1687302	25.4597415
H	21.1467199	7.6486274	26.3239118
H	23.7429007	4.3842328	26.3310212
H	25.2414834	3.1984952	28.7550895
H	25.8044666	4.3855548	27.5375703
H	19.3144682	8.6498493	24.9196932
H	18.2572109	7.3740295	25.6191414
H	18.2533697	9.8485132	26.6464381
H	18.7784931	7.3804706	28.4363258
H	17.1742160	7.8850613	27.8236898
H	18.0551311	8.9080638	29.0039973
H	19.9565838	12.8122871	28.8049879
H	24.4722188	1.5860922	27.2317203
H	18.8013821	0.7746066	14.8806351
H	20.3501903	1.2883581	14.1331909
H	23.0808253	7.2901523	17.5245055
H	21.3925020	7.5546789	17.0076434
H	18.5849017	13.5598118	27.9120527
H	18.3135940	12.1089988	28.9167076
H	24.6211614	4.8641896	28.7477646
H	27.6623152	0.5550144	27.0508789
H	23.8948030	-4.5922365	22.0157407
H	21.2044671	-1.0365888	20.1439403
H	28.7679991	1.9343246	23.6181255
H	17.8116897	1.7533221	25.1010314

7RUU

C	7.27800000	-38.27500000	-51.06900000

C	7.52700000	-38.20300000	-52.56800000
O	7.50300000	-37.12800000	-53.17800000
N	7.78200000	-39.36700000	-53.15900000
C	7.83500000	-39.46900000	-54.60900000
C	6.45000000	-39.21300000	-55.19200000
O	5.43200000	-39.56700000	-54.59100000
C	8.33500000	-40.85400000	-55.03400000
C	9.78600000	-41.11200000	-54.70600000
C	10.78300000	-40.27500000	-55.19200000
C	10.15100000	-42.18800000	-53.91000000
C	12.11900000	-40.50800000	-54.89000000
C	11.48900000	-42.42800000	-53.60300000
C	12.46800000	-41.58700000	-54.09100000
N	6.41200000	-38.59400000	-56.36600000
C	5.17000000	-38.42100000	-57.10900000
N	20.74400000	-40.86400000	-62.98400000
C	20.18600000	-41.76900000	-62.11900000
N	19.98700000	-41.80200000	-60.77900000
C	20.48800000	-40.62300000	-60.27000000
N	20.48800000	-40.23200000	-58.90200000
C	21.10600000	-38.96800000	-58.94000000
N	21.49600000	-38.51200000	-60.11700000
C	21.09500000	-39.57600000	-60.96800000
C	21.22300000	-39.71900000	-62.37500000
N	21.78800000	-38.78400000	-63.15300000
C	19.94100000	-41.01200000	-57.81000000
C	20.77400000	-40.79000000	-56.50000000
C	19.99200000	-39.60700000	-55.86700000
C	18.54800000	-39.99700000	-56.24300000
C	17.57600000	-38.83000000	-56.12100000
O	18.60300000	-40.51100000	-57.56600000
O	20.63700000	-41.92100000	-55.78500000
O	20.23900000	-39.55200000	-54.54000000
Co	15.63300000	-39.66100000	-55.59000000
N	15.70900000	-38.48900000	-54.14700000
N	14.76300000	-38.29600000	-56.62400000
N	15.43000000	-41.03400000	-56.80000000
N	16.43700000	-40.84400000	-54.32300000
C	15.86400000	-39.06600000	-52.78800000

C	14.52200000	-39.59900000	-52.21400000
C	16.45600000	-37.86700000	-51.93800000
C	16.14100000	-38.05200000	-50.42100000
C	17.97900000	-37.74600000	-52.21700000
C	15.74200000	-36.68400000	-52.64800000
C	15.63200000	-37.18600000	-54.08100000
C	15.20200000	-36.36100000	-55.19800000
C	15.73300000	-34.89600000	-55.14300000
C	14.67600000	-36.92400000	-56.35200000
C	14.23100000	-36.21200000	-57.64800000
C	13.32100000	-34.98900000	-57.50600000
C	15.51500000	-35.96900000	-58.53600000
C	13.47200000	-37.37800000	-58.37000000
C	11.94800000	-37.41700000	-57.98400000
C	14.14300000	-38.59300000	-57.76500000
C	14.39200000	-39.70900000	-58.53300000
C	14.96300000	-40.87100000	-58.06800000
C	15.08600000	-42.12200000	-58.91000000
C	16.31500000	-41.83400000	-59.84100000
C	13.86200000	-42.44900000	-59.80800000
C	15.41800000	-43.18500000	-57.83800000
C	14.24600000	-44.22100000	-57.59800000
C	15.69500000	-42.37900000	-56.59500000
C	16.44800000	-42.95100000	-55.57600000
C	17.16500000	-44.28700000	-55.84500000
C	16.68600000	-42.11000000	-54.42800000
C	16.93300000	-42.62700000	-52.97200000
C	18.50400000	-42.82100000	-53.15000000
C	16.33400000	-43.91000000	-52.47500000
C	14.78700000	-43.89000000	-52.62200000
C	14.21700000	-45.17800000	-52.01800000
O	14.35500000	-46.24800000	-52.59200000
N	13.57700000	-45.03900000	-50.88000000
C	16.79100000	-41.40600000	-52.04700000
C	17.85500000	-41.25000000	-50.92200000
C	16.83900000	-40.26600000	-53.03500000
C	13.01600000	-46.28200000	-50.32800000
C	11.49300000	-46.13900000	-50.40800000
C	10.74400000	-47.40000000	-49.94800000

O	11.16800000	-45.06200000	-49.47800000
O	12.66376312	-44.91446775	-47.41870669
O	10.66676433	-46.57230243	-47.47215793
P	11.29921278	-45.23615131	-47.86882676
O	10.25222077	-44.07686257	-47.37284760
C	8.88500293	-44.26844321	-47.59133840
H	7.88410570	-39.04910040	-50.57540290
H	7.69159740	-41.62080900	-54.57493170
H	8.18496420	-40.94049330	-56.12385270
H	10.51795600	-39.42051350	-55.82275510
H	9.40330370	-42.83882570	-53.44253200
H	12.89091530	-39.84066800	-55.28681850
H	11.72203590	-43.26742180	-52.94256110
H	13.51999560	-41.80457980	-53.90112890
H	8.52032830	-38.68299030	-54.96953780
H	4.36634730	-38.83266380	-56.48604330
H	19.82046850	-42.66595360	-62.63540720
H	21.25863230	-38.40681420	-58.01858550
H	22.17191210	-37.94551670	-62.74290450
H	19.91439670	-42.06447480	-58.12118190
H	21.82513710	-40.53346980	-56.73441770
H	20.26494990	-38.66588120	-56.38006260
H	18.29205040	-40.80682040	-55.55083570
H	20.86676970	-41.74827050	-54.86271930
H	19.85251850	-38.76555810	-54.14276580
H	14.63343060	-39.95378100	-51.18545700
H	13.75501820	-38.80988320	-52.22233440
H	14.14330440	-40.41575830	-52.83283470
H	16.65527050	-37.27863600	-49.82964300
H	16.48187440	-39.02815320	-50.06238920
H	15.06579760	-37.96039750	-50.22240220
H	18.54841870	-38.57049940	-51.76762710
H	18.17594830	-37.71106350	-53.29898550
H	16.27933120	-35.72947800	-52.55545770
H	16.81417720	-34.92453250	-54.93945870
H	15.25451070	-34.29919440	-54.35082230
H	15.58506230	-34.37153870	-56.09204720
H	12.51936150	-35.16202360	-56.77733150
H	13.87409580	-34.09611090	-57.18165800

H	12.86836760	-34.74756790	-58.47895540
H	16.21167680	-35.26070500	-58.06431450
H	16.04485710	-36.91788420	-58.70341180
H	13.58626240	-37.34145320	-59.46167480
H	11.82715030	-37.40768970	-56.89038630
H	11.40157460	-36.56626460	-58.41070960
H	13.98322910	-39.71971720	-59.54308830
H	17.20267030	-41.56836260	-59.24977790
H	16.54806080	-42.72445060	-60.44507480
H	16.09165790	-41.00055920	-60.52242930
H	12.92759330	-42.51994270	-59.23591450
H	14.03279340	-43.40220800	-60.32715360
H	13.73563320	-41.67614740	-60.57997460
H	16.31986330	-43.73963180	-58.13148230
H	14.49711960	-44.89997940	-56.77171700
H	14.06572220	-44.82305300	-58.49776670
H	17.48210500	-44.78240100	-54.92104350
H	16.52772040	-45.00034140	-56.37635580
H	18.06309960	-44.08688970	-56.45152510
H	18.96599510	-43.21674450	-52.23229760
H	18.99815830	-41.86985450	-53.39835670
H	18.73310100	-43.52868690	-53.95538300
H	16.61060760	-44.02800230	-51.41400160
H	16.72002460	-44.79097750	-53.00580370
H	14.36135970	-43.00207340	-52.12915250
H	14.51779460	-43.86792420	-53.68754810
H	15.80097080	-41.45831010	-51.57864420
H	17.55682720	-40.46869870	-50.21395100
H	18.84352340	-40.98736640	-51.32122510
H	17.86559230	-39.89900830	-53.17491370
H	13.30901810	-46.40073240	-49.27394080
H	13.38493240	-47.11252710	-50.93547820
H	11.17970350	-45.83840080	-51.42264810
H	11.08040830	-47.68046570	-48.93850460
H	9.66947380	-47.17743440	-49.88717960
H	10.91550920	-48.24255890	-50.63716730
H	8.59572282	-45.31405610	-47.38159284
H	21.86764880	-38.95824690	-64.14460620
H	6.21635510	-38.51318930	-50.89781990

H	7.48836220	-37.29158620	-50.63281360
H	4.96602920	-37.35554580	-57.29743390
H	5.20069080	-38.95781970	-58.07054930
H	8.32217243	-43.58913648	-46.92815274
H	8.59041397	-44.02844418	-48.63722159
H	7.27190530	-38.24482220	-56.76654850
H	7.66483120	-40.22623380	-52.63481960
H	14.71944540	-36.52457620	-52.26445290
H	11.49151100	-38.34235950	-58.36579570
H	18.36053530	-36.80931470	-51.78150840
H	17.95520440	-42.17994320	-50.34247010
H	13.32035060	-43.69834030	-57.31955100
H	13.23599510	-44.16333010	-50.50204280
H	15.21085720	-35.55774080	-59.50996880
H	17.81159940	-38.15657780	-55.29172570
H	17.43198600	-38.29696380	-57.06891120

7RUV

C	6.65839530	-33.86499970	-31.46672510
C	7.82516480	-34.80099130	-31.60060320
O	8.53045160	-34.81640590	-32.62311540
N	8.06654710	-35.61383750	-30.56120660
C	9.18729280	-36.53375450	-30.56536630
C	8.86748460	-37.84051530	-31.32372450
O	8.91797310	-38.93268090	-30.78676670
C	9.66487220	-36.85013320	-29.15245610
C	10.28482120	-35.66948150	-28.46079980
C	11.64879300	-35.38437850	-28.59672190
C	9.49594250	-34.81825750	-27.67746820
C	12.21379720	-34.27931170	-27.96034300
C	10.05574750	-33.71300280	-27.04383890
C	11.41376050	-33.43839710	-27.18629180
N	8.56089240	-37.64474770	-32.62672960
C	8.16544110	-38.72013820	-33.49230720
Co	13.43691560	-31.59936360	-29.50452600
N	12.65241550	-32.06958610	-31.13670530
N	14.81356200	-32.89938760	-29.80604750
N	13.88049610	-31.27705560	-27.65937360
N	11.97213740	-30.44200460	-29.37801720

C	11.26972640	-31.59284440	-31.36502470
C	10.31379490	-32.57518410	-30.68759550
C	11.15914810	-31.58741350	-32.92879350
C	9.75113620	-31.82070940	-33.46542140
C	11.71015270	-30.28641670	-33.52516950
C	12.15670670	-32.72229570	-33.31885370
C	11.57769300	-34.10691520	-33.61335190
C	13.12449610	-32.71285460	-32.15891720
C	14.42144960	-33.34314200	-32.17915410
C	14.92735940	-33.86326960	-33.50095600
C	15.15484990	-33.47036470	-31.02285110
C	16.51718940	-34.16513180	-30.88601630
C	16.72237100	-35.47111900	-31.65049550
C	17.59977470	-33.14250710	-31.27258120
C	16.57271800	-34.38272840	-29.35572270
C	15.99898040	-35.71019990	-28.84995820
C	15.69693480	-33.27039610	-28.86640700
C	15.77173310	-32.77768060	-27.57528370
C	14.90835450	-31.84404930	-27.01560450
C	15.05492060	-31.36612900	-25.58465110
C	16.23118220	-30.38332570	-25.50255610
C	15.29607020	-32.52252330	-24.61343050
C	13.71717480	-30.60905620	-25.38138670
C	12.70015890	-31.30384410	-24.46994590
C	13.18754440	-30.45008390	-26.79373590
C	12.11694960	-29.64420340	-27.10938820
C	11.54706770	-28.74595050	-26.04353650
C	11.50599020	-29.69932990	-28.41717230
C	10.29305270	-28.88401160	-28.87885310
C	10.74705380	-27.43687440	-29.11502710
C	9.09105320	-28.97087990	-27.92327860
C	8.67103850	-30.41101760	-27.64426560
C	8.18721950	-30.70564260	-26.23496290
O	8.80376670	-30.32619330	-25.23974340
N	7.10183280	-31.49134160	-26.21718020
C	9.93816120	-29.59495260	-30.22044640
C	9.26012880	-28.72137090	-31.25977500
C	11.25474290	-30.23461130	-30.63802060
C	6.62034820	-32.22051280	-25.07576430

C	6.35381780	-33.67732620	-25.46203300
C	7.18600304	-34.65419063	-24.65909135
O	4.98564256	-33.98635284	-25.24016686
O	4.23090911	-35.00278575	-27.50980034
O	3.59012939	-32.55241809	-26.83548389
P	3.86563012	-33.99730325	-26.44964894
O	2.61029240	-34.59100258	-25.61329864
C	2.02844112	-33.80046038	-24.60398942
N	20.94836380	-25.79470920	-27.41360290
C	19.69553470	-25.36424410	-27.25847620
N	18.56262010	-26.01406710	-27.51533250
C	18.80527330	-27.24315320	-27.97370760
N	17.89236950	-28.19249040	-28.34671900
C	18.61575150	-29.29215480	-28.74971310
N	19.90898890	-29.11629070	-28.67137960
C	20.05061550	-27.83880110	-28.18845230
C	21.16802560	-27.02993470	-27.88096730
N	22.42951730	-27.46144880	-28.04255090
C	16.46146530	-28.01391360	-28.25166490
C	15.91936180	-26.93142600	-29.20766590
C	15.19775590	-27.76252140	-30.27280450
C	14.75970010	-28.99241900	-29.46321650
C	14.49072530	-30.19695190	-30.30884040
O	15.84735070	-29.22109080	-28.57235930
O	14.94104380	-26.14232060	-28.58346950
O	14.15100870	-27.09401670	-30.89293560
H	5.74778930	-34.41635550	-31.18790740
H	10.38142010	-37.67972800	-29.22783930
H	8.81250100	-37.23536130	-28.57271120
H	12.27855790	-36.04544320	-29.20127380
H	8.42468290	-35.02407770	-27.59480920
H	13.28364250	-34.08310070	-28.05158430
H	9.42654620	-33.06221550	-26.43325770
H	11.83943250	-32.56375450	-26.69977820
H	10.00462950	-36.04473450	-31.12632080
H	8.25183780	-39.65927990	-32.92956340
H	10.44964550	-33.59010860	-31.07637380
H	10.49912190	-32.61017860	-29.60531390
H	9.26453280	-32.29850980	-30.84841880

H	9.06837470	-31.02503290	-33.13330270
H	9.76398990	-31.79405820	-34.56655860
H	9.31972690	-32.78541750	-33.16476220
H	11.07896940	-29.42017630	-33.28379250
H	12.73508300	-30.06914240	-33.18314970
H	12.70080200	-32.40192090	-34.22318400
H	10.91843690	-34.47779990	-32.81891840
H	12.38149960	-34.84029050	-33.77011410
H	16.00046540	-33.67445210	-33.62742550
H	14.42266630	-33.36414500	-34.33663580
H	14.76491810	-34.94482030	-33.62801050
H	17.58638470	-36.00478790	-31.22545180
H	15.84877620	-36.13383520	-31.58138840
H	16.94421610	-35.30802750	-32.71090510
H	17.52184800	-32.23111270	-30.65887850
H	17.51463420	-32.84431870	-32.32757660
H	17.60337970	-34.27104740	-28.98240570
H	14.97050900	-35.86251620	-29.21265080
H	16.60931330	-36.56520140	-29.17165580
H	16.55864800	-33.18404610	-26.94013690
H	16.34218260	-30.02233140	-24.46837000
H	16.06255080	-29.52273680	-26.16026980
H	17.17573350	-30.86013500	-25.80556400
H	15.26518670	-32.16251840	-23.57490690
H	14.55317060	-33.32431140	-24.72296490
H	16.29194100	-32.96196020	-24.77237040
H	13.92623540	-29.61404410	-24.95830940
H	13.04663700	-31.29658940	-23.42678070
H	11.72083300	-30.80889600	-24.50374860
H	12.27979380	-28.55476070	-25.25126760
H	10.64325820	-29.18312070	-25.58408020
H	11.26842700	-27.76725980	-26.45170440
H	11.56005870	-27.38992270	-29.85730500
H	11.11525340	-26.96986590	-28.19200290
H	9.90978750	-26.82597750	-29.48235060
H	9.29728210	-28.45203100	-26.97998430
H	8.25779550	-28.41975640	-28.39090750
H	9.54680190	-31.07579870	-27.74945340
H	7.93709470	-30.78528030	-28.37140740

H	9.25124550	-30.41168430	-29.96567880
H	9.93086160	-27.94385910	-31.65570260
H	8.90661510	-29.32285850	-32.10811950
H	11.85401100	-29.51507960	-31.22018240
H	7.37038700	-32.15553980	-24.27156970
H	5.67983230	-31.78561020	-24.69133270
H	6.59160429	-33.80194926	-26.53447337
H	6.95036896	-34.56242342	-23.58767844
H	8.26217248	-34.47316447	-24.80260004
H	6.95264549	-35.68027533	-24.97475330
H	1.85315727	-32.77100687	-24.95812665
H	19.59113750	-24.34334800	-26.87230080
H	18.10400090	-30.19611780	-29.07757670
H	23.19464220	-26.84602520	-27.80781270
H	16.22931890	-27.70014590	-27.21643500
H	15.92239530	-28.08192250	-31.04130000
H	13.87019810	-28.69048770	-28.88066380
H	15.44430650	-30.64168250	-30.63398200
H	13.93300770	-29.87301780	-31.19915170
H	13.82963780	-26.45199570	-30.24093730
H	10.97117340	-34.07807420	-34.52879330
H	18.60169090	-33.57238960	-31.11877290
H	11.73987660	-30.37136690	-34.62213270
H	12.54073200	-32.34984580	-24.76248040
H	6.82282910	-33.13954850	-30.65343800
H	6.50465880	-33.35197830	-32.42333870
H	8.81228000	-38.78061950	-34.38333210
H	7.12170110	-38.60918830	-33.83235450
H	1.06822209	-34.26018910	-24.32862770
H	2.67523995	-33.76092749	-23.71073346
H	8.37677980	-28.22300910	-30.83245280
H	15.96588780	-35.71298980	-27.75088220
H	22.60239910	-28.39188180	-28.39196340
H	16.73626870	-26.31567390	-29.61555090
H	6.71231320	-31.77489520	-27.13965510
H	15.38433960	-25.45584770	-28.07207280
H	8.56841000	-36.67932100	-32.96345740
H	7.44911590	-35.54973350	-29.72441110

8DYJ

C	25.55839790	3.47690940	25.79433980
C	25.76605960	4.41742660	24.65766270
N	26.79425040	5.32018550	24.64037610
C	25.03411540	4.53612100	23.49210070
C	26.69047370	5.95572370	23.49173980
N	25.63160130	5.53218670	22.75983380
Co	25.21015770	6.06264450	21.03734490
N	26.93802400	5.77500450	20.37635730
N	25.64462730	7.89696120	21.42145100
N	23.35135900	6.19349280	21.46960630
N	24.85868090	4.41472360	20.18733390
C	27.24385540	4.37338590	19.98279530
C	27.48762470	3.53007810	21.23062620
C	28.51513940	4.54021520	19.08618410
C	29.44914580	3.33554680	19.09306220
C	28.14302060	4.85911110	17.63715270
C	29.15342960	5.83472050	19.68190850
C	30.23974490	5.65512100	20.74543870
C	27.95444970	6.56865880	20.23649630
C	27.95132790	7.96703720	20.60601800
C	29.19257820	8.76153790	20.29691600
C	26.86190060	8.54012070	21.21756340
C	26.75654250	9.99067510	21.71257880
C	27.97810330	10.52768520	22.45948340
C	26.42659240	10.91372600	20.52554130
C	25.51192920	9.90399880	22.63260350
C	25.78394610	9.67344180	24.12393410
C	24.81184290	8.69209650	22.10380450
C	23.46672770	8.44067820	22.35654400
C	22.78650840	7.28385900	22.02394390
C	21.29555680	7.09364130	22.20276740
C	20.61015420	7.55756560	20.90366290
C	20.70328060	7.83134850	23.39558740
C	21.23265310	5.55332560	22.28466710
C	21.34371430	4.98394910	23.70090890
C	22.44926850	5.14624180	21.48277490
C	22.63273480	3.89764670	20.92932620
C	21.54540490	2.86376480	21.04129350

C	23.85221760	3.58265390	20.22526070
C	24.09222700	2.37309090	19.32901140
C	23.23827110	2.55774680	18.06312230
C	23.81875740	1.00328550	19.96683570
C	24.34218260	0.82036840	21.39213300
C	24.48625960	-0.67511200	21.63782030
O	23.50905130	-1.40759800	21.69389120
N	25.76602540	-1.08041870	21.66794630
C	25.61999320	2.48600650	19.02120850
C	26.02675700	1.97459740	17.65071670
C	25.93535350	3.95071730	19.29326380
C	26.15962500	-2.45323390	21.49517800
C	27.58720430	-2.51578160	20.95640300
C	28.03688390	-3.94065530	20.70488250
O	27.68001650	-1.79527070	19.73427170
O	27.77362380	0.52391480	20.81971180
O	28.43041170	0.16618870	18.29362160
P	28.39593140	-0.30992950	19.71156100
O	29.91006040	-0.65906810	20.24257640
C	30.80467870	-1.24812850	19.33183760
O	23.15060040	10.53055070	18.65929370
C	23.45188650	9.18604120	18.88311890
C	23.26529680	8.32215180	17.65570290
O	23.94410550	8.93099130	16.56036870
C	23.86683510	6.92822710	17.80933150
O	22.88842430	6.02227910	18.23606130
C	24.37007670	6.63096130	16.38859980
O	23.30130410	6.20534070	15.60645680
C	24.79337020	8.02513280	15.92328220
N	26.17000760	8.33147360	16.30024180
C	26.60395900	9.26755540	17.20911740
N	27.90419570	9.31180880	17.33725290
C	28.36250480	8.36045850	16.45967280
C	29.64895500	7.88373130	16.12798330
N	30.76412170	8.33808770	16.74367760
N	29.75541320	6.92545720	15.20503340
C	28.64751080	6.43571770	14.63865050
N	27.38576630	6.77838360	14.87623170
C	27.30054150	7.74137220	15.78997970

H	26.44970300	2.84834130	25.94630080
H	24.70105820	2.81384170	25.61132730
H	24.17849730	3.97310480	23.12951290
H	27.37692480	6.72579820	23.13895880
H	28.26836250	3.97259360	21.86127430
H	26.58076140	3.47333520	21.84393650
H	27.77708490	2.49936050	20.97166040
H	29.79378590	3.06386640	20.09825540
H	30.33618810	3.55391770	18.47707470
H	28.97052650	2.43784940	18.67234000
H	27.48375840	5.73753590	17.56098110
H	27.65260100	4.01036070	17.14455050
H	29.59206360	6.41929040	18.85657060
H	30.62951030	6.62631270	21.08062150
H	29.87219970	5.11905360	21.62989900
H	28.94747220	9.76850180	19.94433090
H	29.86084860	8.84820230	21.16744320
H	29.76021920	8.28822270	19.48810790
H	27.69222600	11.43550770	23.01189570
H	28.37843670	9.80295120	23.18246190
H	28.78538810	10.81533070	21.77773700
H	25.44225200	10.68722450	20.08893890
H	27.18227460	10.84031710	19.72947980
H	24.88426290	10.80209980	22.51984870
H	24.83420620	9.54925710	24.66395680
H	26.31277020	10.52625890	24.57009330
H	22.90907140	9.22767470	22.86327760
H	19.53011790	7.35190110	20.96023400
H	20.74128320	8.63967310	20.75202460
H	21.01809840	7.03253900	20.02546450
H	21.25121620	7.62750140	24.32580740
H	19.65363910	7.53415480	23.53997230
H	20.70916120	8.91844930	23.22647170
H	20.30593610	5.19116290	21.81698240
H	22.26144660	5.33377280	24.19861910
H	21.38307810	3.88595820	23.67338250
H	20.60710300	3.29359610	21.40581600
H	21.82492420	2.05098970	21.72818640
H	21.32913620	2.39480270	20.07360580

H	22.16642380	2.62612550	18.29654100
H	23.37845720	1.69952520	17.39230430
H	23.51734840	3.46426400	17.50445910
H	22.75650610	0.72552300	19.92627360
H	24.33103340	0.27617870	19.31314430
H	23.63945080	1.23578540	22.13084750
H	25.30870920	1.32816290	21.52724210
H	26.15749250	1.87571270	19.76419080
H	27.11251550	1.82523220	17.59126930
H	25.68340390	2.63225650	16.83622000
H	25.83492020	4.54062900	18.36524550
H	26.11010510	-3.01556900	22.44536010
H	25.46583270	-2.95207330	20.79568820
H	28.25965490	-2.05141140	21.70227440
H	29.08349920	-3.95170660	20.36960660
H	27.95545240	-4.55119620	21.61686960
H	27.41852230	-4.39744600	19.91701800
H	30.55527380	-2.30846560	19.14265770
H	22.80388580	8.81228020	19.68742020
H	24.49446540	9.05775870	19.23716440
H	22.19659020	8.23060500	17.39674710
H	24.72384930	6.96014580	18.51083890
H	23.28341550	5.38955150	18.84887160
H	25.22191590	5.92637720	16.35471700
H	22.70911210	5.71758440	16.19841460
H	24.69858120	8.10983920	14.83012530
H	25.89795660	9.90553270	17.73448220
H	30.70969480	9.19948570	17.26859140
H	28.80447850	5.64979500	13.89146340
H	23.49702250	10.75549130	17.78579410
H	31.65679540	8.05330530	16.36280700
H	31.08211150	5.08656970	20.32903230
H	20.47784620	5.27343850	24.31275110
H	30.79197200	-0.71879960	18.36502440
H	31.81408300	-1.20000210	19.76782340
H	26.37704430	8.76197830	24.29193900
H	25.38163810	4.02171290	26.73552200
H	26.39365260	11.95814520	20.87240900
H	25.61459760	0.96851800	17.48197160

H	29.05456640	5.08476430	17.06561590
H	26.50571390	-0.37446290	21.48621780
H	27.48142947	5.46677851	25.35192049

Cartesian coordinates of complexes 2 to 10, 12 to 15 and 17 to 20

2

Co	0.3871474	-1.9376176	0.0024905
N	0.2760374	-1.1976421	-1.7015080
N	-0.5305118	-3.4975409	-0.6501379
N	0.3643323	-2.4554290	1.8431286
N	1.2164998	-0.3238622	0.4372456
C	0.4805159	0.2802744	-1.7884740
C	-0.8417477	0.9609337	-1.3951118
C	0.8751502	0.5022186	-3.3017010
C	0.4571818	1.8739681	-3.8474821
C	2.3901422	0.3077389	-3.5315597
C	0.1501534	-0.7095551	-4.0001019
C	-1.2201562	-0.4446719	-4.6528381
C	0.0646080	-1.7363993	-2.8819168
C	-0.2663495	-3.1271525	-3.0721601
C	-0.2253938	-3.6803927	-4.4825892
C	-0.6284346	-3.9080411	-1.9847472
C	-1.0878632	-5.3818715	-2.0310679
C	-2.0305182	-5.7927975	-3.1749320
C	0.1820022	-6.2717641	-2.0327490
C	-1.7778523	-5.5056611	-0.6365982
C	-3.2772903	-5.1455649	-0.6039397
C	-1.0015404	-4.4851634	0.1564075
C	-0.7918724	-4.5802589	1.5311308
C	-0.1063524	-3.6455465	2.3050365
C	0.2613291	-3.8620333	3.7691566
C	1.6347412	-4.5829046	3.7974119
C	-0.7646488	-4.6697422	4.5741055
C	0.4449908	-2.3859352	4.2220140
C	-0.8272866	-1.7330238	4.7988900
C	0.8319801	-1.6975645	2.9155383
C	1.4263069	-0.4467676	2.8444573
C	1.8225074	0.2392789	4.1360239

C	1.5833409	0.2313469	1.5755722
C	2.0588232	1.6852068	1.3851123
C	3.5534780	1.8559817	1.7173381
C	1.1551052	2.6709376	2.1939429
C	-0.3083229	2.1921430	2.1779758
C	-1.5162954	3.1134395	2.0290039
O	-2.6470897	2.5895253	2.0613528
N	-1.2981300	4.4384405	1.8348964
C	1.7719380	1.9281145	-0.1479919
C	2.7719807	2.8085310	-0.8929132
C	1.5745021	0.5235000	-0.7241060
C	-2.3828552	5.3124446	1.4115617
C	-2.4214116	5.4923877	-0.1207473
C	-3.6205292	6.3224808	-0.5675414
O	-1.2400846	6.1680659	-0.5720810
O	-0.1745889	4.0101802	-1.6053702
O	0.9828060	5.2663662	0.4370924
P	0.1625958	5.2966211	-0.8678774
O	0.8810800	6.3292261	-1.9345218
C	1.3423766	7.5843119	-1.4410506
H	1.8972370	7.4616678	-0.4851735
H	-3.5829345	7.3320969	-0.1087755
H	-3.6024590	6.4457641	-1.6677065
H	-4.5738416	5.8362454	-0.2765513
H	-2.4633484	4.4805248	-0.5866522
H	-3.3346774	4.8668693	1.7624339
H	-2.2548411	6.3112576	1.8810706
H	-0.3385052	4.7650726	1.5684967
H	-0.4525917	1.5156551	1.3136710
H	-0.5376759	1.5564650	3.0582789
H	1.5211808	2.7997493	3.2322061
H	1.2680583	3.6553188	1.6901221
H	3.8595295	2.9014987	1.5225588
H	3.7837778	1.6412265	2.7777719
H	4.1858798	1.1918484	1.0945524
H	2.3870525	3.0275353	-1.9063982
H	3.7773166	2.3475357	-0.9834474
H	0.8067195	2.4620579	-0.2240008
H	2.5320971	0.1086001	-1.1049565

H	-1.6453486	0.6676825	-2.0960874
H	-1.1526369	0.6010962	-0.3965511
H	-0.7430708	2.0656851	-1.4052025
H	0.8097414	1.9954280	-4.8928318
H	-0.6362394	2.0265155	-3.8365482
H	0.8608803	2.6997620	-3.2311763
H	2.9847063	1.1088244	-3.0538835
H	2.7487218	-0.6706500	-3.1473714
H	0.8157949	-1.1011492	-4.7997872
H	-1.6319178	-1.3731781	-5.0944127
H	-1.9588574	-0.0645243	-3.9224372
H	0.4287239	-3.0646630	-5.1265244
H	0.1809338	-4.7079943	-4.5085968
H	-1.2227403	-3.7077500	-4.9679314
H	-1.4945202	-5.9629769	-4.1250851
H	-2.5252863	-6.7508176	-2.9136795
H	-2.8196773	-5.0389488	-3.3569362
H	0.7828617	-6.1121876	-2.9494199
H	0.8281084	-6.0442692	-1.1608777
H	-1.6417918	-6.5265223	-0.2213225
H	-3.6404234	-5.1300622	0.4429292
H	-3.4519387	-4.1391267	-1.0358892
H	-0.7726407	-5.7310435	4.2538865
H	-0.5027669	-4.6590733	5.6514375
H	-1.7926389	-4.2749790	4.4628211
H	2.4026393	-3.9989826	3.2500763
H	1.9785277	-4.7137810	4.8443842
H	1.5633912	-5.5831505	3.3252165
H	1.2574181	-2.3141403	4.9728207
H	-1.6739768	-1.8160731	4.0869462
H	-1.1312886	-2.2049916	5.7534590
H	2.7228409	0.8637922	4.0157282
H	1.0203415	0.9103452	4.5093099
H	2.0396692	-0.4886260	4.9382010
H	2.8493015	3.7909247	-0.3882640
H	2.6071839	0.3406750	-4.6189246
H	-0.0995672	-7.3441726	-1.9881025
H	-3.8897635	-5.8784384	-1.1641648
H	-1.1268552	0.3000160	-5.4664663

H	-0.6640912	-0.6558385	4.9921312
H	-1.1774997	-5.4781723	2.0319437
H	2.0164295	8.0204272	-2.2066545
H	0.4946459	8.2851756	-1.2676326
C	2.0868656	-2.8447742	-0.3235116
H	2.0385010	-3.8304965	0.1811846
H	2.2072329	-2.9702497	-1.4179023
H	2.9097420	-2.2358968	0.0986541
N	-2.0371274	-0.5284631	1.4155633
H	-2.4586397	0.4113236	1.5521173
H	-2.7767927	-1.2213174	1.5864056
H	-1.3683219	-0.6309224	2.1881740

3

Co	0.3181719	-1.9018445	0.0089095
N	0.1753988	-1.1520416	-1.6963477
N	-0.6388544	-3.4477437	-0.6341359
N	0.2720717	-2.3978296	1.8584037
N	1.1592056	-0.2836307	0.4360032
C	0.4072121	0.3232071	-1.7840153
C	-0.8991163	1.0239069	-1.3732372
C	0.7886629	0.5325035	-3.3021897
C	0.3857107	1.9080421	-3.8484717
C	2.2979681	0.3080264	-3.5444202
C	0.0364937	-0.6693292	-3.9890176
C	-1.3389598	-0.3916928	-4.6252945
C	-0.0493539	-1.6944041	-2.8711328
C	-0.3815625	-3.0848469	-3.0575902
C	-0.3398573	-3.6427819	-4.4651185
C	-0.7273175	-3.8669681	-1.9676820
C	-1.1428112	-5.3520230	-1.9990409
C	-2.0582135	-5.8152659	-3.1439627
C	0.1653569	-6.1870768	-1.9723669
C	-1.8450966	-5.4769368	-0.6109986
C	-3.3528690	-5.1515308	-0.5965030
C	-1.0988774	-4.4332885	0.1792053
C	-0.8955255	-4.5168946	1.5548501
C	-0.1980101	-3.5862383	2.3214624
C	0.2009963	-3.8129390	3.7741363

C	1.5697873	-4.5456567	3.7510892
C	-0.8099658	-4.6174229	4.6002699
C	0.4094266	-2.3413698	4.2313311
C	-0.8408126	-1.6802917	4.8456994
C	0.7728596	-1.6488855	2.9215591
C	1.3847095	-0.4084858	2.8420882
C	1.8139815	0.2708090	4.1257213
C	1.5418862	0.2636835	1.5700031
C	2.0481157	1.7054092	1.3671520
C	3.5521236	1.8331352	1.6771273
C	1.1808515	2.7151837	2.1852032
C	-0.2930880	2.2682444	2.2003703
C	-1.4842994	3.2107116	2.0459177
O	-2.6241859	2.7051176	2.0868675
N	-1.2465058	4.5297141	1.8446101
C	1.7466362	1.9501013	-0.1631793
C	2.7543942	2.8104710	-0.9209366
C	1.5204461	0.5479958	-0.7355583
C	-2.3212900	5.4199929	1.4283762
C	-2.3775551	5.5876835	-0.1048062
C	-3.5674335	6.4356974	-0.5423660
O	-1.1901912	6.2376541	-0.5777290
O	-0.1765794	4.0503950	-1.5995852
O	1.0237370	5.3024113	0.4196101
P	0.1926661	5.3379102	-0.8788288
O	0.9217846	6.3444918	-1.9610853
C	1.4128557	7.5955976	-1.4848731
H	1.9748739	7.4711974	-0.5335752
H	-3.5059438	7.4480358	-0.0923643
H	-3.5616457	6.5499660	-1.6436040
H	-4.5252517	5.9688259	-0.2351056
H	-2.4443176	4.5727514	-0.5613157
H	-3.2765274	4.9958145	1.7957713
H	-2.1673763	6.4198415	1.8875995
H	-0.2838267	4.8393789	1.5654884
H	-0.4655021	1.5711487	1.3578269
H	-0.5235291	1.6610180	3.1005032
H	1.5694160	2.8434625	3.2152355
H	1.3055882	3.6933589	1.6715292

H	3.8829948	2.8691518	1.4728636
H	3.7917312	1.6175194	2.7352166
H	4.1554315	1.1478934	1.0490397
H	2.3611493	3.0362566	-1.9295868
H	3.7488878	2.3298496	-1.0227581
H	0.7897639	2.5003051	-0.2283041
H	2.4642299	0.1083642	-1.1202826
H	-1.7157884	0.7452444	-2.0649480
H	-1.2050891	0.6747929	-0.3684025
H	-0.7801163	2.1270812	-1.3876065
H	0.7297398	2.0190889	-4.8976631
H	-0.7051498	2.0776943	-3.8270894
H	0.8094911	2.7295971	-3.2401064
H	2.9098864	1.1040699	-3.0805364
H	2.6443008	-0.6723120	-3.1550413
H	0.6878291	-1.0731149	-4.7937207
H	-1.7657155	-1.3174165	-5.0588280
H	-2.0646549	0.0000434	-3.8878971
H	0.2868762	-3.0111060	-5.1198658
H	0.1008988	-4.6558805	-4.4871722
H	-1.3424336	-3.7045672	-4.9359403
H	-1.5065762	-5.9830174	-4.0853214
H	-2.5187339	-6.7864637	-2.8694866
H	-2.8740057	-5.0957716	-3.3470276
H	0.7492238	-6.0406219	-2.9017745
H	0.8162767	-5.8899172	-1.1255185
H	-1.6876951	-6.4886164	-0.1819954
H	-3.7280893	-5.1317136	0.4460288
H	-3.5485213	-4.1561239	-1.0447665
H	-0.8348386	-5.6763555	4.2737127
H	-0.5168477	-4.6167024	5.6693788
H	-1.8374930	-4.2134193	4.5218887
H	2.3192436	-3.9785755	3.1628043
H	1.9514838	-4.6694948	4.7854073
H	1.4695620	-5.5490730	3.2916947
H	1.2427133	-2.2819594	4.9596444
H	-1.7099978	-1.7577926	4.1606482
H	-1.1187285	-2.1527886	5.8078114
H	2.7254241	0.8753214	3.9896932

H	1.0310466	0.9595434	4.5073147
H	2.0269825	-0.4599017	4.9261652
H	2.8559261	3.7914071	-0.4177753
H	2.5040138	0.3299235	-4.6341101
H	-0.0725205	-7.2668628	-1.8804837
H	-3.9397644	-5.9073711	-1.1531038
H	-1.2458253	0.3490460	-5.4423025
H	-0.6648980	-0.6047182	5.0366906
H	-1.2742049	-5.4154226	2.0593221
H	2.0873856	8.0100001	-2.2617732
H	0.5806358	8.3143295	-1.3104205
C	1.8913220	-2.7500441	-0.3088574
N	2.8833352	-3.3526607	-0.5207550
N	-2.1385649	-0.3652429	1.5025291
H	-2.4939594	0.6035496	1.6368547
H	-2.9371688	-1.0009815	1.6229574
H	-1.5155803	-0.5314922	2.3019451

4

Co	0.3790076	-1.9265099	-0.0345859
N	0.2856509	-1.1013555	-1.7070600
N	-0.4514597	-3.4927597	-0.7959392
N	0.2874604	-2.5276687	1.7876470
N	1.1165562	-0.3048375	0.5208334
C	0.4900683	0.3760243	-1.7202654
C	-0.8536428	1.0386397	-1.3726140
C	0.9758501	0.6563880	-3.1974202
C	0.5839631	2.0447827	-3.7178599
C	2.5030450	0.4668269	-3.3361626
C	0.3063593	-0.5322849	-3.9832353
C	-1.0335836	-0.2631353	-4.6936724
C	0.1802976	-1.6048923	-2.9127439
C	-0.0749055	-2.9997013	-3.1783334
C	0.0697244	-3.4882137	-4.6047889
C	-0.4445489	-3.8482385	-2.1466183
C	-0.7906970	-5.3478906	-2.2762924
C	-1.6321579	-5.7848488	-3.4868587
C	0.5478917	-6.1306198	-2.2296252
C	-1.5474277	-5.5787903	-0.9313896

C	-3.0686616	-5.3270976	-0.9714426
C	-0.8904641	-4.5409943	-0.0572804
C	-0.7413018	-4.6890801	1.3219210
C	-0.1345922	-3.7635034	2.1666654
C	0.1898256	-4.0349195	3.6316808
C	1.6051027	-4.6695818	3.6729958
C	-0.8099987	-4.9455013	4.3546109
C	0.2623698	-2.5762056	4.1654086
C	-1.0715070	-2.0299749	4.7124801
C	0.6652359	-1.8019413	2.9133833
C	1.2001462	-0.5218540	2.9294304
C	1.4857812	0.1288890	4.2673196
C	1.4110684	0.2090084	1.6980342
C	1.8838305	1.6713233	1.5839005
C	3.3539804	1.8358316	2.0141108
C	0.9269338	2.6235420	2.3701119
C	-0.5294166	2.1378943	2.2518505
C	-1.7319590	3.0552937	2.0420558
O	-2.8554619	2.5197455	1.9663638
N	-1.5159866	4.3889435	1.9227191
C	1.6904428	1.9632452	0.0442766
C	2.7312192	2.8695118	-0.6076534
C	1.5291952	0.5784118	-0.5912534
C	-2.5758953	5.2761102	1.4643484
C	-2.5094196	5.5307339	-0.0564426
C	-3.6726772	6.3896746	-0.5417914
O	-1.2977847	6.2198281	-0.3937929
O	-0.1825010	4.0869959	-1.4201437
O	0.8535289	5.2762093	0.7253764
P	0.1172609	5.3497820	-0.6274609
O	0.9100516	6.4034383	-1.6172635
C	1.3538827	7.6388463	-1.0614908
H	1.8428377	7.4838416	-0.0747926
H	-3.6623503	7.3760064	-0.0336070
H	-3.5804707	6.5649521	-1.6311650
H	-4.6452432	5.8966112	-0.3392930
H	-2.5224247	4.5434317	-0.5729113
H	-3.5464224	4.8092959	1.7251607
H	-2.4897458	6.2510438	1.9897559

H	-0.5426072	4.7329041	1.7418701
H	-0.6145469	1.4738582	1.3704680
H	-0.8101017	1.4879361	3.1068376
H	1.2303822	2.7192223	3.4317334
H	1.0631626	3.6252577	1.9069672
H	3.6633910	2.8894798	1.8773680
H	3.5205816	1.5833076	3.0783406
H	4.0284577	1.1992512	1.4070815
H	2.4073902	3.1237277	-1.6340402
H	3.7414460	2.4129179	-0.6539036
H	0.7304538	2.4996220	-0.0725989
H	2.4989561	0.1631516	-0.9360419
H	-1.6157471	0.7765523	-2.1299380
H	-1.2172773	0.6362125	-0.4083411
H	-0.7590137	2.1430246	-1.3294800
H	0.9996932	2.2055681	-4.7343668
H	-0.5094499	2.1913688	-3.7690807
H	0.9435636	2.8493014	-3.0484007
H	3.0658997	1.2533534	-2.7999735
H	2.8321027	-0.5236346	-2.9562677
H	1.0195034	-0.8790953	-4.7616689
H	-1.4058815	-1.1804196	-5.1910268
H	-1.8147139	0.0758909	-3.9876426
H	0.7104204	-2.8056585	-5.1915465
H	0.5462406	-4.4843543	-4.6488602
H	-0.9006055	-3.5598994	-5.1384564
H	-1.0323012	-5.8778383	-4.4090299
H	-2.0639348	-6.7872734	-3.2874605
H	-2.4670333	-5.0875907	-3.6906556
H	1.1705145	-5.9076224	-3.1178127
H	1.1373197	-5.8553189	-1.3318099
H	-1.3598429	-6.6034826	-0.5473318
H	-3.4897711	-5.3794486	0.0523367
H	-3.2931013	-4.3194141	-1.3766696
H	-0.7388622	-5.9869746	3.9819026
H	-0.5870697	-4.9747418	5.4404433
H	-1.8566985	-4.6101688	4.2241601
H	2.3478922	-4.0202428	3.1666257
H	1.9281701	-4.8201285	4.7237981

H	1.6091245	-5.6517881	3.1601683
H	1.0355577	-2.4960304	4.9555458
H	-1.8809671	-2.1337667	3.9612609
H	-1.3843667	-2.5640018	5.6308844
H	2.3720516	0.7835881	4.2316804
H	0.6381513	0.7627397	4.6034894
H	1.6690120	-0.6192074	5.0589952
H	2.7752542	3.8331490	-0.0643481
H	2.7870307	0.5313642	-4.4065259
H	0.3532432	-7.2227047	-2.2089768
H	-3.5935621	-6.0790271	-1.5920342
H	-0.9116459	0.5145651	-5.4718727
H	-0.9848803	-0.9550818	4.9611091
H	-1.0934281	-5.6316446	1.7612676
H	2.0826506	8.0843354	-1.7693523
H	0.5055335	8.3472562	-0.9258854
O	2.0409602	-2.6549355	-0.3885589
H	2.6236115	-2.2632109	0.2950364
N	-2.1885298	-0.5738931	1.3606129
H	-2.6002593	0.3741249	1.4715035
H	-2.9556498	-1.2532551	1.4372393
H	-1.5964078	-0.7084704	2.1891117

5

Co	0.0764237	-1.8063865	0.0168696
N	-0.0600412	-1.0484461	-1.6862961
N	-0.8489272	-3.3715973	-0.6399686
N	0.0096646	-2.2962882	1.8790166
N	0.9033481	-0.1863782	0.4660525
C	0.1667940	0.4239772	-1.7472538
C	-1.1320698	1.1170702	-1.2992795
C	0.5218532	0.6695444	-3.2647846
C	0.1327630	2.0701575	-3.7578785
C	2.0226940	0.4311113	-3.5443406
C	-0.2600200	-0.5062032	-3.9677389
C	-1.6403505	-0.1876617	-4.5724820
C	-0.3346661	-1.5562993	-2.8666261
C	-0.6999979	-2.9405340	-3.0661440
C	-0.7515938	-3.4592204	-4.4907604

C	-1.0094805	-3.7477987	-1.9766951
C	-1.4658166	-5.2245359	-2.0329770
C	-2.4573379	-5.6198621	-3.1410553
C	-0.1933187	-6.1064907	-2.1156572
C	-2.0899981	-5.3850387	-0.6113255
C	-3.5945960	-5.0611656	-0.5081860
C	-1.3056136	-4.3602082	0.1713763
C	-1.1160219	-4.4373683	1.5520176
C	-0.4756401	-3.4795533	2.3394195
C	-0.1664882	-3.6718361	3.8219215
C	1.1891302	-4.4193167	3.9173584
C	-1.2387818	-4.4434669	4.6020462
C	0.0299361	-2.1908540	4.2592083
C	-1.2351935	-1.5058109	4.8132442
C	0.4506798	-1.5241233	2.9520889
C	1.0774395	-0.2865063	2.8755798
C	1.4693605	0.4108024	4.1627731
C	1.2743102	0.3726124	1.5991577
C	1.8715466	1.7765466	1.3836287
C	3.3826359	1.7805702	1.6908805
C	1.1043872	2.8702334	2.1881751
C	-0.4016301	2.5708430	2.1943858
C	-1.4793087	3.6494911	2.2540276
O	-2.6565117	3.2895036	2.4360194
N	-1.1022440	4.9389366	2.0409374
C	1.5874937	2.0210594	-0.1488875
C	2.6367035	2.8338592	-0.9036234
C	1.2951778	0.6256074	-0.7060842
C	-2.0936947	5.9473239	1.6952257
C	-2.2934240	6.0618270	0.1675033
C	-3.4054183	7.0426711	-0.1903651
O	-1.0935809	6.5298708	-0.4662679
O	-0.4117625	4.1553599	-1.3396042
O	1.1138764	5.4647808	0.4056113
P	0.1534590	5.4597182	-0.7997453
O	0.8591745	6.2676162	-2.0551203
C	1.5032213	7.5063849	-1.7726111
H	2.1404977	7.4308324	-0.8644164
H	-3.1670082	8.0552321	0.1968922

H	-3.5055956	7.1126798	-1.2909396
H	-4.3755846	6.7181027	0.2375862
H	-2.5384275	5.0468075	-0.2228536
H	-3.0516135	5.6646030	2.1757024
H	-1.7669397	6.9343764	2.0859668
H	-0.1422747	5.1291189	1.6702409
H	-0.6643675	2.0507294	1.2504380
H	-0.6684405	1.8522666	2.9952784
H	1.4949706	2.9709372	3.2208892
H	1.3190892	3.8285896	1.6680501
H	3.8040342	2.7857770	1.4964640
H	3.5999787	1.5326210	2.7478365
H	3.9249848	1.0507467	1.0556854
H	2.2610931	3.0823345	-1.9144269
H	3.6079708	2.3064649	-1.0078469
H	0.6564192	2.6144034	-0.2246459
H	2.2189092	0.1463870	-1.0983632
H	-1.9556096	0.8677183	-1.9939950
H	-1.4425251	0.7366717	-0.3050223
H	-1.0115191	2.2185784	-1.2736366
H	0.4717479	2.2224907	-4.8038187
H	-0.9562925	2.2482136	-3.7231121
H	0.5677923	2.8625496	-3.1181864
H	2.6582583	1.1985816	-3.0651541
H	2.3557763	-0.5676575	-3.1892844
H	0.3697150	-0.8953103	-4.7980032
H	-2.0902819	-1.0932598	-5.0246487
H	-2.3470883	0.1922940	-3.8110710
H	-0.1426861	-2.8263676	-5.1623549
H	-0.3508843	-4.4869840	-4.5731445
H	-1.7783885	-3.4766092	-4.9117753
H	-1.9650531	-5.7657097	-4.1185200
H	-2.9339990	-6.5864455	-2.8777081
H	-3.2588818	-4.8682838	-3.2710792
H	0.3598738	-5.9239380	-3.0593831
H	0.4908877	-5.8934312	-1.2693154
H	-1.9155091	-6.4106423	-0.2212009
H	-3.9101030	-5.0725513	0.5538186
H	-3.8097184	-4.0502432	-0.9102150

H	-1.2626483	-5.5087599	4.2956193
H	-1.0156821	-4.4239466	5.6881624
H	-2.2516104	-4.0247444	4.4476235
H	1.9871507	-3.8656779	3.3812441
H	1.4954312	-4.5320135	4.9782374
H	1.1135427	-5.4285279	3.4651888
H	0.8322187	-2.1245783	5.0220695
H	-2.0668148	-1.5541161	4.0821194
H	-1.5716381	-1.9727648	5.7593603
H	2.4038387	0.9873684	4.0548929
H	0.6920253	1.1308043	4.4953483
H	1.6263850	-0.3062812	4.9885911
H	2.7874250	3.8082459	-0.3990629
H	2.2106278	0.4812721	-4.6367823
H	-0.4639221	-7.1823168	-2.0828881
H	-4.2164362	-5.7965084	-1.0551285
H	-1.5507057	0.5755914	-5.3694787
H	-1.0432914	-0.4346265	5.0140182
H	-1.5061694	-5.3329137	2.0544246
H	2.1351948	7.7660982	-2.6468507
H	0.7597526	8.3198590	-1.6123958
N	1.9613348	-2.6854937	-0.2952797
C	3.0396564	-2.5636526	0.5576487
C	2.3918713	-3.3046717	-1.3894794
C	4.1550950	-3.1293107	-0.0359613
N	3.7227517	-3.5933947	-1.2675743
H	1.7747589	-3.5421586	-2.2638271
H	5.1912832	-3.2372590	0.2983507
H	4.3006961	-4.0580102	-1.9693480
H	2.9363211	-2.0651767	1.5284097
N	-2.4315935	0.0204755	1.6362599
H	-2.7084087	0.9799997	1.9121578
H	-3.1539640	-0.6175091	1.9931971
H	-1.5767014	-0.1846821	2.1681369

6

Co	0.1550391	-1.9906369	0.0675128
N	0.0586453	-1.2653887	-1.6434366
N	-0.4989117	-3.6654657	-0.6222737

N	0.0706301	-2.4667377	1.9087893
N	0.8963303	-0.3333890	0.5230756
C	0.1927833	0.2190947	-1.6907435
C	-1.1355498	0.8342882	-1.2118931
C	0.5201126	0.5033537	-3.2052863
C	0.0350885	1.8808476	-3.6790843
C	2.0313575	0.3695672	-3.4974581
C	-0.1764979	-0.7182633	-3.9170694
C	-1.5961127	-0.5099071	-4.4778038
C	-0.1467636	-1.7885980	-2.8338413
C	-0.3371916	-3.2045410	-3.0453789
C	-0.3156578	-3.7102979	-4.4753474
C	-0.5615218	-4.0551720	-1.9638042
C	-0.7878048	-5.5831666	-2.0333634
C	-1.6685464	-6.1239485	-3.1728554
C	0.6111243	-6.2528683	-2.0748021
C	-1.4254327	-5.8453264	-0.6334185
C	-2.9653641	-5.7676593	-0.5795582
C	-0.8418956	-4.7152805	0.1775446
C	-0.7204148	-4.7506039	1.5635088
C	-0.2745340	-3.6971731	2.3674637
C	-0.0525176	-3.8154447	3.8707120
C	1.3889582	-4.3498510	4.0800365
C	-1.0563729	-4.7221644	4.5943606
C	-0.1113180	-2.3141429	4.2743212
C	-1.5095860	-1.8014607	4.6731077
C	0.3352953	-1.6216684	2.9929571
C	0.8446082	-0.3346841	2.9355562
C	1.0636154	0.4360078	4.2203028
C	1.1349738	0.2869462	1.6590819
C	1.7941222	1.6589771	1.4310106
C	3.2903385	1.5395826	1.8026921
C	1.1295105	2.8457240	2.1842034
C	-0.4024457	2.8240443	2.1251801
C	-1.1800373	4.0962961	2.4709577
O	-2.2984205	4.0258267	2.9938122
N	-0.6078538	5.2759563	2.0677086
C	1.5911270	1.8697559	-0.1185642
C	2.7086700	2.6276294	-0.8336117

C	1.3140805	0.4611768	-0.6503799
C	-1.4532814	6.4271256	1.7949179
C	-2.0497108	6.3708860	0.3664351
C	-3.0099411	7.5250617	0.1009920
O	-1.0150016	6.4467700	-0.6293368
O	-0.9145482	3.8571171	-1.0157659
O	1.1878047	5.1855168	-0.0722547
P	-0.0593205	5.1161360	-0.9703442
O	0.3695913	5.4820079	-2.5255631
C	1.2279787	6.6019086	-2.7357463
H	2.0746182	6.6004808	-2.0148147
H	-2.4847767	8.4967138	0.2090470
H	-3.4021817	7.4609708	-0.9326304
H	-3.8629110	7.5018691	0.8090985
H	-2.5794518	5.3960473	0.2609875
H	-2.2788584	6.4364798	2.5348593
H	-0.8577394	7.3582755	1.8997863
H	0.2404526	5.2268576	1.4596501
H	-0.7221840	2.6303671	1.0778615
H	-0.8358952	2.0162486	2.7476086
H	1.4807786	2.9055767	3.2334032
H	1.5100399	3.7533906	1.6727111
H	3.8031698	2.5037708	1.6208595
H	3.4293264	1.2851030	2.8717208
H	3.7961590	0.7565984	1.2028601
H	2.3854145	2.9028892	-1.8563308
H	3.6490557	2.0424326	-0.9060839
H	0.6719548	2.4723435	-0.2511550
H	2.2446873	-0.0161984	-1.0267464
H	-1.9746702	0.4871037	-1.8429614
H	-1.3429912	0.5050450	-0.1740845
H	-1.1036262	1.9449113	-1.2255767
H	0.3241439	2.0500140	-4.7368425
H	-1.0598585	2.0036101	-3.6014214
H	0.4662180	2.6972968	-3.0677594
H	2.6204028	1.1614119	-3.0019906
H	2.4348701	-0.6132401	-3.1733903
H	0.4622470	-1.0347582	-4.7696609
H	-1.9769065	-1.4441882	-4.9360460

H	-2.3120551	-0.2055476	-3.6915402
H	0.1945651	-2.9900383	-5.1400549
H	0.2296590	-4.6672196	-4.5680244
H	-1.3321290	-3.8682980	-4.8914611
H	-1.1262024	-6.1938123	-4.1316355
H	-1.9987264	-7.1519651	-2.9198776
H	-2.5721853	-5.5060401	-3.3341754
H	1.1613405	-5.9776648	-2.9972973
H	1.2273282	-5.9481265	-1.2038769
H	-1.1012292	-6.8304990	-0.2371990
H	-3.3104162	-5.8361051	0.4710119
H	-3.3284288	-4.8036767	-0.9903050
H	-0.8977087	-5.7863559	4.3253320
H	-0.9214305	-4.6436592	5.6922541
H	-2.1057684	-4.4640452	4.3546508
H	2.1338998	-3.6972580	3.5814868
H	1.6271630	-4.3932251	5.1626192
H	1.4946976	-5.3699535	3.6588846
H	0.5946225	-2.1201985	5.1059333
H	-2.2464608	-1.9968137	3.8677514
H	-1.8709259	-2.2839368	5.6023581
H	2.0189378	0.9906884	4.2067403
H	0.2619643	1.1839096	4.3904021
H	1.0881060	-0.2272333	5.1023070
H	2.8941645	3.5933137	-0.3284296
H	2.2078888	0.4585179	-4.5889360
H	0.5098634	-7.3573216	-2.0552596
H	-3.4413945	-6.5910871	-1.1465426
H	-1.5942505	0.2724401	-5.2613779
H	-1.4865458	-0.7080473	4.8454508
H	-1.0035842	-5.6873106	2.0623535
H	1.6222578	6.5352115	-3.7703068
H	0.6711001	7.5592548	-2.6237246
N	3.0083904	-2.9792105	0.1063508
C	2.4490360	-3.0617285	-0.9195713
H	1.9893815	-3.1878197	-1.9031471

7

Co	0.5353185	-1.7147686	-0.0109337

N	0.6227899	-0.9176523	-1.6834035
N	-0.2176031	-3.2831274	-0.8169679
N	0.2137295	-2.2492271	1.7890883
N	1.2219462	-0.0872916	0.5782859
C	0.7837021	0.5701166	-1.7030005
C	-0.5744142	1.2335490	-1.4371448
C	1.3509221	0.8474271	-3.1531261
C	0.9604311	2.2082606	-3.7410607
C	2.8889283	0.7140392	-3.1872080
C	0.7610836	-0.3648295	-3.9633788
C	-0.5520883	-0.1302190	-4.7359115
C	0.5962352	-1.4309715	-2.8949267
C	0.3619826	-2.8264206	-3.1643372
C	0.6160102	-3.3292047	-4.5713477
C	-0.1190934	-3.6544639	-2.1588708
C	-0.5498051	-5.1281634	-2.3129588
C	-1.3118809	-5.5073870	-3.5935964
C	0.7168744	-6.0089538	-2.1577374
C	-1.4353574	-5.2982732	-1.0370803
C	-2.9335064	-4.9756066	-1.2125764
C	-0.8153878	-4.2800148	-0.1155052
C	-0.8698676	-4.3719058	1.2744330
C	-0.3443503	-3.4369957	2.1604810
C	-0.2332060	-3.6637597	3.6660893
C	1.0755611	-4.4564540	3.9181681
C	-1.4227145	-4.4089177	4.2850103
C	-0.0469542	-2.1984285	4.1484909
C	-1.3671333	-1.4626470	4.4657987
C	0.5766131	-1.5416713	2.9274923
C	1.2833021	-0.3505815	2.9761238
C	1.7622353	0.1322281	4.3322232
C	1.5129164	0.4000396	1.7740449
C	2.1299362	1.8066177	1.6715676
C	3.6589363	1.6756675	1.8810263
C	1.5516355	2.9029548	2.6151220
C	0.1337385	2.7214433	3.2010848
C	-1.0310303	2.3392633	2.2801267
O	-1.5249594	1.1848875	2.3508351
N	-1.5058259	3.3298754	1.5082261

C	1.7908108	2.1804149	0.1838756
C	2.7163051	3.2014109	-0.4683829
C	1.7102732	0.8121730	-0.4945144
C	-2.7485843	3.2860473	0.7551778
C	-2.6431904	4.0094185	-0.6050736
C	-4.0190862	4.2342048	-1.2243078
O	-2.0080381	5.2802907	-0.4438855
O	0.0916635	4.4494980	-1.7755065
O	0.2756147	5.2161439	0.7723353
P	-0.3277255	5.3656392	-0.6410822
O	-0.2054136	6.9296054	-1.1332670
C	-0.4792914	7.9582217	-0.1836243
H	0.0228499	7.7534642	0.7868200
H	-4.6293037	4.8987805	-0.5786077
H	-3.9087288	4.7209299	-2.2124186
H	-4.5603007	3.2747567	-1.3571106
H	-2.0313181	3.3922188	-1.2953386
H	-3.0556178	2.2305493	0.6073348
H	-3.5471626	3.7816561	1.3554940
H	-0.8974425	4.1962905	1.4055885
H	0.1270603	1.9491417	3.9893076
H	-0.1310899	3.6808941	3.6924043
H	2.2334847	3.0532216	3.4794888
H	1.5634642	3.8552398	2.0441576
H	4.1442390	2.6670063	1.7876006
H	3.8978871	1.2759458	2.8861299
H	4.1192888	0.9978737	1.1329972
H	2.2837075	3.5351903	-1.4282625
H	3.7399624	2.8066813	-0.6317676
H	0.7767894	2.6248337	0.1948729
H	2.7284437	0.4491979	-0.7529629
H	-1.3494836	0.8722057	-2.1374070
H	-0.9102363	0.9965916	-0.4098169
H	-0.4661684	2.3317748	-1.5330531
H	1.3922120	2.3169036	-4.7578508
H	-0.1318374	2.3502975	-3.8203722
H	1.3130440	3.0470826	-3.1145592
H	3.3790207	1.5266266	-2.6184884
H	3.2353750	-0.2591663	-2.7797766

H	1.5199793	-0.6946502	-4.7048658
H	-0.8868155	-1.0621127	-5.2321637
H	-1.3687335	0.2017663	-4.0675238
H	1.3452360	-2.6823663	-5.0929809
H	1.0412808	-4.3493270	-4.5710433
H	-0.3000074	-3.3541612	-5.1970004
H	-0.6432258	-5.6389226	-4.4625454
H	-1.8251002	-6.4786217	-3.4385706
H	-2.0787126	-4.7548411	-3.8579760
H	1.4410360	-5.8181758	-2.9740261
H	1.2298726	-5.8039392	-1.1958513
H	-1.3295621	-6.3250856	-0.6272169
H	-3.4433042	-5.0185975	-0.2303409
H	-3.0750609	-3.9468032	-1.5968007
H	-1.4376777	-5.4701830	3.9642006
H	-1.3440104	-4.4052941	5.3910568
H	-2.3919761	-3.9563030	4.0030338
H	1.9532115	-3.9181515	3.5046541
H	1.2360606	-4.5965504	5.0074102
H	1.0296253	-5.4552812	3.4400997
H	0.6214648	-2.1611961	5.0310437
H	-2.0834716	-1.5768702	3.6277324
H	-1.8356721	-1.8556540	5.3891109
H	2.2917804	1.0965267	4.2761614
H	0.9312270	0.2597577	5.0558283
H	2.4675306	-0.6007097	4.7773842
H	2.7721961	4.1130850	0.1576495
H	3.2450739	0.7839146	-4.2353216
H	0.4432765	-7.0840644	-2.1837900
H	-3.4308415	-5.6975013	-1.8892806
H	-0.4142963	0.6373648	-5.5212151
H	-1.2019074	-0.3740879	4.5769193
H	-1.3397341	-5.2704636	1.6959145
H	-0.0991801	8.9115317	-0.6033429
H	-1.5733905	8.0558193	-0.0016926
C	2.3053551	-2.5329237	-0.0844542
H	2.2287499	-3.5291090	0.3959070
H	2.5889384	-2.6336050	-1.1511870
H	3.0274632	-1.8954103	0.4617236

N	-2.8246280	-1.7311963	-0.0014370
C	-2.5034684	-0.8609758	0.7114416
H	-2.1836060	0.0001223	1.3687728

8

Co	0.4752482	-1.6839524	-0.0093305
N	0.5403438	-0.8751544	-1.6861523
N	-0.3212461	-3.2341459	-0.8162564
N	0.1187121	-2.1988339	1.7955782
N	1.1666089	-0.0514894	0.5820901
C	0.7407890	0.6093229	-1.7031161
C	-0.6038982	1.3004932	-1.4420228
C	1.3205661	0.8664796	-3.1511591
C	0.9685207	2.2354755	-3.7431910
C	2.8543372	0.6857754	-3.1756442
C	0.7000559	-0.3307043	-3.9611874
C	-0.6054631	-0.0681093	-4.7375600
C	0.5088751	-1.3927465	-2.8943003
C	0.2618426	-2.7855881	-3.1631047
C	0.5182824	-3.2942735	-4.5663281
C	-0.2178464	-3.6110073	-2.1566763
C	-0.6389720	-5.0867466	-2.2976157
C	-1.3822561	-5.4919911	-3.5805981
C	0.6381853	-5.9479747	-2.1108648
C	-1.5411585	-5.2431142	-1.0316347
C	-3.0358736	-4.9143108	-1.2269995
C	-0.9293426	-4.2200630	-0.1115673
C	-0.9956938	-4.3041230	1.2782050
C	-0.4508364	-3.3814630	2.1636908
C	-0.3243768	-3.6176970	3.6650642
C	0.9846184	-4.4218722	3.8851017
C	-1.5104879	-4.3575771	4.2955675
C	-0.1193745	-2.1572354	4.1542944
C	-1.4291564	-1.4086049	4.4855703
C	0.5013099	-1.5008472	2.9328266
C	1.2236141	-0.3202320	2.9800909
C	1.7194396	0.1519822	4.3325381
C	1.4646355	0.4258608	1.7776829
C	2.1048237	1.8205419	1.6730079

C	3.6307259	1.6540356	1.8836852
C	1.5431804	2.9256690	2.6153516
C	0.1207510	2.7677243	3.1983140
C	-1.0457850	2.3985731	2.2739152
O	-1.5354909	1.2403550	2.3249178
N	-1.5245092	3.4018703	1.5222252
C	1.7736708	2.1999356	0.1845325
C	2.7201755	3.2021683	-0.4664446
C	1.6725726	0.8333148	-0.4952632
C	-2.7652247	3.3718825	0.7654747
C	-2.6465288	4.0842799	-0.5999929
C	-4.0183349	4.3283697	-1.2209008
O	-1.9896165	5.3447686	-0.4480727
O	0.1022763	4.4648612	-1.7589472
O	0.2857689	5.2512176	0.7825629
P	-0.3069075	5.3998644	-0.6356928
O	-0.1533812	6.9553628	-1.1417383
C	-0.4121998	7.9991801	-0.2038317
H	0.0818938	7.7951876	0.7707993
H	-4.6166006	5.0084195	-0.5802341
H	-3.8998061	4.8055856	-2.2126790
H	-4.5764239	3.3776436	-1.3463989
H	-2.0453133	3.4511306	-1.2851876
H	-3.0884025	2.3203149	0.6244104
H	-3.5564251	3.8846220	1.3607903
H	-0.9067219	4.2627121	1.4214637
H	0.1002862	1.9978232	3.9891881
H	-0.1302032	3.7323436	3.6860468
H	2.2265463	3.0663142	3.4798727
H	1.5690792	3.8767594	2.0428584
H	4.1361842	2.6340483	1.7812695
H	3.8603632	1.2597412	2.8927733
H	4.0744362	0.9563337	1.1447427
H	2.2942996	3.5448140	-1.4259914
H	3.7351642	2.7864693	-0.6291318
H	0.7682459	2.6635781	0.1935979
H	2.6818505	0.4454536	-0.7467954
H	-1.3834645	0.9560228	-2.1456746
H	-0.9489160	1.0724606	-0.4153244

H	-0.4705500	2.3966806	-1.5389091
H	1.4045224	2.3280555	-4.7595825
H	-0.1190879	2.4099648	-3.8225933
H	1.3472190	3.0642876	-3.1189180
H	3.3643504	1.4906297	-2.6134248
H	3.1711529	-0.2920918	-2.7559569
H	1.4529927	-0.6821089	-4.6984710
H	-0.9587466	-0.9927815	-5.2346401
H	-1.4167821	0.2839414	-4.0729601
H	1.2448733	-2.6475334	-5.0908586
H	0.9497763	-4.3113475	-4.5565999
H	-0.3986029	-3.3272775	-5.1899673
H	-0.7015715	-5.6323821	-4.4385096
H	-1.8876095	-6.4655389	-3.4148345
H	-2.1536305	-4.7520785	-3.8670812
H	1.3585617	-5.7760991	-2.9342351
H	1.1563700	-5.7003601	-1.1622298
H	-1.4429481	-6.2654271	-0.6099380
H	-3.5586322	-4.9466456	-0.2512302
H	-3.1683004	-3.8887438	-1.6232417
H	-1.5378659	-5.4166437	3.9689174
H	-1.4135022	-4.3618116	5.3999846
H	-2.4809347	-3.8954928	4.0330375
H	1.8564852	-3.8980679	3.4429292
H	1.1688171	-4.5580708	4.9708332
H	0.9153492	-5.4221121	3.4133757
H	0.5580001	-2.1316951	5.0301159
H	-2.1534419	-1.5083483	3.6523430
H	-1.8943180	-1.8031497	5.4096590
H	2.2470994	1.1169468	4.2773638
H	0.8973431	0.2710330	5.0671845
H	2.4317714	-0.5853011	4.7586122
H	2.7940331	4.1115503	0.1610038
H	3.2167709	0.7366034	-4.2225769
H	0.3740858	-7.0255449	-2.1013834
H	-3.5264492	-5.6406556	-1.9034376
H	-0.4461299	0.6954697	-5.5223640
H	-1.2501283	-0.3230847	4.6047088
H	-1.4737727	-5.1978368	1.7001137

H	-0.0122923	8.9403491	-0.6320823
H	-1.5050668	8.1189228	-0.0284880
C	2.1259076	-2.4287828	-0.0971289
N	3.1917059	-2.9292493	-0.1755602
N	-2.9046471	-1.6621018	0.0058452
C	-2.5689436	-0.7828687	0.7010597
H	-2.2295767	0.0810843	1.3474970

9

Co	0.4963445	-1.7199838	-0.0161698
N	0.5126709	-0.9057537	-1.6917407
N	-0.2334599	-3.3102510	-0.8117601
N	0.2093052	-2.2713508	1.7972968
N	1.1337569	-0.0721585	0.5788913
C	0.7189329	0.5745428	-1.7153559
C	-0.6164157	1.2860957	-1.4672300
C	1.3177371	0.8227279	-3.1585823
C	0.9786340	2.1885124	-3.7649000
C	2.8506899	0.6311134	-3.1620974
C	0.7068257	-0.3786119	-3.9669108
C	-0.6013994	-0.1261274	-4.7414591
C	0.5226725	-1.4357479	-2.8923787
C	0.3164150	-2.8370098	-3.1580810
C	0.5775253	-3.3376771	-4.5634781
C	-0.1364967	-3.6772982	-2.1507992
C	-0.5429753	-5.1581663	-2.3026223
C	-1.3079168	-5.5507425	-3.5774162
C	0.7393962	-6.0167915	-2.1515154
C	-1.4192188	-5.3385200	-1.0214462
C	-2.9203183	-5.0210715	-1.1856756
C	-0.8055507	-4.3124586	-0.1045735
C	-0.8513103	-4.4053259	1.2873842
C	-0.3245387	-3.4691081	2.1694502
C	-0.1940361	-3.6949482	3.6737857
C	1.1356370	-4.4579378	3.9083866
C	-1.3594698	-4.4665474	4.3052488
C	-0.0364833	-2.2257277	4.1551157
C	-1.3706365	-1.5141310	4.4719568
C	0.5660517	-1.5577044	2.9301410

C	1.2465730	-0.3494641	2.9742017
C	1.7260482	0.1395706	4.3272599
C	1.4580070	0.4034610	1.7700363
C	2.1102083	1.7928108	1.6528417
C	3.6363465	1.6127281	1.8489327
C	1.5751951	2.9111142	2.5958079
C	0.1617460	2.7724640	3.2041918
C	-1.0195717	2.3965515	2.3028776
O	-1.5301354	1.2500937	2.3959569
N	-1.4853277	3.3830947	1.5214209
C	1.7702950	2.1667813	0.1651224
C	2.7172527	3.1609684	-0.4969565
C	1.6480591	0.7964637	-0.5038014
C	-2.7350886	3.3520031	0.7793739
C	-2.6289365	4.0616920	-0.5883237
C	-4.0061097	4.3023202	-1.1986894
O	-1.9728180	5.3239160	-0.4437916
O	0.1019441	4.4623079	-1.7988696
O	0.3214466	5.2240417	0.7476953
P	-0.2934098	5.3836976	-0.6601963
O	-0.1533292	6.9458134	-1.1521064
C	-0.4006998	7.9778816	-0.1988379
H	0.1105951	7.7656483	0.7651723
H	-4.6004717	4.9827147	-0.5546906
H	-3.8959093	4.7777328	-2.1922921
H	-4.5634852	3.3503871	-1.3180180
H	-2.0326200	3.4288139	-1.2775527
H	-3.0609824	2.3004035	0.6443178
H	-3.5198647	3.8665072	1.3818301
H	-0.8615336	4.2371163	1.4000824
H	0.1477701	2.0155780	4.0071954
H	-0.0756607	3.7468206	3.6795843
H	2.2745642	3.0513392	3.4477166
H	1.6017552	3.8573321	2.0153632
H	4.1519598	2.5871526	1.7429475
H	3.8723797	1.2136515	2.8550521
H	4.0674095	0.9160925	1.1006152
H	2.2847559	3.5050365	-1.4531283
H	3.7280970	2.7386038	-0.6692976

H	0.7680648	2.6370203	0.1814804
H	2.6473236	0.3816695	-0.7535734
H	-1.3987376	0.9433612	-2.1686460
H	-0.9675174	1.0745756	-0.4394817
H	-0.4704520	2.3792877	-1.5757171
H	1.4281586	2.2726992	-4.7762375
H	-0.1076137	2.3642983	-3.8608693
H	1.3508451	3.0216047	-3.1421145
H	3.3611660	1.4364145	-2.6012749
H	3.1463563	-0.3469277	-2.7267505
H	1.4613863	-0.7243607	-4.7048804
H	-0.9507732	-1.0551986	-5.2333568
H	-1.4131146	0.2232206	-4.0758421
H	1.3070033	-2.6887799	-5.0819314
H	1.0061581	-4.3558855	-4.5610816
H	-0.3367381	-3.3641147	-5.1921415
H	-0.6428180	-5.6744333	-4.4502082
H	-1.8056688	-6.5292290	-3.4170009
H	-2.0872554	-4.8101850	-3.8395430
H	1.4497017	-5.8231425	-2.9788908
H	1.2624608	-5.7883966	-1.2008428
H	-1.3038396	-6.3644172	-0.6124454
H	-3.4253907	-5.0755840	-0.2013456
H	-3.0670142	-3.9897613	-1.5624537
H	-1.3545144	-5.5276187	3.9838485
H	-1.2674498	-4.4617273	5.4102664
H	-2.3419870	-4.0352440	4.0359089
H	1.9940434	-3.9062235	3.4732060
H	1.3173950	-4.5866446	4.9955683
H	1.1022603	-5.4603326	3.4372574
H	0.6337032	-2.1722214	5.0352256
H	-2.0880162	-1.6521312	3.6380889
H	-1.8273769	-1.9086449	5.4004253
H	2.2595611	1.1012319	4.2656095
H	0.8925372	0.2750956	5.0467016
H	2.4265477	-0.5932036	4.7797463
H	2.8033324	4.0713193	0.1275956
H	3.2270071	0.6669209	-4.2048519
H	0.4813371	-7.0959844	-2.1630682

H	-3.4185211	-5.7387742	-1.8660245
H	-0.4495778	0.6351770	-5.5302785
H	-1.2271945	-0.4212516	4.5729112
H	-1.3037309	-5.3118746	1.7107554
H	-0.0122446	8.9257097	-0.6231813
H	-1.4909220	8.0909454	-0.0033553
O	2.2239951	-2.3515130	-0.1930462
H	2.7078232	-1.9406903	0.5541211
N	-2.7033352	-1.6826341	0.0091829
C	-2.4147325	-0.8070224	0.7302174
H	-2.1294719	0.0593117	1.3998863

10

Co	0.1444959	-1.7088923	-0.0129767
N	0.1435722	-0.7530834	-1.6216842
N	-0.4882026	-3.2679200	-0.9541101
N	-0.0897141	-2.4334546	1.7503348
N	0.6851258	-0.0738902	0.7289961
C	0.2834838	0.7273666	-1.4993169
C	-1.0957525	1.3137097	-1.1668226
C	0.8282375	1.1567630	-2.9148017
C	0.4350665	2.5799426	-3.3323916
C	2.3649692	1.0201646	-2.9844074
C	0.2216975	0.0331277	-3.8378903
C	-1.0928771	0.3502524	-4.5761476
C	0.0780175	-1.1399811	-2.8781014
C	-0.1409603	-2.5101677	-3.2770297
C	-0.0026858	-2.8482297	-4.7499609
C	-0.4897830	-3.4733651	-2.3331589
C	-0.8226439	-4.9553913	-2.6242489
C	-1.6835640	-5.2553053	-3.8635960
C	0.5160318	-5.7342571	-2.6987391
C	-1.5524214	-5.3474457	-1.3017139
C	-3.0796279	-5.1259726	-1.2921654
C	-0.9224239	-4.3900531	-0.3228485
C	-0.8567232	-4.6363239	1.0480288
C	-0.4489492	-3.7176881	2.0129021
C	-0.3190376	-4.0362839	3.5007181
C	1.1192169	-4.5603715	3.7450703

C	-1.3383733	-5.0551424	4.0257760
C	-0.4542586	-2.6027076	4.0911994
C	-1.9073083	-2.1908820	4.4097858
C	0.0650755	-1.7446690	2.9448862
C	0.5395092	-0.4494689	3.1075671
C	0.7547772	0.0461040	4.5271626
C	0.8344606	0.3759117	1.9601533
C	1.4556615	1.7832720	1.9780609
C	2.9264289	1.6271338	2.4379571
C	0.7305719	2.8442216	2.8575882
C	-0.7824096	2.6550882	3.0870401
C	-1.7710582	2.7905253	1.9233404
O	-2.6316892	1.8998912	1.7192908
N	-1.7321487	3.9416640	1.2260699
C	1.3697713	2.2064447	0.4617449
C	2.5149516	3.0936183	-0.0209357
C	1.2188801	0.8705743	-0.2746496
C	-2.7079234	4.2752139	0.1979772
C	-2.0569982	4.9306291	-1.0314502
C	-3.0886635	5.3078471	-2.0906396
O	-1.3520728	6.1123912	-0.6423615
O	0.9286576	5.2573265	-1.6189596
O	0.5137096	5.4197043	1.0122760
P	0.3182850	5.9582895	-0.4243986
O	0.7107395	7.5561322	-0.4671479
C	0.2825463	8.3812382	0.6131972
H	0.5104187	7.9107350	1.5944588
H	-3.8067565	6.0495178	-1.6836617
H	-2.5743458	5.7661178	-2.9569176
H	-3.6549608	4.4187210	-2.4365429
H	-1.3273446	4.2183698	-1.4819389
H	-3.2497521	3.3496618	-0.0845212
H	-3.4581551	4.9876405	0.6127887
H	-0.9017093	4.6002103	1.3280295
H	-1.0023872	1.6663904	3.5299956
H	-1.1000054	3.4124978	3.8363530
H	1.2082378	2.8862515	3.8593612
H	0.9204790	3.8377174	2.3959733
H	3.4342585	2.6107780	2.4533829

H	2.9856968	1.1988359	3.4577594
H	3.4921505	0.9588016	1.7569492
H	2.2798748	3.5376941	-1.0055943
H	3.4783662	2.5452982	-0.0719840
H	0.4361627	2.7868903	0.3436515
H	2.2173184	0.4716158	-0.5571148
H	-1.8465650	1.0476142	-1.9319781
H	-1.4481634	0.9275130	-0.1960049
H	-1.0452233	2.4120469	-1.0897459
H	0.8541061	2.8113758	-4.3338307
H	-0.6603255	2.7159973	-3.3992454
H	0.8072330	3.3554174	-2.6308546
H	2.8682531	1.7647657	-2.3408907
H	2.7053349	0.0065938	-2.6816959
H	0.9738155	-0.2191134	-4.6164487
H	-1.4265478	-0.5194996	-5.1748031
H	-1.9104778	0.6033994	-3.8755598
H	0.6269566	-2.1023081	-5.2692612
H	0.4798297	-3.8310762	-4.9060931
H	-0.9749490	-2.8744581	-5.2844751
H	-1.1008593	-5.2373790	-4.8014717
H	-2.1103833	-6.2753808	-3.7736670
H	-2.5236678	-4.5426313	-3.9671603
H	1.1355054	-5.3894747	-3.5513697
H	1.1038106	-5.5983582	-1.7681163
H	-1.3363184	-6.4040342	-1.0354624
H	-3.4786719	-5.3131865	-0.2761370
H	-3.3271556	-4.0758122	-1.5443200
H	-1.1228111	-6.0696316	3.6329537
H	-1.2869807	-5.1176381	5.1319024
H	-2.3749904	-4.7953702	3.7385873
H	1.8751298	-3.8249665	3.4015828
H	1.2834654	-4.7496450	4.8263060
H	1.2909495	-5.5072110	3.1944144
H	0.1677468	-2.4967437	5.0022271
H	-2.5572332	-2.3510762	3.5269457
H	-2.3154278	-2.7689429	5.2620729
H	1.0983407	1.0907162	4.5633775
H	-0.1707853	-0.0070157	5.1353587

H	1.5179206	-0.5661252	5.0524861
H	2.6202005	3.9657807	0.6520278
H	2.7074053	1.1960819	-4.0247063
H	0.3255247	-6.8191932	-2.8336037
H	-3.6007736	-5.8026085	-1.9973633
H	-0.9574042	1.2022487	-5.2699322
H	-1.9645708	-1.1155200	4.6690057
H	-1.1918531	-5.6243736	1.3900968
H	0.8221556	9.3467698	0.5321213
H	-0.8127403	8.5761107	0.5625197
N	2.1526634	-2.3072632	-0.1566457
C	3.1053866	-2.1362781	0.8269690
C	2.7787257	-2.7531999	-1.2405587
C	4.3457311	-2.4913241	0.3244568
N	4.1162212	-2.8800153	-0.9854244
H	2.2965246	-2.9781918	-2.1992711
H	5.3451321	-2.4992035	0.7699094
H	4.8246427	-3.1974204	-1.6486311
H	2.8290377	-1.7579328	1.8178672
N	-3.0615726	-1.9341111	0.7356228
C	-2.9444819	-0.8494835	1.1585322
H	-2.8124081	0.2034287	1.5298418

12

Co	0.6457395	-1.7086008	0.0498823
N	0.7496347	-0.9073791	-1.6195776
N	-0.0740516	-3.2907841	-0.7763706
N	0.2747065	-2.2424317	1.8437091
N	1.3000957	-0.0718581	0.6506292
C	0.8773961	0.5840938	-1.6313361
C	-0.4945993	1.2209885	-1.3689333
C	1.4423953	0.8791066	-3.0782100
C	1.0210264	2.2325497	-3.6618058
C	2.9830059	0.7816103	-3.1055963
C	0.8832065	-0.3440282	-3.8945815
C	-0.4410795	-0.1476398	-4.6594556
C	0.7448454	-1.4182537	-2.8314719
C	0.5511269	-2.8191452	-3.1076067
C	0.8391317	-3.3079347	-4.5128419

C	0.0660544	-3.6591742	-2.1158166
C	-0.3204895	-5.1433818	-2.2799984
C	-1.0540549	-5.5352870	-3.5733721
C	0.9713722	-5.9850676	-2.1134158
C	-1.2170195	-5.3532022	-1.0164529
C	-2.7298928	-5.1278606	-1.2154485
C	-0.6696431	-4.2978626	-0.0892969
C	-0.7826465	-4.3719187	1.2999894
C	-0.3013781	-3.4253261	2.2003623
C	-0.2643703	-3.6340218	3.7122630
C	1.0300031	-4.4223987	4.0396595
C	-1.4834428	-4.3722899	4.2797094
C	-0.1034365	-2.1625791	4.1852267
C	-1.4377962	-1.4226908	4.4257036
C	0.5779341	-1.5181807	2.9881935
C	1.2671527	-0.3178181	3.0528982
C	1.6812106	0.1856529	4.4234936
C	1.5323199	0.4273402	1.8531037
C	2.1010613	1.8536663	1.7718470
C	3.6173299	1.7923481	2.0775231
C	1.4078532	2.9173996	2.6748420
C	-0.0393578	2.6561866	3.1496640
C	-1.1393932	2.3078574	2.1372557
O	-1.6470108	1.1577568	2.1412606
N	-1.5483422	3.3270605	1.3612028
C	1.8378962	2.2100404	0.2636997
C	2.7967821	3.2245356	-0.3502552
C	1.7915867	0.8376637	-0.4126348
C	-2.7326322	3.3204516	0.5179506
C	-2.5365350	4.1122581	-0.7934869
C	-3.8698996	4.3759216	-1.4864976
O	-1.9078334	5.3674811	-0.5326018
O	0.2773792	4.5020110	-1.6848426
O	0.2665235	5.2858711	0.8650427
P	-0.2148599	5.4336722	-0.5935417
O	-0.0289772	6.9919440	-1.0842828
C	-0.3738078	8.0315911	-0.1704758
H	0.0382568	7.8298320	0.8421421
H	-4.5181294	5.0122371	-0.8493868

H	-3.6960855	4.9085455	-2.4412382
H	-4.4050356	3.4277258	-1.7016763
H	-1.8842583	3.5241752	-1.4731830
H	-3.0141492	2.2749962	0.2816041
H	-3.5797079	3.7757312	1.0835635
H	-0.9428790	4.1998916	1.3492647
H	-0.0728815	1.8346854	3.8855497
H	-0.3674992	3.5741718	3.6812962
H	2.0136195	3.0872037	3.5908904
H	1.4195180	3.8774108	2.1156431
H	4.0627953	2.8043039	2.0110631
H	3.8122818	1.4040685	3.0962817
H	4.1516137	1.1355165	1.3607757
H	2.4201771	3.5439119	-1.3379568
H	3.8287601	2.8298892	-0.4486098
H	0.8258658	2.6555906	0.2156162
H	2.8176479	0.4891441	-0.6596411
H	-1.2570629	0.8551015	-2.0804681
H	-0.8366861	0.9745823	-0.3466269
H	-0.4032239	2.3201446	-1.4543780
H	1.4683566	2.3639356	-4.6691998
H	-0.0740585	2.3343191	-3.7657381
H	1.3294747	3.0788862	-3.0212607
H	3.4508542	1.6029956	-2.5307409
H	3.3494563	-0.1849638	-2.6999881
H	1.6485929	-0.6484310	-4.6397843
H	-0.7357160	-1.0826759	-5.1747594
H	-1.2704885	0.1275182	-3.9813742
H	1.5623780	-2.6423405	-5.0191507
H	1.2863225	-4.3185998	-4.5122190
H	-0.0670709	-3.3479939	-5.1520569
H	-0.3713288	-5.6330965	-4.4356828
H	-1.5335900	-6.5259478	-3.4352848
H	-1.8438898	-4.8064982	-3.8362037
H	1.7000301	-5.7676891	-2.9189669
H	1.4656631	-5.7688931	-1.1439971
H	-1.0530360	-6.3683324	-0.5956944
H	-3.2489266	-5.2132938	-0.2398114
H	-2.9279634	-4.1090596	-1.6018492

H	-1.4833816	-5.4369302	3.9700303
H	-1.4595989	-4.3568303	5.3882737
H	-2.4373426	-3.9223419	3.9455837
H	1.9270104	-3.8885018	3.6639687
H	1.1354706	-4.5489229	5.1371551
H	1.0083017	-5.4269793	3.5722757
H	0.5197743	-2.1153064	5.0996128
H	-2.0961028	-1.5096817	3.5385542
H	-1.9693315	-1.8256128	5.3100668
H	2.1666523	1.1734122	4.3845746
H	0.8240465	0.2751873	5.1216997
H	2.4052120	-0.5145668	4.8910925
H	2.8157570	4.1449480	0.2654807
H	3.3431526	0.8637834	-4.1514759
H	0.7315985	-7.0679959	-2.1477120
H	-3.1684762	-5.8830459	-1.8964503
H	-0.3387260	0.6417545	-5.4285225
H	-1.2709502	-0.3387192	4.5731051
H	-1.2564259	-5.2733835	1.7111515
H	0.0523255	8.9772219	-0.5625734
H	-1.4785687	8.1425069	-0.0866177
C	2.4300105	-2.5111337	0.0235657
H	2.3460985	-3.5196038	0.4760557
H	2.7544511	-2.5820526	-1.0338185
H	3.1212408	-1.8778251	0.6128125
O	-2.9105208	-1.8675099	-1.2214007
C	-2.5346028	-1.2213474	-0.2464914
H	-1.4775013	-1.3138442	0.1572162
N	-3.2514502	-0.3537492	0.4987919
H	-4.2067930	-0.1403670	0.2020499
H	-2.7523027	0.2685210	1.1830052

13

Co	0.4196225	-2.0237904	-0.0838686
N	0.1249606	-1.2712742	-1.7656239
N	-0.4449607	-3.6328656	-0.7006662
N	0.4204298	-2.4801122	1.7665839
N	1.1607401	-0.3512424	0.3164491
C	0.2805350	0.2125773	-1.8318368

C	-1.0077203	0.8320917	-1.2607001
C	0.4977410	0.4874251	-3.3649487
C	-0.0072538	1.8714437	-3.7991378
C	1.9813565	0.3302334	-3.7660203
C	-0.2699383	-0.7250122	-4.0153612
C	-1.7189968	-0.4967459	-4.4850426
C	-0.1830376	-1.7921167	-2.9325225
C	-0.4318358	-3.1969609	-3.1286955
C	-0.4960438	-3.7126654	-4.5516697
C	-0.5918068	-4.0349014	-2.0339438
C	-0.8308497	-5.5590426	-2.0721501
C	-1.7695459	-6.1167949	-3.1545404
C	0.5700924	-6.2209255	-2.1718002
C	-1.3993548	-5.7952737	-0.6396447
C	-2.9315545	-5.6788296	-0.5033005
C	-0.7432195	-4.6731723	0.1230444
C	-0.5043260	-4.7200636	1.4927309
C	0.0581143	-3.6947106	2.2524689
C	0.3960374	-3.8289384	3.7310598
C	1.8235128	-4.4319796	3.8171272
C	-0.5867077	-4.6901422	4.5339516
C	0.4407634	-2.3280538	4.1356341
C	-0.8966512	-1.7576838	4.6479776
C	0.8085276	-1.6541807	2.8209846
C	1.3431846	-0.3817334	2.7229335
C	1.6730924	0.3745281	3.9906327
C	1.5043729	0.2495973	1.4343999
C	2.0875328	1.6493665	1.1675078
C	3.6093359	1.6152761	1.4306006
C	1.3958426	2.7838763	1.9735031
C	-0.1284746	2.6264119	1.9866608
C	-1.0229040	3.8110517	2.3039927
O	-2.2048847	3.6088710	2.6685164
N	-0.5379508	5.0571589	2.0788313
C	1.7622061	1.8628086	-0.3609879
C	2.7920800	2.6642171	-1.1557105
C	1.4759095	0.4522622	-0.8827843
C	-1.4575497	6.1713048	1.8746203
C	-2.0144699	6.1957142	0.4309294

C	-3.0377832	7.3093443	0.2365044
O	-0.9639739	6.4127381	-0.5223663
O	-0.7174851	3.8598681	-1.0382628
O	1.3041183	5.2544819	-0.0114644
P	0.0709576	5.1580569	-0.9285341
O	0.4873378	5.6251882	-2.4563823
C	1.2853796	6.7988451	-2.6049594
H	2.1191750	6.8136741	-1.8695831
H	-2.5763687	8.2982064	0.4382832
H	-3.4003990	7.3088537	-0.8097059
H	-3.9045041	7.1778446	0.9157076
H	-2.4836703	5.2039007	0.2335379
H	-2.2959179	6.0736500	2.5923488
H	-0.9191293	7.1213055	2.0717400
H	0.3524090	5.1368941	1.5333185
H	-0.4682171	2.3679370	0.9617674
H	-0.4601650	1.7799614	2.6191990
H	1.7963461	2.8587365	3.0036224
H	1.6784632	3.7200014	1.4482075
H	4.0491081	2.6084423	1.2163223
H	3.8439546	1.3683659	2.4839949
H	4.1141833	0.8651054	0.7896498
H	2.3873409	2.9123503	-2.1562516
H	3.7526883	2.1261726	-1.2914909
H	0.8213561	2.4423017	-0.4183768
H	2.3805587	-0.0131620	-1.3273573
H	-1.8894621	0.4787221	-1.8270140
H	-1.1306099	0.4993923	-0.2135744
H	-0.9778516	1.9404948	-1.2862910
H	0.2280944	2.0509765	-4.8682504
H	-1.0969146	1.9955938	-3.6648993
H	0.4563688	2.6791274	-3.1995045
H	2.6128990	1.1243856	-3.3281225
H	2.3947557	-0.6524157	-3.4566614
H	0.3057202	-1.0644193	-4.9028707
H	-2.1453071	-1.4290276	-4.9057110
H	-2.3771543	-0.1686727	-3.6582875
H	-0.0326838	-2.9957673	-5.2527097
H	0.0537323	-4.6647279	-4.6645107

H	-1.5348755	-3.8841315	-4.9009609
H	-1.2775401	-6.2019966	-4.1386929
H	-2.0834915	-7.1410955	-2.8674759
H	-2.6826201	-5.5035802	-3.2775887
H	1.0510656	-5.9891235	-3.1427857
H	1.2489224	-5.8539628	-1.3750744
H	-1.0742647	-6.7821256	-0.2496931
H	-3.2220204	-5.7243048	0.5649351
H	-3.2929135	-4.7123963	-0.9098395
H	-0.5002493	-5.7595604	4.2541959
H	-0.3583039	-4.6201481	5.6166491
H	-1.6378883	-4.3797471	4.3806260
H	2.5548641	-3.8136735	3.2582056
H	2.1480633	-4.4895710	4.8764566
H	1.8457096	-5.4535619	3.3882264
H	1.2252416	-2.1682448	4.9017261
H	-1.6944589	-1.9042532	3.8948191
H	-1.1943306	-2.2290653	5.6050676
H	2.5841208	0.9882645	3.8837039
H	0.8500825	1.0643238	4.2718180
H	1.8385688	-0.3051761	4.8448824
H	2.9650151	3.6407806	-0.6644742
H	2.0775292	0.4043440	-4.8689110
H	0.4781699	-7.3231859	-2.0863405
H	-3.4546951	-6.4987359	-1.0323292
H	-1.7536326	0.2759981	-5.2772749
H	-0.8153583	-0.6668438	4.8103687
H	-0.7748362	-5.6492076	2.0118333
H	1.6992933	6.7968583	-3.6337855
H	0.6740468	7.7184513	-2.4643825
C	2.0316044	-2.7649035	-0.4616828
N	3.0507089	-3.2999473	-0.7224665
O	-1.7709409	-0.3397959	1.8974427
C	-2.9313033	0.0382339	1.6812824
H	-3.6950872	-0.6630677	1.2319608
N	-3.4322527	1.2609726	1.9304309
H	-2.8656048	2.0799221	2.2914828
H	-4.3980836	1.4518278	1.6605617

14

Co	0.4386333	-2.0591890	-0.0880640
N	0.1226459	-1.2983821	-1.7606387
N	-0.3695467	-3.6930923	-0.7182501
N	0.4686163	-2.5389459	1.7605664
N	1.1428566	-0.3837401	0.3343145
C	0.2866827	0.1812114	-1.8246248
C	-1.0000315	0.8161304	-1.2675394
C	0.5290201	0.4563031	-3.3549706
C	0.0371721	1.8413864	-3.7993074
C	2.0184861	0.2901775	-3.7311308
C	-0.2291697	-0.7546193	-4.0171641
C	-1.6767467	-0.5306681	-4.4926105
C	-0.1449735	-1.8227771	-2.9339699
C	-0.3552241	-3.2332962	-3.1401511
C	-0.4049775	-3.7392351	-4.5673870
C	-0.4928289	-4.0860385	-2.0524779
C	-0.6763577	-5.6190495	-2.1095666
C	-1.5942327	-6.2004945	-3.1976392
C	0.7475007	-6.2274546	-2.2148302
C	-1.2330789	-5.8936897	-0.6795616
C	-2.7683041	-5.8419262	-0.5408118
C	-0.6214317	-4.7518130	0.0926370
C	-0.3823456	-4.8048902	1.4641048
C	0.1386943	-3.7673960	2.2358654
C	0.4497317	-3.8957434	3.7227090
C	1.8908625	-4.4549408	3.8470495
C	-0.5246231	-4.7887707	4.5013782
C	0.4378539	-2.3952500	4.1319778
C	-0.9272562	-1.8680389	4.6162193
C	0.8111020	-1.7069301	2.8258721
C	1.3129284	-0.4191902	2.7405345
C	1.5951119	0.3465829	4.0142474
C	1.4807445	0.2171878	1.4545796
C	2.0709149	1.6147317	1.1924029
C	3.5864524	1.5833366	1.4876884
C	1.3667096	2.7543769	1.9803142
C	-0.1571364	2.5976218	1.9645055
C	-1.0577431	3.7831910	2.2576359

O	-2.2499372	3.5821289	2.5881643
N	-0.5644412	5.0289344	2.0485564
C	1.7750912	1.8208250	-0.3435159
C	2.8244499	2.6114531	-1.1228815
C	1.4766860	0.4102054	-0.8618506
C	-1.4763448	6.1444497	1.8200526
C	-1.9849465	6.1751025	0.3589149
C	-3.0071392	7.2848175	0.1373741
O	-0.9048587	6.4035125	-0.5570148
O	-0.6218521	3.8574580	-1.0952071
O	1.3503496	5.2512599	0.0248171
P	0.1523992	5.1591141	-0.9387774
O	0.6219016	5.6526715	-2.4426212
C	1.4153868	6.8338885	-2.5426784
H	2.2219579	6.8425752	-1.7772734
H	-2.5576446	8.2745965	0.3604466
H	-3.3347821	7.2890389	-0.9202891
H	-3.8950974	7.1449451	0.7868026
H	-2.4427033	5.1822868	0.1401407
H	-2.3376008	6.0435718	2.5095739
H	-0.9447777	7.0936973	2.0386476
H	0.3430616	5.1105618	1.5322979
H	-0.4758862	2.3327961	0.9348064
H	-0.5009565	1.7532603	2.5933943
H	1.7462594	2.8312506	3.0183228
H	1.6612838	3.6887793	1.4582320
H	4.0317692	2.5759925	1.2822442
H	3.7987154	1.3361616	2.5460655
H	4.1064008	0.8352220	0.8557158
H	2.4391150	2.8588484	-2.1311486
H	3.7839909	2.0667531	-1.2404347
H	0.8425706	2.4115098	-0.4213002
H	2.3724855	-0.0828652	-1.2953087
H	-1.8798549	0.4736222	-1.8434786
H	-1.1357512	0.4809256	-0.2229555
H	-0.9577023	1.9235368	-1.2927103
H	0.2919219	2.0199331	-4.8642941
H	-1.0547360	1.9673001	-3.6854493
H	0.4899537	2.6482259	-3.1901043

H	2.6479793	1.0801369	-3.2831455
H	2.4157477	-0.6958066	-3.4113840
H	0.3528367	-1.0853911	-4.9036901
H	-2.0965462	-1.4641819	-4.9176868
H	-2.3399139	-0.2099430	-3.6668654
H	0.0363213	-3.0040393	-5.2636843
H	0.1702424	-4.6750178	-4.6905536
H	-1.4390516	-3.9373352	-4.9177044
H	-1.1011142	-6.2570827	-4.1833961
H	-1.8713668	-7.2385315	-2.9214866
H	-2.5290583	-5.6193976	-3.3137476
H	1.2201913	-5.9678441	-3.1829907
H	1.4049988	-5.8391177	-1.4103186
H	-0.8680735	-6.8711694	-0.3011481
H	-3.0549241	-5.9093810	0.5274069
H	-3.1688761	-4.8868867	-0.9372946
H	-0.4003871	-5.8545157	4.2210224
H	-0.3252112	-4.7151310	5.5897538
H	-1.5805120	-4.5096896	4.3225872
H	2.6175950	-3.8055755	3.3176680
H	2.1886998	-4.5128421	4.9142257
H	1.9591737	-5.4711106	3.4094385
H	1.1993529	-2.2129570	4.9163455
H	-1.7030981	-2.0348203	3.8446011
H	-1.2327131	-2.3515471	5.5649799
H	2.4979675	0.9766003	3.9317551
H	0.7528163	1.0234558	4.2688331
H	1.7472004	-0.3266333	4.8761910
H	2.9966158	3.5886121	-0.6317199
H	2.1336838	0.3619672	-4.8324398
H	0.7002621	-7.3335412	-2.1390929
H	-3.2596399	-6.6770224	-1.0769317
H	-1.7133527	0.2449046	-5.2822475
H	-0.8822123	-0.7756405	4.7826719
H	-0.6228920	-5.7481345	1.9728970
H	1.8664943	6.8523011	-3.5556766
H	0.7926693	7.7469819	-2.4091400
O	2.1023603	-2.7210134	-0.5493380
H	2.6911860	-2.3929345	0.1621557

O	-1.7720902	-0.3787135	1.9082085
C	-2.9246727	-0.0131837	1.6385649
H	-3.6665520	-0.7286501	1.1751040
N	-3.4413695	1.2125097	1.8394241
H	-2.8943007	2.0412482	2.2069008
H	-4.3972892	1.3910757	1.5291265

15

Co	0.1170873	-1.9273467	-0.0129453
N	-0.1983177	-1.1788830	-1.6937866
N	-0.7713072	-3.5425778	-0.5952595
N	0.1799551	-2.3754153	1.8504865
N	0.9121108	-0.2734181	0.3633421
C	0.0110654	0.2937468	-1.7721778
C	-1.2204226	0.9837740	-1.1564180
C	0.1876232	0.5529236	-3.3137106
C	-0.2485202	1.9606525	-3.7440642
C	1.6461848	0.3123388	-3.7633567
C	-0.6679531	-0.6184410	-3.9300637
C	-2.1204778	-0.3075140	-4.3360300
C	-0.5873037	-1.6861581	-2.8440648
C	-0.9130429	-3.0817266	-3.0182195
C	-1.1174641	-3.5853326	-4.4349676
C	-1.0419637	-3.9196434	-1.9116246
C	-1.3838567	-5.4291246	-1.9426627
C	-2.4568269	-5.8970399	-2.9412980
C	-0.0609412	-6.2005211	-2.1834304
C	-1.8294870	-5.6559537	-0.4641657
C	-3.3371299	-5.4774050	-0.1910928
C	-1.0625617	-4.5682161	0.2494214
C	-0.7626636	-4.6140201	1.6099902
C	-0.1818909	-3.5819235	2.3534368
C	0.1709285	-3.6989082	3.8336191
C	1.5936909	-4.3080668	3.9275441
C	-0.8087831	-4.5452626	4.6566811
C	0.2236430	-2.1913780	4.2203790
C	-1.1026843	-1.6164469	4.7536849
C	0.5743954	-1.5340502	2.8908936
C	1.1263414	-0.2685776	2.7657107

C	1.4597550	0.5174122	4.0147620
C	1.2899433	0.3403409	1.4634654
C	1.9704721	1.6886724	1.1553957
C	3.4883858	1.5290503	1.3967177
C	1.4076941	2.9064826	1.9396013
C	-0.1233813	2.9046591	1.9908414
C	-0.8813869	4.1861362	2.2904360
O	-2.0607915	4.1256612	2.7085927
N	-0.2841906	5.3676654	1.9882588
C	1.6389303	1.8869491	-0.3733972
C	2.7146957	2.5884141	-1.2016273
C	1.2444876	0.4876228	-0.8540001
C	-1.1022409	6.5541002	1.7640591
C	-1.7395411	6.5538141	0.3537605
C	-2.6707625	7.7444664	0.1513305
O	-0.7346691	6.6249717	-0.6682368
O	-0.7192010	4.0376898	-1.0658184
O	1.4524482	5.3073166	-0.1872187
P	0.1759194	5.2686254	-1.0465314
O	0.5634428	5.6327047	-2.6122372
C	1.4556506	6.7204156	-2.8446526
H	2.3255315	6.6824220	-2.1529823
H	-2.1124597	8.6960646	0.2714996
H	-3.0923356	7.7230250	-0.8724282
H	-3.5048396	7.7276191	0.8821256
H	-2.3047876	5.5998854	0.2399739
H	-1.9032426	6.5812405	2.5293080
H	-0.4650339	7.4559000	1.8733990
H	0.5810559	5.3342722	1.3994606
H	-0.5146344	2.6525923	0.9819966
H	-0.5191790	2.1158268	2.6596902
H	1.8348124	2.9661310	2.9602820
H	1.7714830	3.7970615	1.3868045
H	4.0160932	2.4725424	1.1565244
H	3.7109009	1.2804354	2.4529292
H	3.9136655	0.7228134	0.7640570
H	2.3138693	2.8486853	-2.2008607
H	3.6256481	1.9695180	-1.3418734
H	0.7421044	2.5327688	-0.4340728

H	2.1078380	-0.0377550	-1.3181293
H	-2.1438212	0.6687521	-1.6767002
H	-1.3086625	0.6655457	-0.1012701
H	-1.1368522	2.0887289	-1.1954651
H	-0.0559867	2.1133096	-4.8263390
H	-1.3203605	2.1534764	-3.5589967
H	0.2908446	2.7451844	-3.1786572
H	2.3368779	1.0676750	-3.3460921
H	2.0089467	-0.6933077	-3.4619903
H	-0.1517716	-0.9821852	-4.8450037
H	-2.6170695	-1.2142108	-4.7351286
H	-2.7210913	0.0541714	-3.4800040
H	-0.6534026	-2.9007493	-5.1683693
H	-0.6559892	-4.5781086	-4.5952509
H	-2.1879042	-3.6761191	-4.7140205
H	-2.0662579	-5.9926027	-3.9693740
H	-2.8195376	-6.9022795	-2.6439156
H	-3.3282852	-5.2154490	-2.9686160
H	0.3687224	-5.9589238	-3.1772819
H	0.6925450	-5.9429939	-1.4114986
H	-1.5180518	-6.6632166	-0.1151749
H	-3.5305610	-5.5248664	0.8987234
H	-3.6873463	-4.4886992	-0.5505185
H	-0.7336627	-5.6180585	4.3855176
H	-0.5719805	-4.4662499	5.7373269
H	-1.8586028	-4.2288709	4.5079074
H	2.3279883	-3.6927840	3.3679763
H	1.9220217	-4.3623989	4.9863835
H	1.6129240	-5.3317849	3.5023739
H	1.0203954	-2.0253251	4.9736024
H	-1.9193646	-1.7796779	4.0248256
H	-1.3744207	-2.0682285	5.7283781
H	2.3994081	1.0894487	3.9115322
H	0.6594187	1.2496310	4.2494154
H	1.5744652	-0.1361342	4.8974894
H	2.9780315	3.5576762	-0.7371058
H	1.7122846	0.3782084	-4.8692345
H	-0.2377967	-7.2955653	-2.1462426
H	-3.9452262	-6.2638445	-0.6793639

H	-2.1505253	0.4677513	-5.1263578
H	-1.0266584	-0.5209875	4.8808279
H	-1.0376795	-5.5331726	2.1454739
H	1.8121273	6.6467409	-3.8925579
H	0.9403034	7.6980666	-2.7085151
N	2.0028384	-2.7195447	-0.4492508
C	3.1258619	-2.5527626	0.3349992
C	2.3919745	-3.2946706	-1.5821543
C	4.2282918	-3.0464741	-0.3414051
N	3.7416830	-3.5125968	-1.5521684
H	1.7311770	-3.5475251	-2.4196397
H	5.2882552	-3.1046102	-0.0763334
H	4.2967406	-3.9338415	-2.2983257
H	3.0572598	-2.0772084	1.3202675
O	-1.9685107	0.1507092	2.3577855
C	-2.9749137	0.6019174	1.7989623
H	-3.6431895	-0.0646670	1.1771051
N	-3.4059304	1.8780691	1.8400406
H	-2.8618901	2.6604112	2.2956106
H	-4.2065573	2.1392979	1.2637780

17

Co	0.6441399	-1.6768157	0.0728502
N	0.8337222	-0.8875318	-1.5934118
N	-0.1117050	-3.2273960	-0.7752053
N	0.2259664	-2.2071300	1.8546970
N	1.3311146	-0.0654654	0.6969385
C	1.0059902	0.5993504	-1.6039207
C	-0.3630749	1.2594424	-1.4194935
C	1.6523474	0.8730496	-3.0197802
C	1.3058036	2.2389778	-3.6232440
C	3.1886783	0.7262672	-2.9739338
C	1.0925934	-0.3322725	-3.8629252
C	-0.1726273	-0.0810712	-4.7074747
C	0.8548372	-1.3970812	-2.8064968
C	0.6027462	-2.7875711	-3.0892240
C	0.9199827	-3.2961411	-4.4814148
C	0.0607749	-3.6078434	-2.1091187
C	-0.3630199	-5.0828106	-2.2764783

C	-1.0486476	-5.4746398	-3.5961452
C	0.8928546	-5.9607565	-2.0398727
C	-1.3211212	-5.2428290	-1.0535343
C	-2.8032792	-4.9144129	-1.3224027
C	-0.7502869	-4.2223278	-0.1017271
C	-0.8794247	-4.3059889	1.2824163
C	-0.3735469	-3.3813321	2.1956970
C	-0.3326447	-3.6197273	3.7027820
C	0.9475509	-4.4426308	4.0017496
C	-1.5620693	-4.3439188	4.2657759
C	-0.1366881	-2.1617879	4.2023985
C	-1.4538465	-1.4022518	4.4714300
C	0.5517074	-1.5113796	3.0136519
C	1.2693082	-0.3312361	3.0956432
C	1.6907596	0.1493013	4.4706365
C	1.5543805	0.4215766	1.9057632
C	2.1435704	1.8409478	1.8414141
C	3.6606793	1.7538813	2.1376917
C	1.4715462	2.9143872	2.7506105
C	0.0276603	2.6786660	3.2473481
C	-1.0627425	2.2190291	2.2611708
O	-1.5200272	1.0591195	2.3187248
N	-1.5118296	3.1820560	1.4282869
C	1.8792297	2.2106717	0.3383751
C	2.8119742	3.2559290	-0.2619310
C	1.8630302	0.8434316	-0.3467670
C	-2.6939518	3.0693722	0.5902677
C	-2.5551394	3.8648392	-0.7260737
C	-3.9095670	4.0607121	-1.3995463
O	-1.9758706	5.1530992	-0.4866445
O	0.2217266	4.4277946	-1.7242130
O	0.2568065	5.1925618	0.8299902
P	-0.2949249	5.3155283	-0.6041006
O	-0.2222793	6.8870993	-1.0941921
C	-0.6006042	7.8959098	-0.1610358
H	-0.1440777	7.7129842	0.8362595
H	-4.5757379	4.6738776	-0.7580809
H	-3.7793335	4.5877470	-2.3646685
H	-4.3995243	3.0828125	-1.5863948

H	-1.8911853	3.3063395	-1.4165960
H	-2.8931281	2.0018565	0.3624634
H	-3.5755959	3.4611688	1.1517785
H	-0.9585407	4.0777740	1.3830595
H	0.0044520	1.9250295	4.0537998
H	-0.3106844	3.6348648	3.6985682
H	2.0982625	3.0893247	3.6519735
H	1.4807156	3.8657781	2.1776120
H	4.1192194	2.7599853	2.0710477
H	3.8557687	1.3608302	3.1546793
H	4.1833984	1.0925500	1.4161814
H	2.4081300	3.5977320	-1.2316251
H	3.8487608	2.8822767	-0.3901973
H	0.8550193	2.6288391	0.2984519
H	2.8998268	0.4963250	-0.5488197
H	-1.0644644	0.9627084	-2.2174543
H	-0.8152072	0.9579071	-0.4546147
H	-0.2370550	2.3597709	-1.4459414
H	1.7765522	2.3387823	-4.6236144
H	0.2204488	2.4056406	-3.7366421
H	1.6532046	3.0695743	-2.9838316
H	3.6533677	1.5344640	-2.3778241
H	3.5058530	-0.2501752	-2.5502967
H	1.8877207	-0.6720220	-4.5610280
H	-0.4860731	-1.0053482	-5.2315318
H	-1.0217529	0.2521954	-4.0818424
H	1.6687347	-2.6491347	-4.9738146
H	1.3482883	-4.3149657	-4.4576892
H	0.0322693	-3.3273591	-5.1462655
H	-0.3290650	-5.6173857	-4.4213318
H	-1.5729489	-6.4431327	-3.4625325
H	-1.7956085	-4.7236601	-3.9169915
H	1.6635066	-5.7743249	-2.8136038
H	1.3491719	-5.7471412	-1.0515483
H	-1.2476471	-6.2676567	-0.6331734
H	-3.3710912	-4.9203405	-0.3712740
H	-2.9081187	-3.9039521	-1.7642045
H	-1.5871579	-5.4024209	3.9362641
H	-1.5283037	-4.3499887	5.3739720

H	-2.5090831	-3.8667252	3.9505123
H	1.8525979	-3.9199259	3.6294766
H	1.0592083	-4.5931670	5.0955632
H	0.9009393	-5.4372175	3.5149301
H	0.4965224	-2.1451012	5.1111383
H	-2.1338270	-1.4858089	3.6010172
H	-1.9669352	-1.7944838	5.3712784
H	2.2033509	1.1235431	4.4401886
H	0.8308872	0.2570603	5.1622127
H	2.3918313	-0.5755391	4.9358678
H	2.8256460	4.1591374	0.3786547
H	3.6011526	0.7954166	-4.0012893
H	0.6232965	-7.0366955	-2.0732789
H	-3.2734878	-5.6486320	-2.0049235
H	0.0147476	0.6935058	-5.4749058
H	-1.2732377	-0.3180967	4.5950541
H	-1.3812408	-5.1969825	1.6829174
H	-0.2470385	8.8700115	-0.5565754
H	-1.7068569	7.9374377	-0.0394094
C	2.3944282	-2.5423567	0.0948359
H	2.2600140	-3.5485800	0.5401657
H	2.7497423	-2.6218902	-0.9520074
H	3.0870757	-1.9342330	0.7081345
C	-2.8988484	-1.0743477	0.4239440
H	-1.9276269	-1.6224331	0.3494567
H	-2.8273339	-0.3985271	1.2962920
H	-3.7031010	-1.8391197	0.5637858
O	-3.1250857	-0.2709051	-0.7185752
C	-3.0037431	-0.9883448	-1.9210989
H	-3.7600921	-1.8108037	-2.0025748
H	-3.1714796	-0.2833597	-2.7595788
H	-1.9882577	-1.4452255	-2.0347929

18

Co	0.4798009	-1.9742782	-0.0607047
N	0.1905042	-1.2759147	-1.7619222
N	-0.3861410	-3.5954019	-0.6333927
N	0.4081144	-2.3407863	1.8097999
N	1.2292262	-0.2939260	0.2890971

C	0.3053433	0.2105432	-1.8538296
C	-0.9843539	0.8065574	-1.2624347
C	0.4808443	0.4660613	-3.3957911
C	-0.0822683	1.8242362	-3.8401376
C	1.9597891	0.3541299	-3.8270405
C	-0.2569458	-0.7836460	-4.0121265
C	-1.7232187	-0.6143280	-4.4536148
C	-0.1179862	-1.8274100	-2.9132672
C	-0.3358136	-3.2432765	-3.0727021
C	-0.3678738	-3.8001150	-4.4806930
C	-0.5048648	-4.0468830	-1.9548291
C	-0.7501827	-5.5691444	-1.9374701
C	-1.6523531	-6.1678464	-3.0285881
C	0.6487716	-6.2423217	-1.9595362
C	-1.3732910	-5.7390204	-0.5169533
C	-2.9077165	-5.5958521	-0.4433258
C	-0.7343449	-4.5913909	0.2218350
C	-0.5786476	-4.5630977	1.6055317
C	-0.0287770	-3.5133548	2.3412486
C	0.2214819	-3.5826486	3.8412152
C	1.5843002	-4.2987926	4.0379381
C	-0.8762384	-4.3079203	4.6298761
C	0.3804017	-2.0709458	4.1712131
C	-0.9095800	-1.3512713	4.6170618
C	0.8269179	-1.4928347	2.8367526
C	1.4479696	-0.2641220	2.6930353
C	1.8824738	0.4866904	3.9328541
C	1.6039207	0.3294760	1.3851668
C	2.1803767	1.7219891	1.0759749
C	3.7130906	1.6827431	1.2664918
C	1.5287199	2.8665256	1.9018360
C	0.0093650	2.6981529	2.0257428
C	-0.8728808	3.8798906	2.4221267
O	-1.9727451	3.6739071	2.9593216
N	-0.4355829	5.1216181	2.0570286
C	1.7859007	1.9133210	-0.4385013
C	2.7668869	2.7255119	-1.2820844
C	1.5126633	0.4906350	-0.9323269
C	-1.3946605	6.2029358	1.8777330

C	-2.0478996	6.1581680	0.4762357
C	-3.1100853	7.2383374	0.3050190
O	-1.0668093	6.3615461	-0.5555383
O	-0.8133704	3.8007186	-1.0275548
O	1.2552834	5.2522593	-0.1895436
P	-0.0436322	5.1157153	-1.0031897
O	0.2507227	5.5455123	-2.5727367
C	1.0035287	6.7337513	-2.8093204
H	1.8969178	6.7816889	-2.1491698
H	-2.6625955	8.2454063	0.4363178
H	-3.5422980	7.1863096	-0.7132801
H	-3.9255659	7.1135717	1.0460970
H	-2.5018086	5.1485443	0.3471412
H	-2.1802692	6.1042996	2.6533205
H	-0.8778187	7.1771421	2.0052339
H	0.3920622	5.1809985	1.4231076
H	-0.4028250	2.4310356	1.0287034
H	-0.2599383	1.8659040	2.7049464
H	1.9996395	2.9690044	2.8997507
H	1.7619006	3.7946294	1.3391653
H	4.1442613	2.6726371	1.0219587
H	3.9969163	1.4458254	2.3099869
H	4.1856053	0.9247320	0.6099082
H	2.3153330	2.9610837	-2.2654474
H	3.7292534	2.2011734	-1.4552774
H	0.8288898	2.4694649	-0.4592932
H	2.4175437	0.0306391	-1.3829178
H	-1.8690088	0.4336857	-1.8119093
H	-1.0841365	0.4845801	-0.2076917
H	-0.9787354	1.9169550	-1.2916766
H	0.1189873	1.9918394	-4.9180324
H	-1.1716377	1.9142110	-3.6791106
H	0.3691872	2.6583960	-3.2679248
H	2.5731395	1.1735417	-3.4111691
H	2.4132344	-0.6110639	-3.5181103
H	0.3118635	-1.1185588	-4.9055323
H	-2.1236848	-1.5672128	-4.8534454
H	-2.3752895	-0.2991742	-3.6168735
H	0.1026694	-3.0978488	-5.1920599

H	0.1932859	-4.7492651	-4.5537074
H	-1.3977367	-3.9916994	-4.8456068
H	-1.1256737	-6.2924594	-3.9904532
H	-1.9785883	-7.1794624	-2.7122438
H	-2.5587554	-5.5589351	-3.2087365
H	1.1638661	-6.0527346	-2.9220401
H	1.3025805	-5.8472212	-1.1554378
H	-1.0762605	-6.7119505	-0.0727333
H	-3.2393888	-5.5918703	0.6138857
H	-3.2385876	-4.6420864	-0.9031084
H	-0.8765339	-5.3943058	4.4080180
H	-0.6977520	-4.2010610	5.7188413
H	-1.8840178	-3.9109072	4.4026133
H	2.3935469	-3.7777116	3.4869209
H	1.8498290	-4.3201320	5.1147770
H	1.5388690	-5.3425714	3.6682499
H	1.1574892	-1.9376242	4.9496063
H	-1.6856096	-1.4030056	3.8274586
H	-1.3025120	-1.7759780	5.5609092
H	2.8157784	1.0503977	3.7629792
H	1.1177781	1.2218414	4.2588860
H	2.0657391	-0.1973023	4.7805237
H	2.9425422	3.7089623	-0.8066634
H	2.0294191	0.4191139	-4.9324154
H	0.5457620	-7.3394101	-1.8323356
H	-3.4238413	-6.4294291	-0.9574543
H	-1.8026363	0.1461164	-5.2542910
H	-0.7080208	-0.2764279	4.7888563
H	-0.9031325	-5.4548338	2.1581732
H	1.3271560	6.7225351	-3.8701360
H	0.3847144	7.6418469	-2.6313442
C	2.0949560	-2.7399331	-0.3685644
N	3.1189961	-3.2875386	-0.5783053
C	-3.0581609	0.7348176	1.7299631
H	-4.0590469	0.5089103	2.1703360
H	-3.2123987	1.1650052	0.7126372
H	-2.5530829	1.4988350	2.3521084
O	-2.2466771	-0.4385688	1.6783509
C	-2.8426033	-1.4730922	0.9248301

H	-2.9380443	-1.2030883	-0.1553426
H	-2.1937581	-2.3666562	1.0034225
H	-3.8597391	-1.7394173	1.3046586

19

Co	0.5181267	-2.0063394	-0.0750522
N	0.2036755	-1.2874151	-1.7608372
N	-0.2847607	-3.6507365	-0.6734525
N	0.4796441	-2.4185492	1.7935363
N	1.2105280	-0.3174583	0.3143533
C	0.3179325	0.1962757	-1.8403668
C	-0.9776865	0.7939347	-1.2652347
C	0.5247159	0.4643025	-3.3771543
C	-0.0306188	1.8242439	-3.8247566
C	2.0125762	0.3544645	-3.7780887
C	-0.1950107	-0.7831249	-4.0169525
C	-1.6579073	-0.6205606	-4.4711621
C	-0.0584996	-1.8347878	-2.9238384
C	-0.2364979	-3.2543320	-3.1039044
C	-0.2514151	-3.7897743	-4.5204649
C	-0.3898062	-4.0804802	-1.9989775
C	-0.6096972	-5.6087311	-2.0133779
C	-1.5118251	-6.1963200	-3.1111403
C	0.7958523	-6.2643834	-2.0586407
C	-1.2176499	-5.8200330	-0.5920026
C	-2.7541055	-5.7132404	-0.5040568
C	-0.5996982	-4.6700454	0.1630047
C	-0.4379952	-4.6656578	1.5482003
C	0.0776610	-3.6131255	2.3037624
C	0.3066635	-3.6934389	3.8081222
C	1.6901588	-4.3595587	4.0269793
C	-0.7762958	-4.4698272	4.5684540
C	0.4002804	-2.1808621	4.1595581
C	-0.9232580	-1.5184810	4.5948459
C	0.8438045	-1.5684228	2.8383480
C	1.4141875	-0.3113467	2.7191421
C	1.7892548	0.4479728	3.9736256
C	1.5722830	0.2997387	1.4188202
C	2.1475716	1.6963630	1.1259456

C	3.6751850	1.6679036	1.3533025
C	1.4725960	2.8354147	1.9404174
C	-0.0479878	2.6583118	2.0282659
C	-0.9458439	3.8335018	2.4071786
O	-2.0613242	3.6192002	2.9073448
N	-0.5005873	5.0808772	2.0698688
C	1.7867581	1.8894447	-0.3970808
C	2.7870815	2.7020446	-1.2169731
C	1.5132738	0.4681019	-0.8974892
C	-1.4565732	6.1617272	1.8722902
C	-2.0601958	6.1355973	0.4483110
C	-3.1172508	7.2171561	0.2548092
O	-1.0434910	6.3543481	-0.5445782
O	-0.7725954	3.7991106	-1.0405623
O	1.2675904	5.2422090	-0.1228040
P	-0.0061367	5.1140437	-0.9768375
O	0.3342611	5.5651592	-2.5313530
C	1.0893482	6.7585887	-2.7293781
H	1.9601847	6.8026212	-2.0392850
H	-2.6756370	8.2226500	0.4151297
H	-3.5133455	7.1785859	-0.7786480
H	-3.9580107	7.0822858	0.9652399
H	-2.5079234	5.1273986	0.2893501
H	-2.2682150	6.0495262	2.6186851
H	-0.9480465	7.1357802	2.0317027
H	0.3466845	5.1494189	1.4641212
H	-0.4343108	2.3924783	1.0209548
H	-0.3282148	1.8206872	2.6962266
H	1.9192304	2.9355679	2.9498491
H	1.7146284	3.7669619	1.3869996
H	4.1070254	2.6614144	1.1248440
H	3.9355546	1.4257971	2.4019776
H	4.1696924	0.9194079	0.7011308
H	2.3565799	2.9434109	-2.2082655
H	3.7514398	2.1756133	-1.3734203
H	0.8327937	2.4499262	-0.4376329
H	2.4158468	-0.0095591	-1.3350559
H	-1.8551283	0.4307785	-1.8326731
H	-1.0942059	0.4583257	-0.2167142

H	-0.9677893	1.9038144	-1.2847189
H	0.1921362	1.9994691	-4.8973342
H	-1.1234476	1.9103381	-3.6858131
H	0.4060940	2.6556344	-3.2371140
H	2.6184872	1.1693464	-3.3433392
H	2.4541985	-0.6147952	-3.4646913
H	0.3859162	-1.1014103	-4.9086527
H	-2.0464977	-1.5726581	-4.8847043
H	-2.3204281	-0.3209179	-3.6369176
H	0.2206132	-3.0732741	-5.2166813
H	0.3149903	-4.7348158	-4.6053959
H	-1.2768986	-3.9813840	-4.8988618
H	-0.9909648	-6.2938364	-4.0793846
H	-1.8250153	-7.2181869	-2.8146974
H	-2.4260509	-5.5937869	-3.2724175
H	1.3093442	-6.0380357	-3.0142567
H	1.4387885	-5.8864956	-1.2378483
H	-0.8961600	-6.7950357	-0.1697255
H	-3.0766958	-5.7372637	0.5557339
H	-3.1090058	-4.7577899	-0.9416986
H	-0.7318134	-5.5524861	4.3328935
H	-0.6229647	-4.3705175	5.6621179
H	-1.7938580	-4.1084631	4.3261969
H	2.4906717	-3.7963121	3.5049745
H	1.9338373	-4.3894196	5.1088845
H	1.6950745	-5.3977825	3.6387968
H	1.1588514	-2.0276597	4.9527333
H	-1.6833316	-1.5856830	3.7914122
H	-1.3167471	-1.9729921	5.5246736
H	2.7152891	1.0334557	3.8380917
H	0.9978059	1.1666940	4.2711256
H	1.9567687	-0.2308983	4.8286086
H	2.9564306	3.6829158	-0.7335666
H	2.1055820	0.4268967	-4.8813988
H	0.7078103	-7.3664305	-1.9658927
H	-3.2573025	-6.5472112	-1.0305916
H	-1.7374083	0.1480464	-5.2642601
H	-0.7654277	-0.4398668	4.7866599
H	-0.7380837	-5.5765622	2.0835338

H	1.4484061	6.7608876	-3.7788729
H	0.4613909	7.6625113	-2.5619500
O	2.1994958	-2.6571118	-0.4783193
H	2.7790710	-2.2426786	0.1948847
C	-3.0737583	0.6137016	1.7005393
H	-3.2492298	1.0266083	0.6794141
H	-2.5977246	1.4048420	2.3118876
H	-4.0636887	0.3522955	2.1464546
O	-2.2159084	-0.5258928	1.6589980
C	-2.7581096	-1.5809758	0.8931409
H	-2.0848630	-2.4540953	0.9943785
H	-3.7769661	-1.8774339	1.2450465
H	-2.8300445	-1.3179427	-0.1906134

20

Co	0.3127679	-1.5712402	0.1199398
N	0.6648429	-0.7456338	-1.5154925
N	-0.4793565	-3.0589564	-0.8209088
N	-0.2998660	-2.1086134	1.8613559
N	1.0142175	0.0070082	0.8413758
C	0.9398889	0.7201264	-1.4679999
C	-0.3933925	1.4725604	-1.3799992
C	1.7190797	0.9740173	-2.8196185
C	1.5487221	2.3809014	-3.4030830
C	3.2261733	0.6825464	-2.6517693
C	1.1218803	-0.1519278	-3.7435988
C	-0.0367187	0.2429045	-4.6806924
C	0.7090136	-1.2176699	-2.7408562
C	0.3543003	-2.5725438	-3.0881432
C	0.6789972	-3.0550660	-4.4890986
C	-0.3048416	-3.3778687	-2.1665864
C	-0.8713292	-4.7912699	-2.4290636
C	-1.5439834	-5.0389610	-3.7897575
C	0.2724130	-5.8120523	-2.2006526
C	-1.8875693	-4.9066870	-1.2498535
C	-3.3033558	-4.3689935	-1.5493993
C	-1.2425614	-4.0106272	-0.2223699
C	-1.4607139	-4.1338640	1.1506994
C	-0.9855080	-3.2544469	2.1246041

C	-1.1138744	-3.4966325	3.6275243
C	0.0965941	-4.3617327	4.0631026
C	-2.4204494	-4.1775840	4.0536947
C	-0.9251193	-2.0451578	4.1506438
C	-2.2344711	-1.2346338	4.2674974
C	-0.0863619	-1.4190372	3.0471585
C	0.6581996	-0.2622671	3.2151872
C	0.9240612	0.2066120	4.6343256
C	1.1160619	0.4799396	2.0685680
C	1.7987989	1.8571915	2.0905240
C	3.2516283	1.6723838	2.5907946
C	1.0697303	2.9641131	2.9102034
C	-0.4424531	2.7990610	3.1795306
C	-1.4039289	2.5129963	2.0123684
O	-1.9139985	1.3847430	1.8624811
N	-1.6959300	3.5950173	1.2526420
C	1.7655945	2.2439504	0.5679285
C	2.8699752	3.1870802	0.1042400
C	1.7020040	0.8836496	-0.1332522
C	-2.7487521	3.6475020	0.2558850
C	-2.3273664	4.4131541	-1.0166241
C	-3.5322556	4.7523908	-1.8890582
O	-1.6644141	5.6312748	-0.6691379
O	0.6049630	4.6412802	-1.5194687
O	0.3076020	5.4515321	1.0076890
P	0.0227953	5.6027159	-0.4986898
O	0.3545259	7.1468728	-0.9712695
C	-0.0424244	8.2099780	-0.1098524
H	0.2214491	7.9908921	0.9479102
H	-4.2246646	5.4299088	-1.3478003
H	-3.1963627	5.2655751	-2.8106408
H	-4.0893176	3.8351557	-2.1746216
H	-1.6248730	3.7832138	-1.6016316
H	-3.0534534	2.6108579	0.0017971
H	-3.6404990	4.1568631	0.6928311
H	-1.0737724	4.4398920	1.3521061
H	-0.6292988	1.9801188	3.8953402
H	-0.7813895	3.7360676	3.6689771
H	1.5615583	3.0861232	3.8996692

H	1.2172198	3.9244016	2.3705763
H	3.7849451	2.6431460	2.5950084
H	3.2776336	1.2682712	3.6217191
H	3.8173074	0.9727010	1.9413219
H	2.6423891	3.5564400	-0.9111124
H	3.8721791	2.7108113	0.1182279
H	0.8046291	2.7665107	0.3978534
H	2.7218182	0.4563027	-0.2525467
H	-1.0479170	1.2151513	-2.2291025
H	-0.9222316	1.2004398	-0.4456695
H	-0.1953754	2.5609502	-1.3839673
H	2.1183102	2.4666306	-4.3519211
H	0.4961754	2.6400738	-3.6133324
H	1.8970818	3.1633118	-2.7057638
H	3.7118384	1.4311835	-1.9970398
H	3.4085703	-0.3272811	-2.2255544
H	1.9381953	-0.5459876	-4.3886064
H	-0.4043590	-0.6382118	-5.2414968
H	-0.8932284	0.6600859	-4.1189884
H	1.5128828	-2.4719173	-4.9222693
H	0.9945840	-4.1149375	-4.4984220
H	-0.1741185	-2.9644170	-5.1933394
H	-0.8140799	-5.2237181	-4.5977464
H	-2.1802113	-5.9453782	-3.7235670
H	-2.1875355	-4.1912086	-4.0925664
H	1.0856697	-5.6755209	-2.9425626
H	0.7095256	-5.6976983	-1.1873066
H	-1.9555627	-5.9558528	-0.8918932
H	-3.9099906	-4.3822776	-0.6221524
H	-3.2490332	-3.3167283	-1.8978360
H	-2.4469446	-5.2337174	3.7167928
H	-2.5071630	-4.1863661	5.1593892
H	-3.3112616	-3.6679875	3.6396369
H	1.0535267	-3.8731115	3.7861515
H	0.0880580	-4.5134678	5.1626114
H	0.0669778	-5.3547447	3.5719060
H	-0.4010018	-2.0496484	5.1268162
H	-2.7898343	-1.2556447	3.3094020
H	-2.8855086	-1.6265227	5.0739301

H	1.4648192	1.1651169	4.6695332
H	-0.0086460	0.3429413	5.2187211
H	1.5404558	-0.5357086	5.1851834
H	2.8878638	4.0910010	0.7443595
H	3.7298594	0.7293194	-3.6388025
H	-0.1074267	-6.8502650	-2.2994921
H	-3.8288164	-4.9829879	-2.3069804
H	0.2911555	0.9997594	-5.4186732
H	-2.0230348	-0.1673029	4.4698613
H	-2.0577714	-4.9934991	1.4833532
H	0.4862823	9.1274170	-0.4408172
H	-1.1403940	8.3883732	-0.1689065
N	2.1817006	-2.5054070	0.2870214
C	3.0114542	-2.4311471	1.3874799
C	2.8427613	-3.1590117	-0.6628568
C	4.2098369	-3.0602131	1.0960224
N	4.0818104	-3.5146203	-0.2067156
H	2.4547152	-3.3754945	-1.6652117
H	5.1187091	-3.2192100	1.6840999
H	4.7926459	-4.0195823	-0.7376252
H	2.6859728	-1.9234091	2.3026082
C	-3.4756443	-0.0962340	-2.3856149
H	-3.3334730	0.9661780	-2.0685700
H	-3.1243795	-0.2012555	-3.4314487
H	-4.5727457	-0.3139194	-2.3538387
O	-2.7317992	-0.9913920	-1.5906120
C	-3.0658980	-0.8938362	-0.2110194
H	-2.4673569	-1.6522427	0.3257601
H	-2.8133847	0.1015219	0.2217217
H	-4.1494322	-1.1057243	-0.0351056

Cartesian coordinates of the non-covalent minima for the DMB relaxed scan

Cbl

Co	0.4054998	0.6428721	-0.0794981
N	1.0484081	0.4427806	-1.8150764
N	1.9675093	-0.2535580	0.6050403
N	-0.4894853	0.7824306	1.6023152
N	-0.9639982	1.6158822	-0.9134136
C	0.0618215	0.7147087	-2.9035619

C	-0.8735061	-0.5011909	-3.0342347
C	0.9863692	0.9373291	-4.1637019
C	0.3262811	0.5703321	-5.4990321
C	1.4967266	2.3932665	-4.2465016
C	2.2246242	0.0328883	-3.8095130
C	2.2487928	-1.4081675	-4.3587054
C	2.2165065	0.0481608	-2.2881381
C	3.3142617	-0.3730552	-1.4610134
C	4.6562409	-0.6037680	-2.1294117
C	3.1375451	-0.5588962	-0.0917653
C	4.2115648	-1.0448789	0.9082154
C	5.1739431	-2.1516940	0.4447320
C	5.0160083	0.1991010	1.3690381
C	3.3100319	-1.5235194	2.0879839
C	2.8761930	-3.0041635	2.0428740
C	2.0895284	-0.6596027	1.9029191
C	1.1924536	-0.3849193	2.9277931
C	-0.0121782	0.3063017	2.7831277
C	-0.9325009	0.6537183	3.9476906
C	-0.4490725	2.0012589	4.5441340
C	-0.9907262	-0.4136099	5.0488052
C	-2.2648636	0.8697022	3.1792967
C	-3.1096583	-0.4080892	3.0133909
C	-1.7767897	1.2813636	1.8008756
C	-2.5660574	1.9492014	0.8747104
C	-3.8977261	2.4954615	1.3551222
C	-2.1370922	2.0627385	-0.4958455
C	-2.9464823	2.6938936	-1.6467230
C	-2.9725223	4.2287574	-1.4758289
C	-4.3818155	2.1114696	-1.7813783
C	-4.4750437	0.6040480	-1.4997912
C	-5.8619277	0.0505848	-1.8423375
O	-6.2770474	-0.0075141	-3.0021130
N	-6.5617389	-0.3579191	-0.7420834
C	-2.1135923	2.2555909	-2.9059907
C	-2.1290529	3.2272462	-4.0847176
C	-0.7270136	1.9244597	-2.3417156
C	-7.9355332	-0.8411487	-0.7999525
C	-8.6779656	-0.5799915	0.5205655

C	-10.1591787	-0.9242816	0.4091756
O	-8.1536828	-1.3884956	1.6013730
O	-6.8454270	-1.5073599	3.8063097
O	-6.2751661	0.3846178	1.9481461
P	-6.7400871	-1.0050591	2.3819884
O	-5.6507000	-2.0206015	1.5585629
C	-5.6466464	-3.4081619	1.8528785
C	-5.4471238	-4.1860867	0.5063556
O	-5.7157372	-3.4321755	-0.6372577
C	-3.9915108	-4.7378501	0.6597589
O	-3.8704967	-4.9747299	2.0471507
C	-4.4183787	-3.8074806	2.7026726
C	-4.6638203	-4.0554668	4.1926480
O	-4.7955695	-2.8473710	4.8973276
N	-2.9634057	-3.8201357	0.1600032
C	-1.6656257	-4.1800000	-0.2103188
C	-3.0062625	-2.4333416	0.1222675
N	-1.8613218	-1.8860296	-0.2412879
C	-0.9958894	-2.9504239	-0.4644260
C	0.3435035	-2.9563321	-0.8929067
C	1.0040169	-4.1815341	-1.0763552
C	2.4325958	-4.1910817	-1.5722799
C	0.3254719	-5.4162501	-0.7997697
C	1.0558285	-6.7266721	-0.9835352
C	-1.0119755	-5.4135913	-0.3585894
H	-5.8052081	-2.4928199	-0.3404689
H	-5.5969001	-2.3509419	4.5468282
H	-3.7807592	-4.6004192	4.5933005
H	-5.5442095	-4.7388513	4.3116164
H	-3.6883923	-2.9709015	2.6376839
H	-6.5979253	-3.6960388	2.3462788
H	-6.0975631	-5.0874192	0.5191788
H	-3.8401592	-5.7027558	0.1386105
H	-3.9078664	-1.8784738	0.4072359
H	1.9612808	-6.7856920	-0.3418610
H	0.4123286	-7.5924557	-0.7348414
H	1.4064315	-6.8578840	-2.0299351
H	2.8114742	-3.1574668	-1.6960916
H	3.1149496	-4.7247145	-0.8760163

H	2.5253650	-4.7067748	-2.5523025
H	-1.5210465	-6.3632481	-0.1355580
H	0.8611122	-2.0045633	-1.0693720
H	-10.6577656	-0.2885115	-0.3494986
H	-10.2867156	-1.9873054	0.1201292
H	-10.6545201	-0.7722723	1.3871997
H	-8.4596001	-0.3435111	-1.6446440
H	-7.9516682	-1.9354899	-1.0062883
H	-6.2430768	-0.0180044	0.1925811
H	-3.7470197	0.0438734	-2.1216356
H	-4.2155592	0.3893220	-0.4450685
H	-5.0919113	2.6476967	-1.1210795
H	-4.7315225	2.3026236	-2.8195154
H	-1.9493433	4.6562773	-1.4932808
H	-3.5597346	4.7029511	-2.2869366
H	-3.4368999	4.5187590	-0.5145723
H	-1.6491247	2.7752914	-4.9746453
H	-1.5955855	4.1730668	-3.8612756
H	-2.5649312	1.3054158	-3.2496557
H	-0.0707275	2.8202530	-2.3691314
H	-0.3052824	-1.4223412	-3.2521805
H	-1.4105530	-0.6707037	-2.0792979
H	-1.6148372	-0.3572231	-3.8411624
H	1.0380148	0.7269423	-6.3351457
H	-0.0147529	-0.4796482	-5.5414380
H	-0.5549829	1.2141352	-5.6910230
H	1.9935662	2.7115666	-3.3069877
H	0.6803807	3.1080687	-4.4639394
H	3.1406421	0.5366649	-4.1854097
H	1.3999125	-2.0122237	-3.9865420
H	2.2233324	-1.4128579	-5.4656360
H	5.4919077	-0.2188501	-1.5169023
H	4.8632382	-1.6757165	-2.3294483
H	4.7121593	-0.0774947	-3.0992475
H	5.6515313	-2.6080788	1.3351638
H	4.6526334	-2.9539952	-0.1092648
H	5.9909797	-1.7669652	-0.1900766
H	5.5600575	0.6620808	0.5221450
H	4.3481809	0.9690762	1.8062642

H	3.8040665	-1.3194827	3.0612947
H	3.7371695	-3.6904873	2.1589177
H	2.1633738	-3.2110261	2.8646403
H	-0.0423685	-0.4485369	5.6230546
H	0.5785185	1.9108597	4.9511573
H	-0.4478509	2.8024177	3.7766568
H	-1.1238546	2.3127406	5.3675754
H	-1.8010399	-0.1748769	5.7657296
H	-1.1881222	-1.4238844	4.6438869
H	-2.8795120	1.6550829	3.6604120
H	-2.5029318	-1.1971497	2.5254439
H	-3.9889747	-0.2132631	2.3743897
H	-4.3647080	3.1756867	0.6269262
H	-4.6434188	1.7010211	1.5808909
H	-3.7632043	3.0777831	2.2886328
H	-8.5503897	0.4934689	0.7903753
H	5.7602955	-0.0902889	2.1390553
H	2.2399822	2.4845506	-5.0649805
H	-3.4907504	-0.8079963	3.9729813
H	2.3562433	-3.2384571	1.0929603
H	3.1772320	-1.9252988	-4.0478761
H	-3.1684430	3.4811089	-4.3740077
H	1.4471022	-0.7588892	3.9282560

MeCbl

Co	0.6017111	0.7719417	-0.1007136
N	1.0849131	0.4597234	-1.8685581
N	2.0211334	-0.3960129	0.4722517
N	-0.2514628	0.8913639	1.6002613
N	-0.7507177	1.8040952	-0.8665022
C	0.0755952	0.8352585	-2.9072549
C	-0.9851643	-0.2767189	-2.9933092
C	0.9514417	0.9731579	-4.2120918
C	0.1940158	0.6826494	-5.5139616
C	1.5990175	2.3717421	-4.3148805
C	2.1093881	-0.0517499	-3.9251015
C	1.9647011	-1.4863654	-4.4714970
C	2.1836074	-0.0367673	-2.4064476
C	3.2797660	-0.5595850	-1.6393839

C	4.5674698	-0.8835500	-2.3709967
C	3.1367852	-0.7763143	-0.2713540
C	4.2002631	-1.3800455	0.6716670
C	5.0473921	-2.5496613	0.1444796
C	5.1191814	-0.2167198	1.1293420
C	3.3054097	-1.8110343	1.8750159
C	2.7517614	-3.2500179	1.8208509
C	2.1618166	-0.8368617	1.7548758
C	1.3493331	-0.4833181	2.8239695
C	0.2242780	0.3385014	2.7473250
C	-0.5815924	0.7704481	3.9666594
C	0.1191011	2.0201976	4.5619464
C	-0.7281038	-0.3105346	5.0457190
C	-1.9087644	1.1959301	3.2815220
C	-2.9808501	0.0914424	3.1873943
C	-1.4461234	1.5542028	1.8799712
C	-2.1848015	2.3441127	1.0099381
C	-3.3972679	3.0706082	1.5496356
C	-1.8248647	2.4049573	-0.3824656
C	-2.5858303	3.1729528	-1.4780288
C	-2.2947721	4.6828799	-1.3080602
C	-4.1112368	2.9066645	-1.5240528
C	-4.5394911	1.4500830	-1.3295838
C	-6.0566512	1.3024619	-1.5065075
O	-6.7686203	2.2484357	-1.8630830
N	-6.5550795	0.0598061	-1.2314065
C	-1.9197557	2.6036277	-2.7809964
C	-1.8988381	3.5483413	-3.9804013
C	-0.5515806	2.1103178	-2.2991508
C	-7.9804062	-0.1098801	-1.0017376
C	-8.4642276	0.4761507	0.3428147
C	-9.9856211	0.4932837	0.4391462
O	-7.9914991	-0.3049373	1.4637896
O	-6.4626819	-0.6300353	3.5030226
O	-5.8113502	1.1531955	1.6094846
P	-6.4577301	-0.1418654	2.0623265
O	-5.6656280	-1.3524543	1.1354831
C	-6.0351926	-2.6987862	1.4121167
C	-5.5864364	-3.6184257	0.2523263

O	-5.5567081	-2.9169000	-0.9675316
C	-4.2286836	-4.2108583	0.7519279
O	-4.3560006	-4.2854061	2.1437664
C	-5.2845753	-3.2737665	2.6349855
C	-6.2137626	-3.9207505	3.6757949
O	-7.3011757	-3.1101319	4.0185912
N	-3.0675053	-3.4399632	0.2981465
C	-1.8515235	-3.9509181	-0.1638598
C	-2.9549936	-2.0573722	0.2572492
N	-1.7827579	-1.6501475	-0.1889823
C	-1.0655947	-2.8037686	-0.4703039
C	0.2276988	-2.9541835	-0.9991313
C	0.7350852	-4.2442212	-1.2212576
C	2.1194306	-4.4101131	-1.8064660
C	-0.0532097	-5.3966345	-0.8882278
C	0.5174907	-6.7790880	-1.1070030
C	-1.3491311	-5.2485043	-0.3556157
H	-5.3363761	-3.5420191	-1.6815528
H	-7.0205657	-2.1460423	3.9543226
H	-5.5829036	-4.2204566	4.5538884
H	-6.5948132	-4.8721290	3.2322578
H	-4.7135052	-2.4412605	3.0980249
H	-7.1338130	-2.7534605	1.5631953
H	-6.2967835	-4.4773621	0.2243689
H	-4.0653218	-5.2404012	0.3696849
H	-3.7935550	-1.4208940	0.5769576
H	1.4520836	-6.9337555	-0.5254055
H	-0.1965512	-7.5701389	-0.8068294
H	0.7822740	-6.9518378	-2.1724450
H	2.5922958	-3.4232372	-1.9791673
H	2.7879578	-4.9967880	-1.1396175
H	2.0983892	-4.9505738	-2.7771366
H	-1.9392183	-6.1405922	-0.0949192
H	0.8273555	-2.0619837	-1.2195604
H	-10.4219172	1.1391493	-0.3490388
H	-10.3955118	-0.5321868	0.3269765
H	-10.2975599	0.8776626	1.4293472
H	-8.5281381	0.4011243	-1.8195977
H	-8.2161114	-1.1935623	-1.0357857

H	-5.9491940	-0.6253048	-0.7627285
H	-4.0166477	0.7580554	-2.0264423
H	-4.3040264	1.0983741	-0.3034484
H	-4.6496654	3.5254829	-0.7829837
H	-4.4884774	3.2677748	-2.5046107
H	-1.2106509	4.9017655	-1.3926021
H	-2.8307389	5.2711164	-2.0787757
H	-2.6329452	5.0462657	-0.3196735
H	-1.5647221	3.0226377	-4.8960971
H	-1.2220247	4.4121958	-3.8247864
H	-2.5151739	1.7119834	-3.0572195
H	0.1976728	2.9306354	-2.3442540
H	-0.5282230	-1.2492670	-3.2482370
H	-1.4789934	-0.4035123	-2.0088870
H	-1.7490644	-0.0444128	-3.7577678
H	0.8769472	0.7733240	-6.3833904
H	-0.2504391	-0.3285999	-5.5411727
H	-0.6265142	1.4121525	-5.6618853
H	2.1681297	2.6312974	-3.3983203
H	0.8467567	3.1639929	-4.4894511
H	3.0490144	0.3612614	-4.3490951
H	1.0841941	-2.0076082	-4.0506336
H	1.8793512	-1.4874418	-5.5755542
H	5.4573645	-0.5907583	-1.7845192
H	4.6717458	-1.9629843	-2.6078606
H	4.6279636	-0.3351952	-3.3282199
H	5.5205019	-3.0677981	1.0031197
H	4.4380080	-3.2905249	-0.4048270
H	5.8673418	-2.2185765	-0.5161705
H	5.6749612	0.2164013	0.2744628
H	4.5300081	0.5981963	1.5973873
H	3.8504301	-1.6689846	2.8316902
H	3.5572802	-4.0054163	1.9040231
H	2.0465184	-3.4120606	2.6592157
H	0.2371970	-0.5001038	5.5585890
H	1.1407048	1.7721828	4.9142653
H	0.2011298	2.8287656	3.8068337
H	-0.4631604	2.4085340	5.4228326
H	-1.4491181	0.0207062	5.8192985

H	-1.0899575	-1.2690483	4.6286171
H	-2.3495431	2.0709669	3.7976378
H	-2.5609860	-0.8255992	2.7278696
H	-3.8282986	0.4336723	2.5653365
H	-3.6586563	3.9530756	0.9442271
H	-4.3002886	2.4168848	1.5907033
H	-3.2121035	3.4323810	2.5789781
H	-8.0577644	1.5075709	0.4266835
H	5.8581288	-0.5814916	1.8723925
H	2.3078793	2.3963047	-5.1676384
H	-3.3957548	-0.1624535	4.1811007
H	2.1886211	-3.4264085	0.8834293
H	2.8555263	-2.0891951	-4.2089285
H	-2.9137095	3.9436735	-4.1867790
H	1.6119276	-0.8961108	3.8070558
C	1.7623177	2.3089450	0.2000877
H	2.1411575	2.2366233	1.2391799
H	2.5991079	2.2569153	-0.5261739
H	1.1751881	3.2383804	0.0667418

ImHCbl

Co	0.5025474	0.7178438	-0.1213641
N	1.0687817	0.4635658	-1.8852066
N	1.9735557	-0.3781937	0.4972990
N	-0.4342210	0.7463335	1.5586047
N	-0.8823339	1.6946099	-0.9245897
C	0.0687657	0.7852433	-2.9422150
C	-0.9269157	-0.3825355	-3.0532948
C	0.9637403	0.9641049	-4.2298759
C	0.2441207	0.6347900	-5.5443150
C	1.5394090	2.3934963	-4.3332006
C	2.1669844	-0.0027730	-3.9137327
C	2.1117176	-1.4395886	-4.4702603
C	2.1989975	0.0068769	-2.3918601
C	3.2975134	-0.4541879	-1.5849838
C	4.6232633	-0.7087382	-2.2769749
C	3.1299894	-0.6710597	-0.2156067
C	4.2149136	-1.1583115	0.7714508
C	5.1995824	-2.2393708	0.2968129

C	4.9851141	0.1054504	1.2394006
C	3.3252672	-1.6662155	1.9487893
C	2.9043424	-3.1483360	1.8787534
C	2.1024787	-0.7981141	1.7858427
C	1.2201522	-0.5217417	2.8252376
C	0.0265835	0.1966369	2.7112877
C	-0.8829080	0.5141349	3.8960000
C	-0.3558553	1.8051503	4.5738823
C	-0.9801169	-0.6117791	4.9346571
C	-2.2061287	0.8294150	3.1444009
C	-3.1185501	-0.3927832	2.9409232
C	-1.7010934	1.2800910	1.7811547
C	-2.4623926	2.0180232	0.8846337
C	-3.7756525	2.5913183	1.3857088
C	-2.0341415	2.1674963	-0.4859568
C	-2.8264738	2.8685605	-1.6088249
C	-2.7934535	4.3959135	-1.3812464
C	-4.2825863	2.3463907	-1.7582616
C	-4.4220813	0.8320736	-1.5426826
C	-5.8145715	0.3241934	-1.9274102
O	-6.2095760	0.3073532	-3.0956697
N	-6.5424050	-0.1006211	-0.8520101
C	-2.0152274	2.4480621	-2.8888322
C	-1.9881561	3.4694202	-4.0246297
C	-0.6443651	2.0272942	-2.3438287
C	-7.9176675	-0.5708290	-0.9477297
C	-8.6741739	-0.3574253	0.3732610
C	-10.1541932	-0.6968808	0.2326034
O	-8.1626883	-1.2037779	1.4298400
O	-6.8909032	-1.3983771	3.6479706
O	-6.2790163	0.5486510	1.8614647
P	-6.7569873	-0.8517459	2.2423465
O	-5.6636524	-1.8490418	1.4033091
C	-5.7110275	-3.2492647	1.6256402
C	-5.4961541	-3.9641409	0.2477665
O	-5.6995050	-3.1436344	-0.8631802
C	-4.0681153	-4.5787288	0.4156024
O	-4.0000619	-4.8885774	1.7928002
C	-4.5233174	-3.7337316	2.4890809

C	-4.8208411	-4.0440819	3.9576848
O	-4.9226373	-2.8686748	4.7198701
N	-2.9904066	-3.6827442	-0.0106174
C	-1.7081579	-4.0847782	-0.3885224
C	-2.9666503	-2.2939978	0.0160531
N	-1.7938129	-1.7822986	-0.3111460
C	-0.9810312	-2.8772916	-0.5847422
C	0.3537051	-2.9284269	-1.0234458
C	0.9569807	-4.1747374	-1.2571003
C	2.3848052	-4.2293515	-1.7531592
C	0.2241852	-5.3873222	-1.0267674
C	0.8951385	-6.7219444	-1.2573796
C	-1.1129923	-5.3405530	-0.5878518
H	-5.7760052	-2.2184750	-0.5205471
H	-5.6927202	-2.3222335	4.3735683
H	-3.9722679	-4.6454985	4.3522864
H	-5.7314640	-4.6947562	4.0185402
H	-3.7610378	-2.9236207	2.4867476
H	-6.6856885	-3.5308438	2.0750707
H	-6.1801188	-4.8393218	0.1945389
H	-3.9395713	-5.5227196	-0.1481179
H	-3.8473405	-1.7133000	0.3146127
H	1.7932001	-6.8471206	-0.6142858
H	0.2108840	-7.5657411	-1.0438934
H	1.2457730	-6.8303987	-2.3064536
H	2.8044251	-3.2085662	-1.8453621
H	3.0443072	-4.8093104	-1.0716503
H	2.4594778	-4.7198274	-2.7476403
H	-1.6667754	-6.2736576	-0.4048487
H	0.9073567	-1.9944281	-1.1716746
H	-10.6434032	-0.0363321	-0.5109257
H	-10.2778476	-1.7498520	-0.0930026
H	-10.6619297	-0.5772890	1.2087338
H	-8.4292299	-0.0372168	-1.7781458
H	-7.9355992	-1.6560284	-1.1976406
H	-6.2354517	0.2074370	0.0978226
H	-3.6997518	0.2791453	-2.1766566
H	-4.1834787	0.5676572	-0.4944505
H	-4.9682773	2.8757088	-1.0673421

H	-4.6321557	2.5966198	-2.7836549
H	-1.7537775	4.7820831	-1.3829977
H	-3.3620449	4.9245201	-2.1719799
H	-3.2456009	4.6673853	-0.4087140
H	-1.5373983	3.0332524	-4.9376712
H	-1.4063211	4.3767088	-3.7642744
H	-2.5100287	1.5362118	-3.2737971
H	0.0660798	2.8830264	-2.3521420
H	-0.4105374	-1.3265061	-3.3013339
H	-1.4286276	-0.5405518	-2.0780983
H	-1.6911769	-0.1952130	-3.8291857
H	0.9328142	0.7643984	-6.4042715
H	-0.1434206	-0.3990971	-5.5782370
H	-0.6150456	1.3168021	-5.7020440
H	2.0744293	2.6922589	-3.4086267
H	0.7519517	3.1467216	-4.5237532
H	3.0951848	0.4600946	-4.3131225
H	1.2479164	-2.0072865	-4.0755517
H	2.0576409	-1.4405103	-5.5763994
H	5.4777779	-0.3282017	-1.6864548
H	4.8154696	-1.7853783	-2.4678072
H	4.6650282	-0.1973650	-3.2558152
H	5.6879411	-2.6965845	1.1812216
H	4.6928095	-3.0465052	-0.2643295
H	6.0074412	-1.8331059	-0.3368944
H	5.5164301	0.5830725	0.3915940
H	4.2912235	0.8555100	1.6730227
H	3.8264733	-1.4756824	2.9214812
H	3.7699360	-3.8318444	1.9788562
H	2.1953689	-3.3744050	2.6989067
H	-0.0331149	-0.7130577	5.5038317
H	0.6855816	1.6742886	4.9324569
H	-0.3823077	2.6651060	3.8717946
H	-0.9935325	2.0661730	5.4433782
H	-1.7821636	-0.3894607	5.6658921
H	-1.2093724	-1.5894479	4.4709055
H	-2.7755597	1.6258465	3.6626344
H	-2.5539052	-1.1963703	2.4282425
H	-3.9850210	-0.1315740	2.3084073

H	-4.1944516	3.3487501	0.7052170
H	-4.5640513	1.8209193	1.5404460
H	-3.6341111	3.0926263	2.3640765
H	-8.5498820	0.7059304	0.6823874
H	5.7363375	-0.1617477	2.0110405
H	2.2635574	2.4507546	-5.1716461
H	-3.5201871	-0.8028463	3.8879211
H	2.3814090	-3.3672168	0.9269253
H	3.0245068	-1.9971567	-4.1821870
H	-3.0149064	3.7879499	-4.2952821
H	1.4754291	-0.9268726	3.8136687
N	1.7772779	2.3703774	0.3202106
C	1.9559640	2.9131528	1.5767854
C	2.8119304	2.7581709	-0.4191814
C	3.1263051	3.6517103	1.5967810
N	3.6526758	3.5417780	0.3196949
H	2.9937901	2.4649039	-1.4579580
H	3.6148375	4.2339894	2.3837263
H	4.5218688	3.9633216	-0.0100248
H	1.2395708	2.7217091	2.3805081

Cartesian coordinates of complexes 21 to 52

21

C	-0.69756549	-0.27153558	0.00000000
H	-0.34256549	-1.31853558	0.00000000
H	-0.34356549	0.25146442	0.90600000
H	-0.34356549	0.25146442	-0.90700000
Br	-2.65656549	-0.27253558	0.00000000
C	-7.05456549	-0.27253558	0.00000000
N	-5.88756549	-0.27253558	0.00000000
H	-8.14156549	-0.27253558	0.00000000

22

C	-0.43539323	-0.17790262	0.00000000
H	-0.07839323	-1.22390262	0.00000000
H	-0.07739323	0.34509738	0.90600000

H	-0.07739323	0.34509738	-0.90600000
Br	-2.40839323	-0.17690262	0.00000000
N	-5.33239323	-0.17590262	0.00000000
H	-5.73539323	0.77109738	0.00000000
H	-5.72639323	-0.65290262	0.82100000
H	-5.72639323	-0.65290262	-0.82200000

23

C	0.18258426	-0.44943820	0.00000000
H	0.58158426	-1.47943820	0.02500000
H	0.45058426	0.08856180	0.92700000
H	0.57058426	0.09056180	-0.88300000
Br	-1.77541574	-0.53943820	-0.12800000
O	-4.74841574	-0.42143820	0.05600000
C	-4.89741574	-0.73443820	1.42600000
H	-5.93041574	-0.51743820	1.79400000
H	-4.69541574	-1.81643820	1.54500000
H	-4.17441574	-0.16943820	2.06500000
C	-4.97141574	0.94656180	-0.21800000
H	-4.83541574	1.09656180	-1.30700000
H	-6.00541574	1.26656180	0.06300000
H	-4.24641574	1.60556180	0.32100000

24

C	-0.18258426	0.08426966	0.00000000
H	0.44241574	-0.82573034	0.03400000
H	-0.01658426	0.69726966	0.90500000
H	0.04241574	0.67126966	-0.90900000
Br	-2.06958426	-0.46473034	-0.05100000
O	-5.03658426	-1.03973034	-0.09700000
C	-5.67458426	0.00326966	-0.02100000
H	-6.80358426	0.02426966	-0.02400000
N	-5.13758426	1.25226966	0.07400000
H	-4.11658426	1.34926966	0.08300000
H	-5.72658426	2.08126966	0.13200000

25

C	-0.25749062	-0.26217228	0.00000000
H	0.08850938	-1.31117228	0.00000000

H	0.08850938	0.26182772	0.90800000
H	0.08850938	0.26182772	-0.90900000
C	-6.79249062	-0.26317228	0.00000000
N	-5.62749062	-0.26317228	0.00000000
H	-7.87949062	-0.26317228	0.00000000
I	-2.43449062	-0.26217228	0.00000000

26

C	2.34550545	-0.38389513	0.00000000
H	2.69550545	-1.43189513	0.00000000
H	2.69650545	0.14010487	0.90700000
H	2.69650545	0.14010487	-0.90800000
N	-2.77549455	-0.38189513	0.00000000
H	-3.16649455	0.56810487	0.00000000
H	-3.16049455	-0.85989513	0.82500000
H	-3.16049455	-0.85989513	-0.82500000
I	0.14750545	-0.38189513	0.00000000

27

C	1.14700367	-0.63670411	0.00000000
H	1.48700367	-1.68770411	-0.00900000
H	1.51900367	-0.11470411	0.89900000
H	1.47200367	-0.11470411	-0.91800000
O	-4.00899633	-0.46470411	0.25900000
C	-4.38199633	-0.82570411	1.57500000
H	-5.49099633	-0.79270411	1.71400000
H	-4.03099633	-1.86070411	1.74900000
H	-3.91499633	-0.15870411	2.34100000
C	-4.39299633	0.85329589	-0.08300000
H	-4.04499633	1.04329589	-1.11700000
H	-5.50199633	0.98429589	-0.04400000
H	-3.92899633	1.61329589	0.59200000
I	-1.03599633	-0.62570411	0.05500000

28

C	0.41666664	-0.32768727	-0.00428966
H	0.98866664	-1.27268727	0.01471034
H	0.61366664	0.26531273	0.90671034
H	0.65866664	0.25531273	-0.91128966

O	-4.69033336	-1.32168727	-0.09228966
C	-5.40333336	-0.32468727	-0.02028966
H	-6.52833336	-0.39468727	-0.02628966
N	-4.96133336	0.95831273	0.07271034
H	-3.95233336	1.13731273	0.08471034
H	-5.61233336	1.74031273	0.12971034
I	-1.71133336	-0.83068727	-0.05228966

29

C	-2.09269648	-0.00936330	0.00000000
H	-1.73869648	-1.05636330	0.00000000
H	-1.70569648	0.50963670	0.89800000
H	-1.70569648	0.51063670	-0.89800000
As	-4.08669648	0.12063670	-0.00100000
H	-4.31069648	-0.92836330	-1.10500000
H	-4.31069648	-0.92836330	1.10400000
C	-7.60269648	0.35863670	0.00000000
N	-7.89269648	-0.77236330	0.00000000
H	-7.32369648	1.40963670	0.00000000

30

C	-0.05149812	-0.25280898	0.00000000
H	0.09150188	-1.34980898	-0.02800000
H	0.48450188	0.16019102	0.87800000
H	0.39050188	0.19319102	-0.91300000
As	-1.98849812	0.28919102	0.11300000
H	-2.46049812	-0.68280898	-0.98300000
H	-2.34149812	-0.71280898	1.22600000
N	-5.10649812	0.24419102	0.27600000
H	-5.60949812	0.03119102	-0.59600000
H	-5.28749812	1.24019102	0.46200000
H	-5.60549812	-0.26980898	1.01600000

31

C	0.24812732	-0.40262172	0.00000000
H	0.27312732	-1.50562172	-0.07700000
H	0.75012732	-0.09262172	0.93800000
H	0.80512732	0.03537828	-0.85100000
As	-1.61587268	0.33537828	-0.00300000

H	-2.10487268	-0.51262172	-1.19000000
H	-2.16887268	-0.67962172	1.01300000
O	-4.72287268	0.58637828	0.00400000
C	-4.95587268	1.73737828	-0.78200000
H	-4.69787268	1.48637828	-1.82900000
H	-6.02687268	2.05837828	-0.74500000
H	-4.32387268	2.60137828	-0.46000000
C	-5.01887268	0.78937828	1.36900000
H	-6.09187268	1.06337828	1.52900000
H	-4.81387268	-0.15962172	1.90200000
H	-4.38787268	1.59437828	1.82300000

32

C	-0.02340824	-0.38389513	0.00000000
H	0.37159176	-1.41589513	-0.01600000
H	0.35559176	0.13810487	0.90000000
H	0.33859176	0.15910487	-0.89500000
As	-2.02540824	-0.33189513	0.01600000
H	-2.22840824	-1.35989513	-1.10300000
H	-2.21140824	-1.38189513	1.11900000
O	-5.07040824	-1.24589513	-0.02200000
C	-5.78740824	-0.25189513	-0.00800000
H	-6.91540824	-0.31889513	-0.01500000
N	-5.34540824	1.03510487	0.02000000
H	-4.33140824	1.20510487	0.02600000
H	-5.99440824	1.82210487	0.02700000

33

C	0.83801492	-0.12172284	0.00000000
H	0.97101492	-1.21872284	0.00000000
H	1.31801492	0.31027716	0.90000000
H	1.31801492	0.31027716	-0.90100000
H	-1.69498508	-0.72672284	-1.23600000
H	-1.69498508	-0.72672284	1.23500000
C	-5.84198508	0.60827716	0.00000000
N	-4.73798508	0.23127716	0.00000000
H	-6.86998508	0.96027716	0.00000000
Sb	-1.30698508	0.42527716	0.00000000

34

C	1.50280888	-0.41198501	0.00000000
H	1.56980888	-1.51598501	-0.01600000
H	2.05780888	-0.02498501	0.87800000
H	1.96880888	-0.00298501	-0.91900000
H	-1.11719112	-0.83498501	-1.11100000
H	-0.98519112	-0.85298501	1.37300000
N	-3.68419112	0.17201499	0.27800000
H	-4.09619112	-0.14898501	-0.60800000
H	-4.14019112	1.06901499	0.49600000
H	-4.00519112	-0.49198501	0.99600000
Sb	-0.61119112	0.28201499	0.11500000

35

C	2.64513090	-0.96441946	0.00000000
H	2.53113090	-2.06441946	-0.01200000
H	3.20013090	-0.66141946	0.90900000
H	3.21913090	-0.64341946	-0.89100000
H	0.04113090	-1.00141946	-1.23300000
H	0.04913090	-0.97741946	1.24300000
O	-2.47286910	0.38158054	0.09300000
C	-2.97286910	1.41758054	-0.72700000
H	-2.51986910	1.29858054	-1.73100000
H	-4.08586910	1.36758054	-0.82900000
H	-2.70886910	2.43058054	-0.33700000
C	-2.97486910	0.43158054	1.41300000
H	-4.08586910	0.31458054	1.44100000
H	-2.51486910	-0.40441946	1.97600000
H	-2.71786910	1.39058054	1.92700000
Sb	0.67413090	0.04658054	-0.00900000

36

C	1.29681639	0.32771535	0.00000000
H	1.62881639	-0.72728465	-0.01000000
H	1.69081639	0.83071535	0.90500000
H	1.68781639	0.84671535	-0.89700000
H	-1.09518361	-0.72428465	-1.23900000
H	-1.08718361	-0.72828465	1.24700000
O	-4.02518361	-0.35328465	-0.02500000

C	-4.83718361	0.56471535	-0.00700000
H	-5.95218361	0.38471535	-0.01800000
N	-4.52818361	1.88971535	0.02600000
H	-3.54118361	2.16771535	0.03500000
H	-5.25318361	2.60671535	0.03900000
Sb	-0.91218361	0.45971535	0.00500000

37

Br	2.01779012	-0.31835206	0.00000000
C	-2.06620988	-0.31835206	0.00000000
N	-0.90120988	-0.31835206	0.00000000
H	-3.15420988	-0.31835206	0.00000000
C	3.82279012	-0.31735206	0.00000000
N	4.99979012	-0.31735206	0.00000000

38

Br	1.40917593	-0.48689138	0.00000000
N	-1.28382407	-0.48689138	0.00000000
H	-1.67682407	0.46410862	0.00000000
H	-1.67282407	-0.96389138	0.82400000
H	-1.67282407	-0.96389138	-0.82500000
C	3.24417593	-0.48789138	0.00000000
N	4.42117593	-0.48789138	0.00000000

39

Br	2.87921328	-0.58988763	0.00000000
O	0.19721328	-0.42388763	0.22500000
C	-0.24378672	-0.81688763	1.51500000
H	-1.35678672	-0.86788763	1.56600000
H	0.16921328	-1.82488763	1.71400000
H	0.11121328	-0.11788763	2.31000000
C	-0.25578672	0.86611237	-0.15200000
H	0.14721328	1.07711237	-1.16100000
H	-1.36978672	0.91111237	-0.19200000
H	0.10221328	1.65711237	0.55000000
C	4.69121328	-0.64488763	-0.11400000
N	5.86521328	-0.67688763	-0.18600000

40

Br	1.78355489	0.04650685	-0.02395882
O	-0.84744511	-0.51749315	-0.06095882
C	-1.68744511	0.37850685	-0.00695882
H	-2.79044511	0.15550685	-0.01295882
N	-1.42144511	1.70850685	0.06404118
H	-0.45044511	2.03250685	0.07604118
H	-2.17144511	2.39850685	0.10804118
C	3.54155489	0.51750685	0.00804118
N	4.67455489	0.83650685	0.03004118

41

C	1.80243433	-0.75842695	0.00000000
N	2.96643433	-0.75842695	0.00000000
H	0.71443433	-0.75842695	0.00000000
I	5.86443433	-0.75842695	0.00000000
C	7.89843433	-0.75742695	0.00000000
N	9.07543433	-0.75742695	0.00000000

42

N	0.13576778	-0.06554307	0.00000000
H	-0.25023222	0.88845693	0.00000000
H	-0.24723222	-0.54354307	0.82600000
H	-0.24723222	-0.54354307	-0.82700000
I	2.85776778	-0.06554307	0.00000000
C	4.93076778	-0.06654307	0.00000000
N	6.10776778	-0.06654307	0.00000000

43

O	0.93164788	-0.75842695	0.00000000
C	0.41264788	-1.19942695	1.24900000
H	-0.69035212	-1.34742695	1.19300000
H	0.89364788	-2.16842695	1.48400000
H	0.63264788	-0.47642695	2.06900000
C	0.39764788	0.48957305	-0.42400000
H	0.86664788	0.73657305	-1.39600000
H	-0.70635212	0.42657305	-0.56100000
H	0.62064788	1.30557305	0.30300000
I	3.63664788	-0.97642695	-0.27000000
C	5.66864788	-1.10842695	-0.46400000

N	6.83764788	-1.17942695	-0.57500000

44

O	0.22940073	-0.64606741	0.00000000
C	-0.63559927	0.23293259	0.04700000
H	-1.72759927	-0.02806741	0.01900000
N	-0.40659927	1.56493259	0.13600000
H	0.55440073	1.91793259	0.16600000
H	-1.17659927	2.23293259	0.16900000
I	2.89440073	-0.15606741	0.03700000
C	4.89640073	0.29793259	0.06200000
N	6.04140073	0.57093259	0.07600000

45

As	2.13014966	-0.10299625	0.00000000
H	1.72114966	-1.08699625	-1.10800000
H	1.72114966	-1.08699625	1.10800000
C	-2.05385034	0.06500375	0.00000000
N	-0.90285034	-0.11899625	0.00000000
H	-3.12785034	0.23500375	0.00000000
C	3.97114966	-0.71999625	0.00000000
N	5.11914966	-0.97299625	0.00000000

46

As	1.87734069	-0.71161048	0.00000000
H	1.47434069	-1.68361048	-1.11900000
H	1.59334069	-1.74461048	1.10100000
N	-0.91965931	-0.68061048	0.14700000
H	-1.38365931	-0.95261048	-0.73100000
H	-1.24165931	0.27438952	0.35300000
H	-1.32465931	-1.28061048	0.87900000
C	3.76434069	-1.26961048	-0.11600000
N	4.92034069	-1.47361048	-0.18400000

47

As	2.02715342	-0.73033707	0.00000000
H	1.45515342	-1.47233707	-1.21600000
H	1.47515342	-1.77533707	0.98200000
O	-0.73284658	-0.26633707	0.05200000

C	-1.13584658	0.77666293	-0.81900000
H	-0.63984658	0.60866293	-1.79500000
H	-2.24084658	0.77566293	-0.97600000
H	-0.83884658	1.78166293	-0.43500000
C	-1.30684658	-0.16233707	1.34300000
H	-2.42084658	-0.22833707	1.30300000
H	-0.92484658	-1.00733707	1.94800000
H	-1.03284658	0.79466293	1.84900000
C	3.74215342	-1.64633707	-0.13800000
N	4.83115342	-2.08233707	-0.20600000

48

As	3.01966271	-0.74906366	0.00000000
H	2.90866271	-1.79406366	-1.11400000
H	2.90266271	-1.79306366	1.11500000
O	0.33966271	-1.51206366	-0.01500000
C	-0.58133729	-0.69806366	-0.00400000
H	-1.65933729	-1.02206366	-0.02000000
N	-0.43933729	0.65193634	0.02900000
H	0.49966271	1.05993634	0.04300000
H	-1.24833729	1.27193634	0.03700000
C	4.97066271	-0.80306366	0.00500000
N	6.14166271	-0.70106366	0.00700000

49

H	0.80056174	-0.29962546	0.00000000
H	0.80056174	-0.29962546	2.47100000
C	-2.97943826	0.95437454	1.23600000
N	-1.82143826	0.82437454	1.23600000
H	-4.06043826	1.07337454	1.23600000
Sb	1.20356174	0.84137454	1.23600000
C	3.26056174	0.15937454	1.23600000
N	4.40756174	-0.09862546	1.23600000

50

H	0.45411982	-0.24344569	0.00000000
H	0.58711982	-0.30544569	2.48300000
N	-1.94488018	0.83355431	1.39100000
H	-2.37288018	0.52155431	0.50900000

H	-2.36288018	1.74955431	1.60600000
H	-2.27988018	0.18555431	2.11800000
Sb	0.87611982	0.87455431	1.25100000
C	2.96611982	0.19955431	1.12700000
N	4.11611982	-0.03944569	1.06800000

51

H	0.90355799	-0.78644944	-0.01029519
H	1.02255799	-0.80944944	2.46470481
O	-1.14144201	0.80955056	1.39370481
C	-1.74944201	1.68855056	0.46070481
H	-1.12744201	1.68955056	-0.45529519
H	-2.77844201	1.34955056	0.19270481
H	-1.81444201	2.73155056	0.85270481
C	-1.85044201	0.71055056	2.61770481
H	-2.87944201	0.30955056	2.46170481
H	-1.29244201	0.01055056	3.27070481
H	-1.93144201	1.69655056	3.13370481
Sb	1.61655799	0.21355056	1.20470481
C	3.44555799	-0.95544944	1.10570481
N	4.49255799	-1.48744944	1.04470481

52

H	1.01591753	-0.41198501	0.00000000
H	1.01391753	-0.38298501	2.48400000
O	-1.60008247	0.11901499	1.21700000
C	-2.58608247	0.85601499	1.22400000
H	-3.63008247	0.43901499	1.19100000
N	-2.56008247	2.21101499	1.26900000
H	-1.66408247	2.70301499	1.30100000
H	-3.42208247	2.75801499	1.27100000
Sb	1.12091753	0.79201499	1.22800000
C	3.29591753	0.64101499	1.23400000
N	4.47091753	0.68401499	1.23800000

TUDDEE

C	-0.51966289	0.75842695	0.00000000
H	-0.17366289	0.00542695	-0.56000000
C	-1.58766289	-3.84257305	-0.99000000

H	-1.06466289	-4.19257305	-0.23800000
H	-1.01666289	-3.77857305	-1.78400000
H	-2.33766289	-4.44657305	-1.17500000
C	0.14033711	0.67942695	1.38600000
H	0.51233711	-0.21257305	1.60200000
H	-0.43166289	1.01442695	2.12100000
C	1.20033711	1.70142695	0.89700000
H	1.28033711	2.47942695	1.51900000
C	0.30033711	2.02742695	-0.32400000
H	-0.21166289	2.86942695	-0.23100000
H	0.77233711	1.98942695	-1.19300000
C	2.44333711	-0.53057305	-0.33800000
C	2.42333711	-1.74057305	0.34000000
H	2.56233711	-1.76457305	1.27900000
C	2.19733711	-2.92057305	-0.36900000
H	2.18533711	-3.75557305	0.08500000
C	1.99133711	-2.87057305	-1.74500000
H	1.84333711	-3.67357305	-2.22900000
C	2.00133711	-1.65157305	-2.41300000
H	1.86033711	-1.62657305	-3.35200000
C	2.21433711	-0.47357305	-1.71400000
H	2.20333711	0.36242695	-2.16500000
C	-3.00766289	3.39642695	1.08500000
C	-2.92666289	4.08742695	-0.08700000
C	-2.74366289	3.41442695	-1.34600000
C	-3.11466289	5.59142695	-0.02700000
H	-4.06366289	5.80742695	-0.14500000
H	-2.59166289	6.01442695	-0.74000000
H	-2.81066289	5.92442695	0.84300000
C	-3.46066289	4.00142695	2.41000000
H	-3.05866289	4.90742695	2.53700000
C	-2.86266289	3.00142695	3.41600000
C	-2.82766289	1.75042695	2.57500000
C	-5.00566289	4.09242695	2.38800000
H	-5.32637889	4.48622011	1.44621011
H	-5.42242951	3.11681112	2.52720963
C	-1.41166289	3.40842695	3.71700000
H	-1.40666289	4.22242695	4.26300000
H	-0.93866289	3.57842695	2.87500000

H	-0.96266289	2.68642695	4.20300000
C	-2.77266289	4.08642695	-2.72800000
C	-1.44866289	4.85642695	-2.92200000
H	-1.34766289	5.51942695	-2.20700000
H	-1.45966289	5.31242695	-3.79000000
H	-0.69666289	4.22842695	-2.89300000
C	-3.99866289	5.01242695	-2.93000000
H	-3.85379784	5.92045610	-2.38282801
H	-4.10869348	5.23665519	-3.97043980
C	-2.86666289	2.85042695	-3.67300000
H	-3.83466289	2.67242695	-3.85000000
C	-2.31866289	1.68642695	-2.83300000
H	-1.33966289	1.58142695	-3.00200000
C	-2.15666289	3.01042695	-5.03600000
H	-1.11558674	3.19867276	-4.87592506
H	-2.27320319	2.11268613	-5.60642069
C	-2.32666289	-2.06157305	0.64900000
C	-2.11966289	-2.47057305	-0.63900000
C	-2.42866289	-1.60257305	-1.74200000
C	-2.78666289	0.48042695	3.10300000
H	-2.77366289	0.39442695	4.05000000
C	-2.76266289	-0.68157305	2.35200000
C	-2.96166289	-2.03857305	2.96200000
H	-2.49966289	-2.09757305	3.84600000
C	-2.25166289	-2.94857305	1.90800000
C	-2.92166289	-4.32057305	1.76600000
H	-3.76666289	-4.22257305	1.27900000
H	-3.09966289	-4.69057305	2.65600000
H	-2.32866289	-4.92457305	1.27300000
C	-0.74866289	-3.07857305	2.28500000
H	-0.18660566	-3.35883694	1.41871840
H	-0.39101949	-2.14002496	2.65394267
C	-2.65566289	-2.08857305	-3.15900000
H	-1.87266289	-2.65557305	-3.41500000
C	-2.57366289	-0.76057305	-3.98600000
C	-3.00866289	0.30642695	-2.90500000
C	-4.52966289	0.47442695	-2.85200000
H	-4.76566289	1.07642695	-2.11600000
H	-4.94966289	-0.39957305	-2.70700000

H	-4.84566289	0.85242695	-3.69900000
C	-3.92466289	-2.96057305	-3.27100000
H	-4.09515054	-3.46323930	-2.34193563
H	-4.76524187	-2.33934168	-3.49990732
C	-1.08966289	-0.55957305	-4.41300000
H	-0.97958597	0.39620493	-4.88126438
H	-0.46023983	-0.60808982	-3.54907134
C	-4.48066289	-2.31457305	3.12000000
H	-4.96072399	-2.22292363	2.16813776
H	-4.62575719	-3.30471297	3.49877510
C	-3.47166289	-0.71357305	-5.23100000
H	-3.13166289	-1.34057305	-5.90300000
H	-3.46966289	0.19442695	-5.60000000
H	-4.38566289	-0.96357305	-4.98300000
C	-3.62966289	2.80842695	4.72500000
H	-3.73666289	3.67442695	5.17100000
H	-3.13066289	2.20042695	5.30800000
H	-4.51166289	2.42842695	4.53300000
N	-2.79966289	2.03242695	1.24800000
N	-2.59266289	-0.73557305	1.01500000
N	-2.59466289	-0.32657305	-1.61900000
N	-2.54066289	2.13442695	-1.42200000
O	3.54133711	1.85242695	-0.26900000
O	3.32833711	0.56742695	1.84400000
S	2.77833711	0.95542695	0.56500000
Co	-2.48866289	0.78342695	-0.12600000
O	-0.68666289	0.48242695	7.77700000
O	-1.55466289	-1.69757305	7.84300000
O	-2.64366289	0.03542695	9.00100000
O	-2.69766289	-0.02257305	6.66000000
Cl	-1.89566289	-0.30457305	7.81100000
C	-8.37266289	1.70142695	0.89700000
H	-8.36598684	2.12839147	1.87809975
C	-7.12966289	-0.53057305	-0.33800000
C	-7.14966289	-1.74057305	0.34000000
H	-7.01066289	-1.76457305	1.27900000
C	-7.37566289	-2.92057305	-0.36900000
H	-7.38766289	-3.75557305	0.08500000
C	-7.58166289	-2.87057305	-1.74500000

H	-7.73066289	-3.67357305	-2.22900000
C	-7.57266289	-1.65157305	-2.41300000
H	-7.71266289	-1.62657305	-3.35200000
C	-7.35866289	-0.47357305	-1.71400000
H	-7.36966289	0.36242695	-2.16500000
O	-6.03166289	1.85242695	-0.26900000
O	-6.24566289	0.56742695	1.84400000
S	-6.79466289	0.95542695	0.56500000
H	-5.33622116	4.73715690	3.17537191
H	-4.90280032	-1.60637407	3.80202214
H	-0.63414764	-3.82631037	3.04175303
H	-3.79313918	-3.68352070	-4.04878416
H	-0.80948483	-1.32872422	-5.10206221
H	-2.58952923	3.83072074	-5.56952116
H	-4.88003439	4.51956054	-2.57621057
H	-9.13880766	0.95687886	0.83724778
H	-8.56350481	2.46827017	0.17559108
